# Supplementary material for: Ucenprubart is an agonistic antibody to CD200R with the potential to treat inflammatory skin disease: preclinical development and a phase 1 clinical study
Source: Nat Commun. 2025 May 1;16:4082. doi: 10.1038/s41467-025-59147-w (PMC12046042; doi:10.1038/s41467-025-59147-w)
Supplement: Supplementary file 1 — Supplementary Information [file 41467_2025_59147_MOESM1_ESM.pdf]

Supplementary Information

Ucenprubart is an agonistic antibody to CD200R with the potential to treat inflammatory skin disease: Preclinical development and a phase 1 clinical study

Anja Koester[1,*], Derrick R. Witcher[2], Mark Lee[3], Stephen J. Demarest[4,†], Scott Potter[1], Katie Werle[1,‡], Scott Bauer[2], Diana Ruiz[4], Laurent Malherbe[5], Josh Poorbaugh[2], Andrew Glasebrook[1,§], Christoph Preuss[2], Gourab Datta[2], Ziqiao Wang[2,¶], Jack Knorr[2], David Manner[2], Dipak Patel[1], Carsten Schmitz[1], Paul Klekotka[1], & Ajay Nirula[6]

[1]Lilly Biotechnology Center, San Diego, Eli Lilly and Company, Immunology Research, San Diego, CA, USA. [2]Lilly Research Laboratories, Eli Lilly and Company, Biotechnology, Indianapolis, IN, USA. [3]Progressive Clinical Research, San Antonio, TX, USA. [4]Lilly Biotechnology Center, San Diego, Eli Lilly and Company, Biotechnology, San Diego, CA, USA. [5]Lilly Research Laboratories, Eli Lilly and Company, Toxicology, Indianapolis, IN, USA. [6]Recludix Pharma, San Diego, CA, USA.

*Correspondence: Anja Koester, Lilly Biotechnology Center, San Diego, Immunology Research, 10290 Campus Point Drive, San Diego, CA 92121, USA. E-mail: koestera@lilly.com

Affiliations at submission: †Tentarix Biotherapeutics, San Diego, CA, USA; ‡Arcturus Therapeutics, San Diego, CA, USA; §Toralgen, Indianapolis, IN, USA; ¶Department of Biostatistics, Bloomberg School of Public Health, Johns Hopkins University, Baltimore, MD, USA.

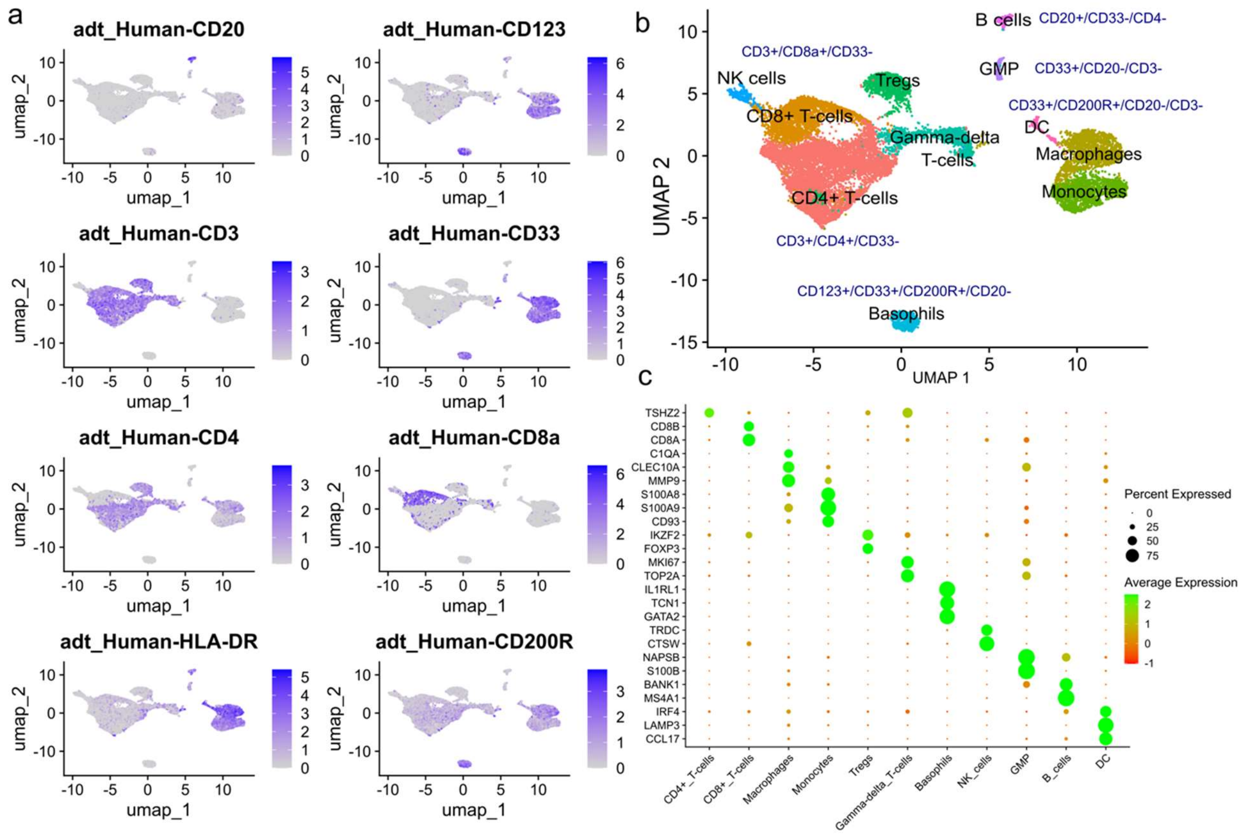

Supplementary Fig. 1 | Additional data for CITE-seq. a, Overview of CITE-seq panel including 8 oligonucleotide-labeled antibodies to improve cell type annotations. **b**, Surface expression of CITE-seq antibodies overlaps with 5 broad cell type clusters (myeloid cells, CD4^{+} T cells, B cells, basophils, and GMP cells) determined based on Uniform Manifold Approximation and Projection (UMAP) projections from single-cell RNA-sequencing data. **c**, Dot plots for differentially expressed genes in each of the 11 identified cell clusters identified by the FindMarkers function in Seurat. CITE-seq, Cellular Indexing of Transcriptomes and Epitopes by Sequencing; DC, dendritic cells; GMP, granulocyte–monocyte progenitors; NK, natural killer; Tregs, regulatory T cells.

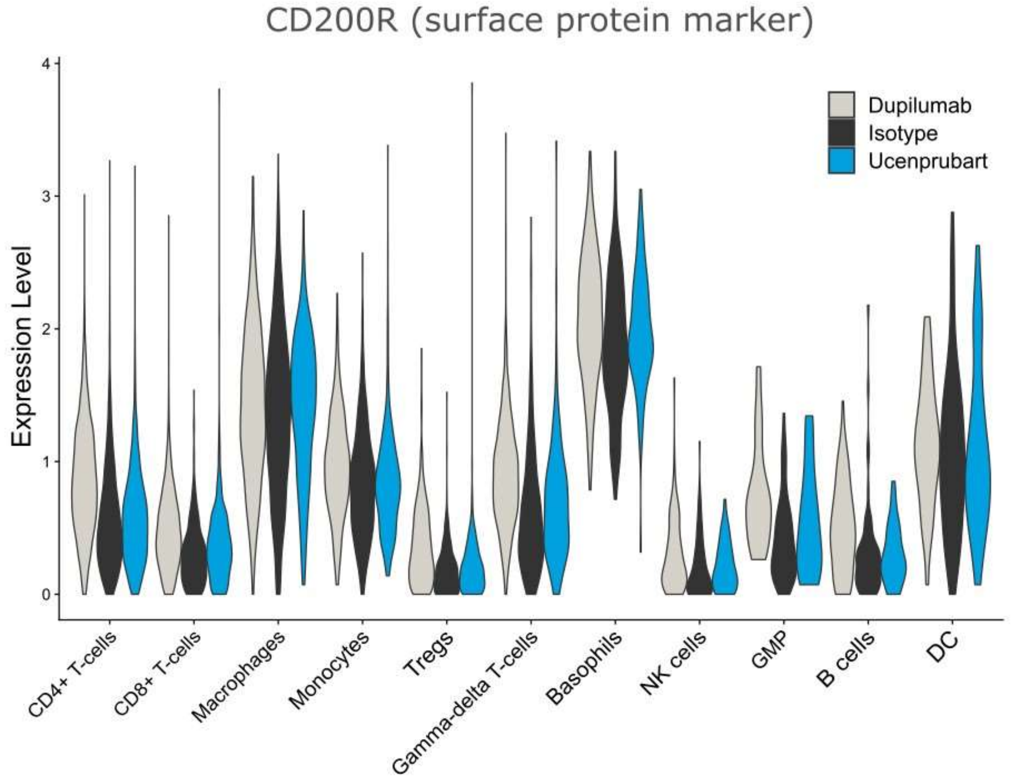

Supplementary Fig. 2 | Surface protein expression of CD200R in response to ucenprubart and dupilumab treatment. No significant differences in human CD200R surface protein expression were observed after ucenprubart or dupilumab treatment when compared to isotype controls. DC, dendritic cells; GMP, granulocyte–monocyte progenitors; NK, natural killer; Tregs, regulatory T cells. Source data are provided as a Source Data file.

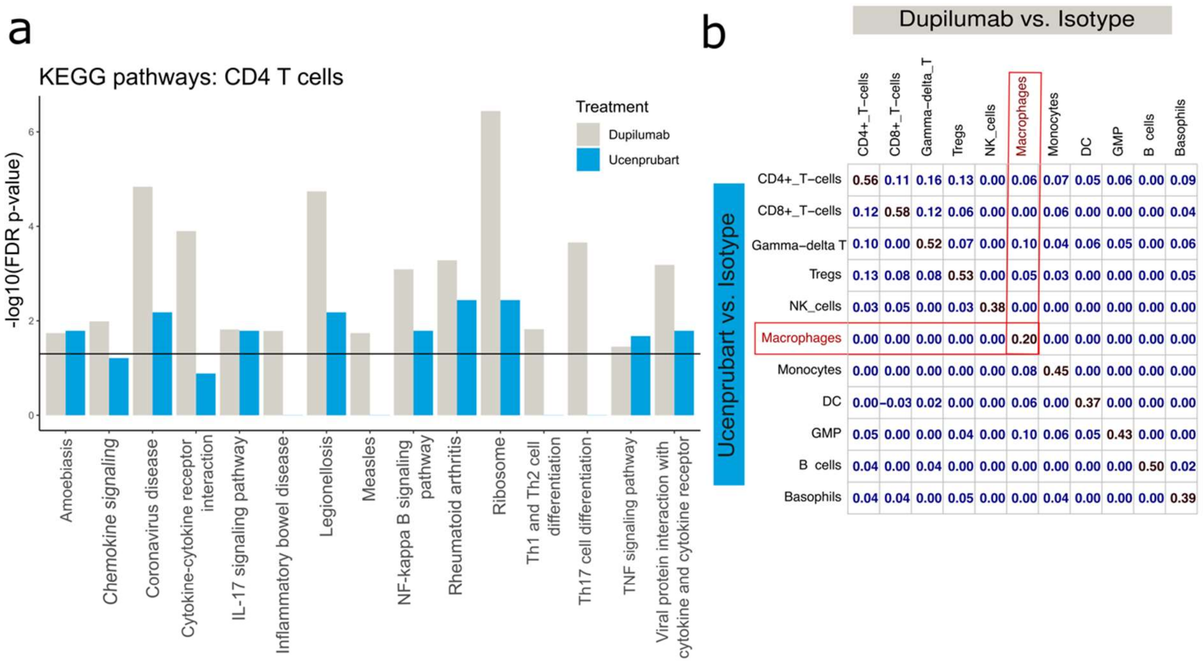

Supplementary Fig. 3 | Analysis of KEGG pathways with dupilumab. a, Analysis of KEGG pathways in $CD4^+$ T cells treated with dupilumab revealed several enriched pathways (false discovery rate [FDR]-adjusted $p < 0.05$) linked to immune function and the Th1 and Th2 response. **b**, Comparison of the number of genes differentially expressed between treatments confirmed the cell type-specific differences in $CD4^+$ T cells and macrophages. Significant positive correlations (Pearsons $p < 0.05$, Pearson R > 0.5) in gene expression changes were observed for T-cell clusters but not macrophages after ucenprubart and dupilumab treatment (Pearson $p = 0.08$; Pearson $r = 0.2$ between treatments). DC, dendritic cells; GMP, granulocyte–monocyte progenitors; IL, interleukin; NK, natural killer; Tregs, regulatory T cells. Source data are provided as a Source Data file.

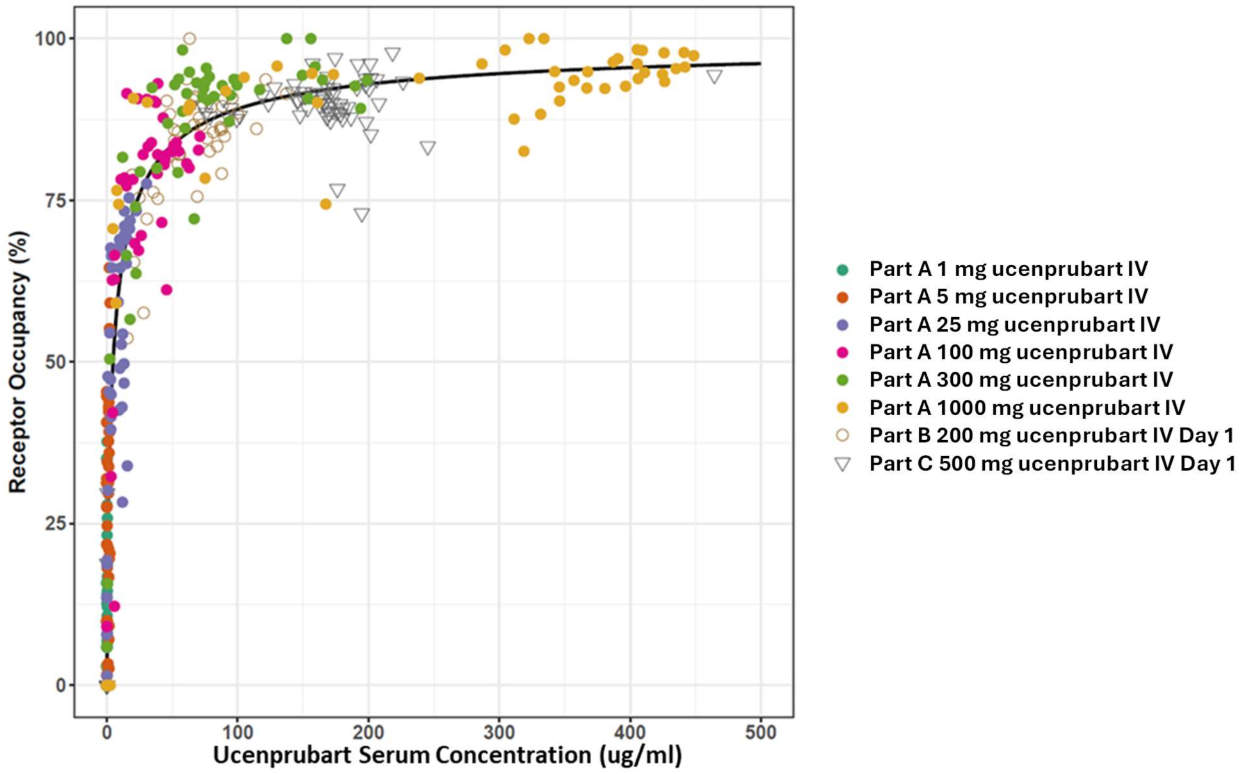

Supplementary Fig. 4 | Receptor occupancy (RO) in whole blood following single-ascending ucenprubart doses. The solid black line represents the median exposure–response curve; symbols represent observed data. Target engagement was assessed using a whole blood assay measuring the binding of ucenprubart to CD200R expressed on neutrophils (CD45 + CD66 + CD14low) by flow cytometry. Briefly, a single draw of heparinized blood was collected from individuals at baseline and subsequent time points in the study. Blood was aliquoted into 3 separate 12 × 75-mm test tubes. Tube 1 was used as the fluorescent minus1 control for the AF647-labeled ucenprubart reagent, while Tube 3 was inoculated with a saturating concentration of unlabeled ucenprubart. All 3 tubes were incubated for 2 hours at 37 °C/5% carbon dioxide in the incubator. Following blocking, antibody panels consisting of CD66 FITC (REA899; Miltenyi, Cat. #130-114-480), ucenprubart (clone P1F9) AF647, CD45 BV421 (Clone HI30; BioLegend, Cat. #557923), CD14 AF700 (Clone M5E2; BD, Cat. #557923) and live/dead yellow (ThermoFisher, Cat. #MT35060CI) were added and incubated in the dark for 60 minutes. Cells were then treated with BD FACS Lysing Solution (BD, Cat. #349202), washed, and read on a BD SORP FACSCanto II. Approximately 250,000 events were collected for each tube. Receptor occupancy was calculated as the mean fluorescence intensity values for each patient at each time point with the corresponding pre-dose sample set as 0% RO control and the corresponding ucenprubart saturated sample at each time point set as 100% control. The formula was as follows: (pre-dose sample - sample at each time point) / (pre-dose sample - saturated sample at each time point) * 100%. P1D5 is a CD200R detecting antibody not competing with ucenprubart and was used to determine total CD200R available. Cat., catalog. Source data are provided as a Source Data file.

Supplementary Fig. 5 | Gating strategies for flow cytometry figures.

a.

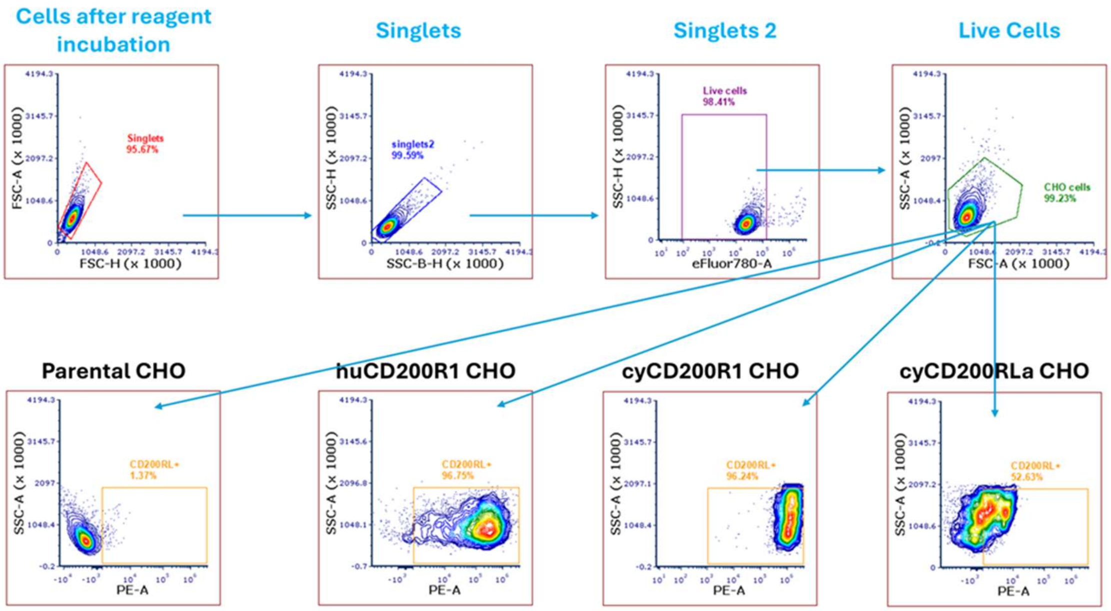

b.

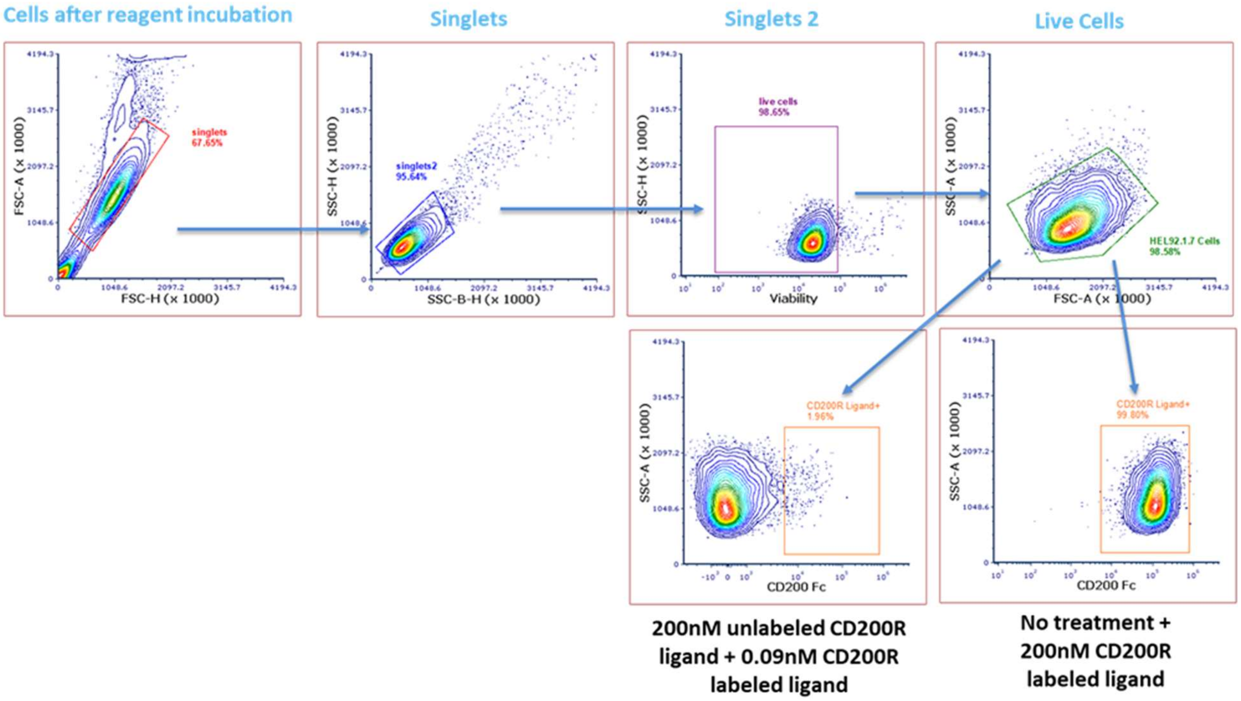
Cells after reagent incubation
Singlets
Singlets 2
Live Cells
singlets
67.65%
FSC-A (x 1000)
FSC-H (x 1000)
singlets2
95.64%
SSC-H (x 1000)
SSC-B-H (x 1000)
live cells
98.65%
Viability
HEL92.1.7 Cells
98.58%
SSC-A (x 1000)
FSC-A (x 1000)
CD200R Ligand+
1.96%
CD200R Ligand+
99.80%
CD200 Fc
200nM unlabeled CD200R ligand + 0.09nM CD200R labeled ligand
No treatment + 200nM CD200R labeled ligand

c.

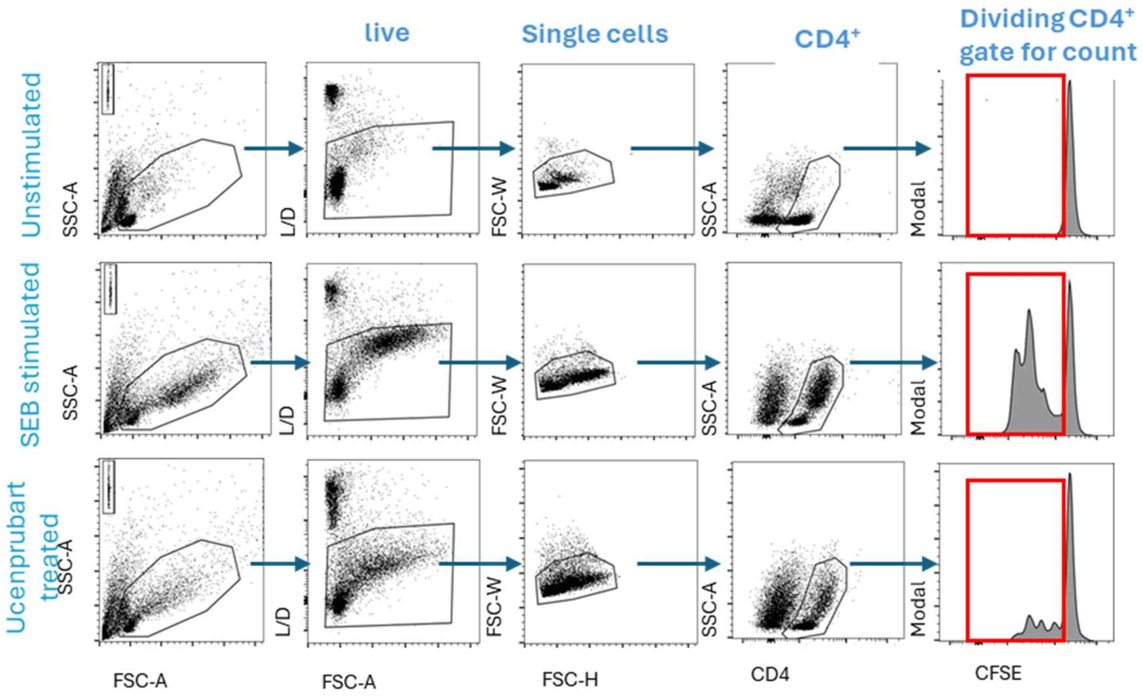

live
Single cells
CD4+
Dividing CD4+ gate for count
Unstimulated
SEB stimulated
Ucenprubart treated
SSC-A
L/D
FSC-W
SSC-A
Modal
FSC-A
FSC-A
FSC-H
CD4
CFSE

d.

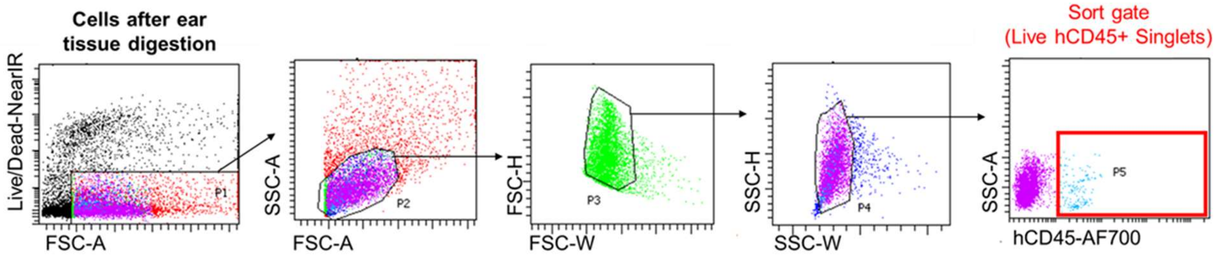

Cells after ear tissue digestion
Live/Dead-NearIR
FSC-A
P1
SSC-A
FSC-A
P2
FSC-H
FSC-W
P3
SSC-H
SSC-W
P4
Sort gate
(Live hCD45+ Singlets)
SSC-A
hCD45-AF700
P5

e.

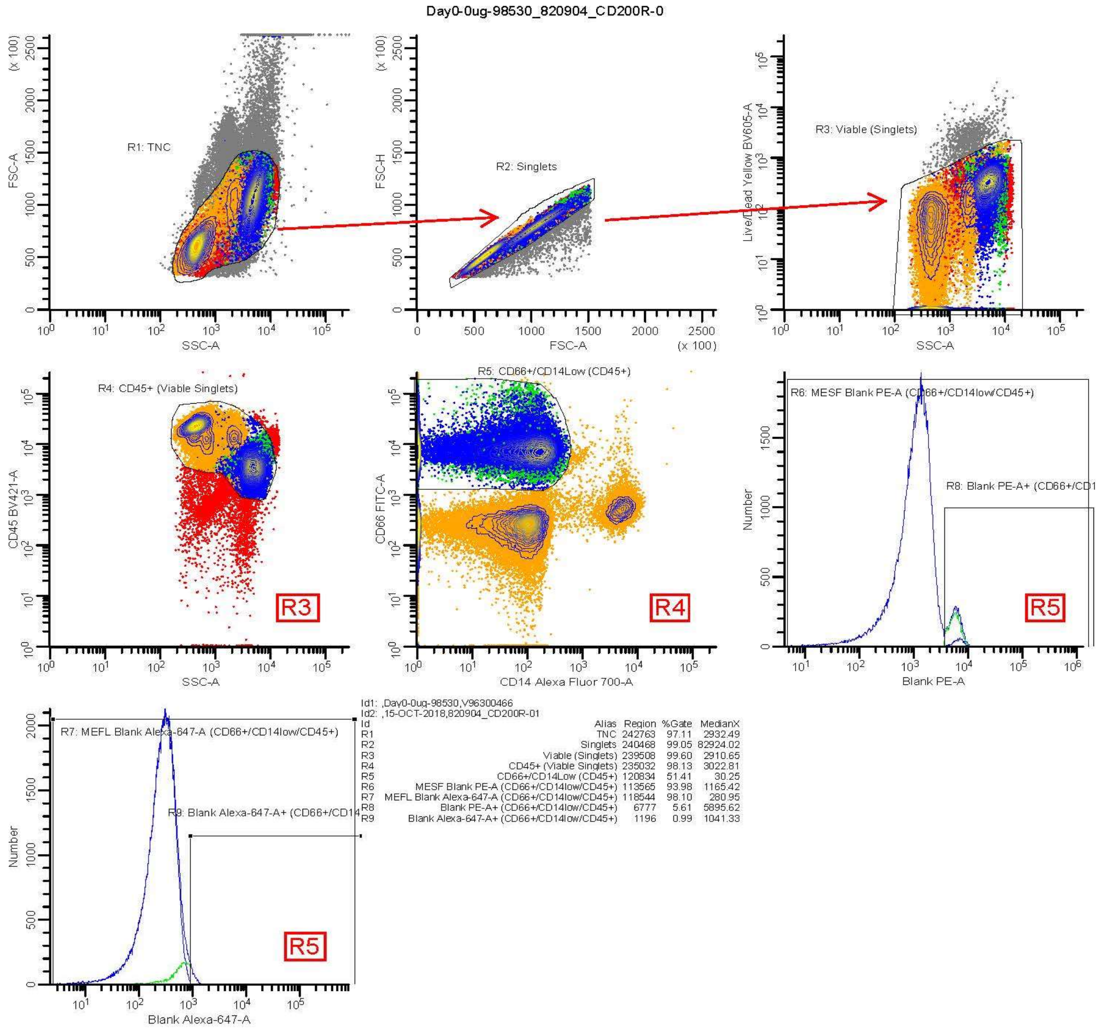

Id1: ,Day0-0ug-98530,V96300466
Id2: ,15-OCT-2018,820904_CD200R-01

Id	Alias	Region	%Gate	MedianX
R1	TNC	242763	97.11	2932.49
R2	Singlets	240468	99.05	82924.02
R3	Viable (Singlets)	239508	99.60	2910.65
R4	CD45+ (Viable Singlets)	235032	98.13	3022.81
R5	CD66+/CD14Low (CD45+)	120834	51.41	30.25
R6	MESF Blank PE-A (CD66+/CD14low/CD45+)	113565	93.98	1165.42
R7	MEFL Blank Alexa-647-A (CD66+/CD14low/CD45+)	118544	98.10	280.95
R8	Blank PE-A+ (CD66+/CD14low/CD45+)	6777	5.61	5895.62
R9	Blank Alexa-647-A+ (CD66+/CD14low/CD45+)	1196	0.99	1041.33

f.

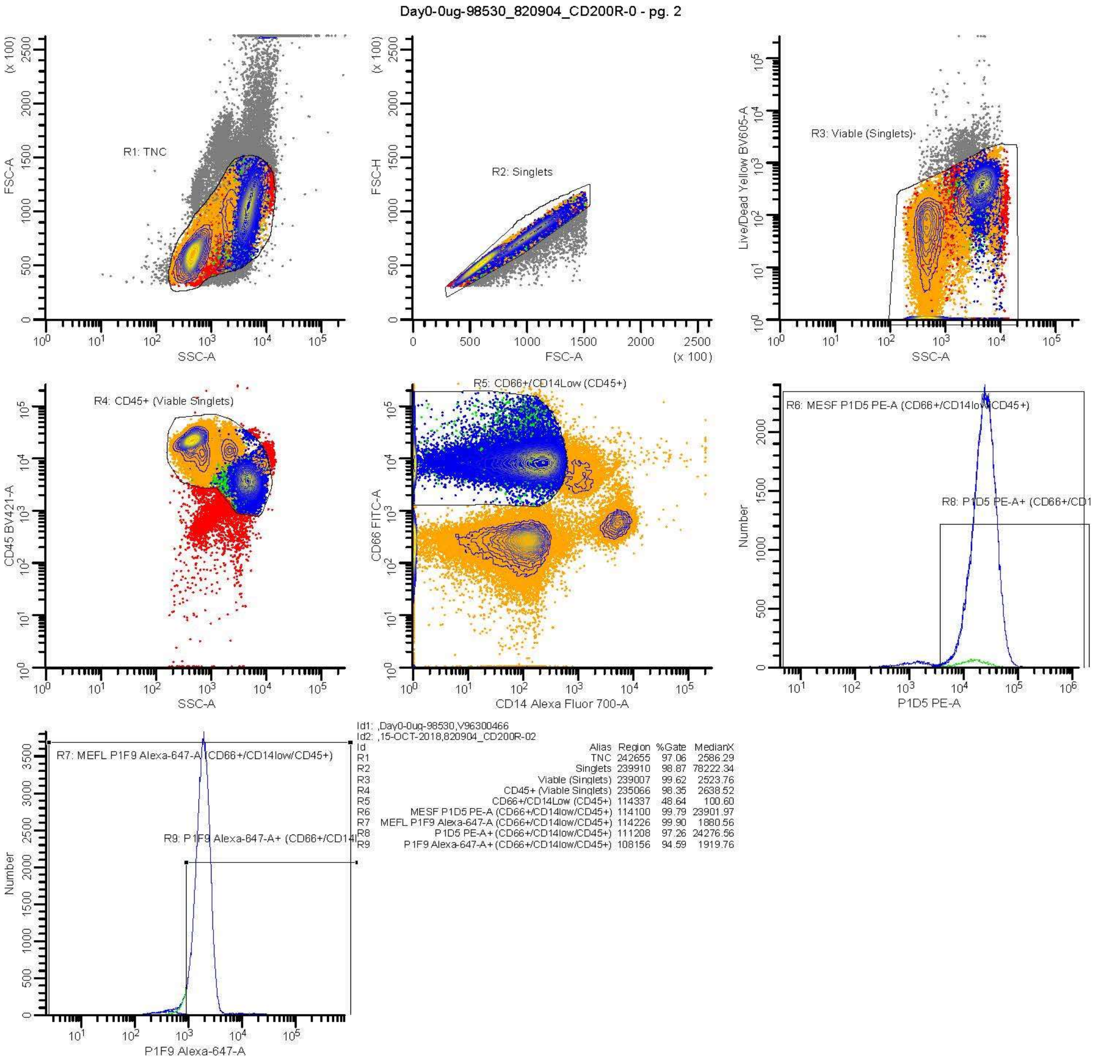

Id	Alias	Region	%Gate	MedianX
R1	TNC	242655	97.06	2586.29
R2	Singlets	239910	98.87	78222.34
R3	Viable (Singlets)	239007	99.62	2523.76
R4	CD45+ (Viable Singlets)	235066	98.35	2638.52
R5	CD66+/CD14Low (CD45+)	114337	48.64	100.60
R6	MESF P1D5 PE-A (CD66+/CD14low/CD45+)	114100	99.79	23901.97
R7	MEFL P1F9 Alexa-647-A (CD66+/CD14low/CD45+)	114226	99.90	1880.56
R8	P1D5 PE-A+ (CD66+/CD14low/CD45+)	111208	97.26	24276.56
R9	P1F9 Alexa-647-A+ (CD66+/CD14low/CD45+)	108156	94.59	1919.76

Supplementary Fig. 5 | Gating strategies for flow cytometry figures. a, Fig. 1b: ucenprubart staining on CHO cells expressing human or cynomolgus CD200R, cynomolgus CD200RLa, or naïve CHO cells; plots shown are for 500ug/ml ucenprubart staining conditions. **b**, Fig. 1c: identification of cells stained with labeled CD200Fc with and without ucenprubart pre-incubation. **c** Fig. 2c: identifying numbers of dividing CD4 cells in response to SEB stimulation with and without ucenprubart treatment. **d**, Fig. 3: sorting human CD45+ lymphocytes from mouse ear tissue.

e, gating strategy for ucenprubart receptor occupancy on neutrophils (CD66+/DC14low/CD45+) exemplary from validation report: tube 1 for FM0 (Fluorescence minus one, gate R9), **Tube 1: 820904_CD200R-01**

1: Total nucleated cells are gated in (R1).
2: Singlets are gated in (R2).
3: Viable (Singlets) are gated in (R3).
4: CD45+ (Viable Singlets) are gated in (R4).
5: CD66+/CD14Low (CD45+) cells are gated in (R5).
6: The (R8) percent positive gate is set on the blank to observe the percent positive and MESF (Molecules of Equivalent Soluble Fluorochrome) of P1D5 PE + (non-competing CD200R antibody to detect total CD200R) (CD66+/CD14low/CD45+) in Tube 2.
7: The (R9) percent positive gate is set on the blank to observe the percent positive and MEFL(Molecules of Equivalent Flurescein) of P1F9 AF647+ (CD66+/CD14low/CD45+) in Tube 2.

f, gating strategy for ucenprubart receptor occupancy on neutrophils (gate R9, CD66+/DC14low/CD45+), tube 2 5ug/ml ucenprubart spiked into test tube **Tube 2: 820904_CD200R-02**

1: Total nucleated cells are gated in (R1).
2: Singlets are gated in (R2).
3: Viable (Singlets) are gated in (R3).
4: CD45+ (Viable Singlets) are gated in (R4).
5: CD66+/CD14Low (CD45+) cells are gated in (R5).
6: Observe the percent positive and intensity of P1D5 PE + (non-competing CD200R antibody to detect total CD200R) (CD66+/CD14low/CD45+) in gate (R8). No adjustment needed on the MESF gate (R6).
7: Observe the percent positive and intensity of ucenprubart (P1F9) AF647+ (CD66+/CD14low/CD45+) in gate (R9).

CHO, Chinese hamster ovary; cy, cynomolgus; hu, human;

Supplemental Fig 6: Correlation of CD3+ and GATA3+ cells in the Dermis

The analysis of data from percent positive CD3+ and percent positive GATA3+ immune cells, relative to all dermal cells as determined by immunohistochemistry across all available matching samples (n=57), revealed a significant correlation (Pearson r = 0.7546, $p < 0.0001$).

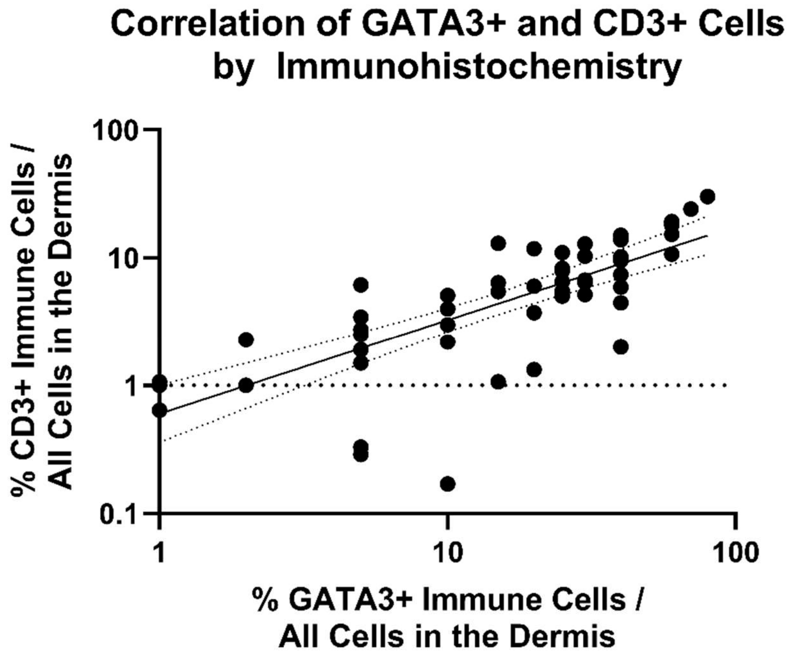

Pearson r	
r	0.7546
95% confidence interval	0.6150 to 0.8484
R squared	0.5694
P value	
P (two-tailed)	<0.0001
P value summary	****
Significant? (alpha = 0.05)	Yes

Supplementary Table 1 | Affinity of ucenprubart to human, cynomolgus monkey, and mouse CD200R as measured by surface plasmon resonance

CD200 receptor	Average K_D	Standard deviation
Human	5.6 nM	1.2 nM
Cynomolgus monkey	2.3 nM	0.1 nM
Cynomolgus monkey activating	2.5 µM	0.4 µM
Mouse	5.1 µM	0.2 µM

K_D, dissociation constant.

Supplementary Table 2 | In vitro binding parameters of ucenprubart to human FcγR extracellular domains measured using surface plasmon resonance at 25 °C

Sample	Human ligand	Average K_D	Standard deviation
Hu IgG1	FcγRI	64.3 pM	10.3
Hu IgG4PAA	FcγRI	>200 nM	—
Hu IgG4P	FcγRI	472 pM	28.9
Ucenprubart	FcγRI	474 pM	24.3
Hu IgG1	FcγRIIa-131H	0.6 µM	0.1
Hu IgG4PAA	FcγRIIa-131H	>10 µM	—
Hu IgG4P	FcγRIIa-131H	4.1 µM	0.4
Ucenprubart	FcγRIIa-131H	4.4 µM	0.4
Hu IgG1	FcγRIIa-131R	0.8 µM	0.1
Hu IgG4PAA	FcγRIIa-131R	>10 µM	—
Hu IgG4P	FcγRIIa-131R	1.6 µM	0.2
Ucenprubart	FcγRIIa-131R	1.8 µM	0.2
Hu IgG1	FcγRIIb	4.4 µM	0.4
Hu IgG4PAA	FcγRIIb	>10 µM	—
Hu IgG4P	FcγRIIb	1.6 µM	0.1
Ucenprubart	FcγRIIb	1.9 µM	0.2
Hu IgG1	FcγRIIIa-158V	0.2 µM	0.0
Hu IgG4PAA	FcγRIIIa-158V	8.9 µM	0.8
Hu IgG4P	FcγRIIIa-158V	3.1 µM	0.7
Ucenprubart	FcγRIIIa-158V	5.0 µM	1.3
Hu IgG1	FcγRIIIa-158F	1.4 µM	0.2
Hu IgG4PAA	FcγRIIIa-158F	>10 µM	—
Hu IgG4P	FcγRIIIa-158F	10 µM	0.5
Ucenprubart	FcγRIIIa-158F	>10 µM	—

Note: n = 3× assayed.

Hu IgG4P: Monoclonal isotype antibody that contains 1 mutation in the hinge region, S228P, to prevent antibody arm exchange. Hu IgG4PAA: Monoclonal isotype antibody that contains 1 mutation in the hinge region, S228P, to prevent antibody arm exchange, which also contains 3 other mutations within the CH2 domain, F234A and L235A, to further reduce the already weak binding of IgG4 to FcγRs.

FcγR, crystallizable fragment gamma receptor; Hu, human; IgG, immunoglobulin G; K_D, dissociation constant.

Supplementary Table 3 | Geometric mean values of C_{max} and AUC of ucenprubart

Dosing regimen and participant population	C_{max} (µg/mL)	$AUC_{0-\infty}$ (µg/mL·h)
1 mg IV single dose in healthy participants	0.330	14.8
5 mg IV single dose in healthy participants	1.81	137
25 mg IV single dose in healthy participants	14.0	1370
100 mg IV single dose in healthy participants	63.2	10,900
100 mg SC single dose in healthy participants	11.9	5970
300 mg IV single dose in healthy participants	143	36,500
1000 mg IV single dose in healthy participants	408	119,000
200 mg IV on day 1 in healthy participants	84.6	13,400*
200 mg IV on day 15 in healthy participants	110	19,400*
500 mg IV on day 1 in participants with AD	151	20,100*
500 mg IV on day 85 in participants with AD	244	56,600*

*$AUC_{0-\tau}$.

AD, atopic dermatitis; AUC, area under the concentration–time curve; $AUC_{0-\infty}$, area under the concentration–time curve from time 0 to infinity; $AUC_{0-\tau}$, area under the concentration–time curve from time 0 to the end of the dosing interval; C_{max}, maximum observed drug concentration; IV, intravenous; SC, subcutaneous.

Supplementary Table 4 | Patient demographics and baseline characteristics in Part C

Parameter	Placebo IV (N = 12)	Ucenprubart 500 mg IV (N = 28)
Age, mean (SD), years	36.8 (15.0)	42.6 (15.8)
Female sex, n (%)	6 (50.0)	17 (60.7)
Country of enrollment, n (%)		
Bulgaria	1 (8.3)	1 (3.6)
Puerto Rico	0	3 (10.7)
United States	11 (91.7)	24 (85.7)
Weight, mean (SD), kg	80.7 (13.0)	78.3 (20.8)
BMI, mean (SD), kg/m^2	27.7 (4.6)	28.3 (6.3)
vIGA-AD, mean (SD)	3.1 (0.3)	3.2 (0.4)
EASI total score, mean (SD)	20.5 (9.8)	20.2 (11.9)
SCORAD total score, mean (SD)	61.8 (13.1)	58.8 (12.0)

BMI, body mass index; EASI, Eczema Area and Severity Index; IV, intravenously; SCORAD, SCORing Atopic Dermatitis; SD, standard deviation; vIGA-AD, validated Investigator Global Assessment for Atopic Dermatitis.

Supplementary Note 1 | Summary of pharmacokinetic results

Following a single intravenous (IV) infusion of ucenprubart to healthy participants, the geometric mean apparent terminal elimination half-life ($t_{1/2}$) of ucenprubart was 59.3 to 355 hours across the 1- to 1000-mg dose range, which appeared to increase with increasing dose.

Following a single subcutaneous infusion of ucenprubart 100 mg to healthy participants, the geometric mean $t_{1/2}$ of ucenprubart was approximately 180 hours, slightly shorter than the geometric mean for the 100-mg IV dose (215 hours).

Following repeated biweekly IV infusions of ucenprubart 200 mg to healthy participants, the geometric mean $t_{1/2}$ of ucenprubart was 219 hours on day 1 and 324 hours on day 15.

Following repeated biweekly IV infusions of ucenprubart 500 mg to participants with atopic dermatitis, the geometric mean $t_{1/2}$ of ucenprubart was 188 hours on day 1 and 340 hours on day 85.

The pharmacokinetics of ucenprubart showed a greater than dose proportional increase in the maximum observed drug concentration and the area under the concentration–time curve from time 0 to infinity across the 1- to 1000-mg dose range (Supplementary Table 3).

Supplementary Note 2 | Additional comment from authors

When preparing the submission of our analysis code for the single cell portion of the manuscript, we noticed inconsistencies between the now commonly used Seurat version 5 software and the previously used version 4 packages for data analysis (V4.1, V4.2). This known issue (https://github.com/satijalab/seurat/issues/6586) affected differential gene expression results and log2 fold changes in Fig. 3 and subsequent down-stream/pathway analysis. To address this issue, we re-analyzed the dataset using the current Seurat version 5. This also increases reproducibility of our single cell analysis pipeline since the older version 4 code is incompatible with the current Seurat version 5. While the main findings are not altered, we noticed slight changes between previously reported numbers of cells and differentially expressed genes. We have revised Fig. 3 and the subsequent figures in the supplement to report findings consistent with the latest Seurat version 5 analysis, and updated the numbers of genes in the text. We also added a KEGG pathway mapping for differentially expressed genes in macrophages (Fig. 3f) and a list of these genes to address the remaining comment by reviewer 3 to improve readability.

Protocol J1B-MC-FRCC(e)
Phase 1, Multicenter, Randomized, Placebo-Controlled, Triple-Blind, Single-Ascending Dose and Repeat-Dose Trial in Healthy Participants and Participants with Atopic Dermatitis

Confidential Information

The information contained in this protocol is confidential and is intended for the use of clinical investigators. It is the property of Eli Lilly and Company or its subsidiaries and should not be copied by or distributed to persons not involved in the clinical investigation of LY3454738, unless such persons are bound by a confidentiality agreement with Eli Lilly and Company or its subsidiaries.

Note to Regulatory Authorities: This document may contain protected personal data and/or commercially confidential information exempt from public disclosure. Eli Lilly and Company requests consultation regarding release/redaction prior to any public release. In the United States, this document is subject to Freedom of Information Act (FOIA) Exemption 4 and may not be reproduced or otherwise disseminated without the written approval of Eli Lilly and Company or its subsidiaries.

LY3454738

Eli Lilly and Company
Indianapolis, Indiana USA 46285

Clinical Pharmacology Protocol Electronically Signed and Approved by Lilly:
01 October 2018
Amendment (a) Electronically Signed and Approved by Lilly: 29 January 2019
Amendment (b) Electronically Signed and Approved by Lilly: 24 April 2019
Amendment (c) Electronically Signed and Approved by Lilly: 01 July 2019
Amendment (d) Electronically Signed and Approved by Lilly: 04 May 2020
Amendment (e) Electronically Signed and Approved by Lilly on date provided below.

Approval Date: 19-Apr-2021 GMT

Table of Contents

Phase 1, Multicenter, Randomized, Placebo-Controlled, Triple-Blind, Single-Ascending Dose and Repeat-Dose Trial in Healthy Participants and Participants with Atopic Dermatitis

Section **Page**

Protocol J1B-MC-FRCC(e) Phase 1, Multicenter, Randomized, Placebo-Controlled, Triple-Blind, Single-Ascending Dose and Repeat-Dose Trial in Healthy Participants and Participants with Atopic Dermatitis....1

Table of Contents....2

1. Protocol Synopsis....9

2. Schedule of Activities....12

3. Introduction....26

3.1. Study Rationale....26

3.2. Background....26

3.3. Benefit/Risk Assessment....28

4. Objectives and Endpoints....30

5. Study Design....32

5.1. Overall Design....32

5.1.1. Part A (SAD Design in Healthy Participants)....34

5.1.2. Part B (Repeat-Dose Design in Healthy Participants)....35

5.1.3. Part C (Repeat-Dose Design in Participants with AD)....35

5.2. Number of Participants....35

5.2.1. Part A and Part B (Healthy Participants)....35

5.2.2. Part C (Participants with AD)....36

5.3. End of Study Definition....36

5.4. Scientific Rationale for Study Design....36

5.5. Justification for Dose....38

6. Study Population....41

6.1. Inclusion Criteria for All Participants....41

6.1.1. Additional Inclusion Criteria for Part C (Participants with AD)....43

6.2. Exclusion Criteria for All Participants....44

6.2.1. Additional Exclusion Criterion for Part A (Healthy Participants)....46

6.2.2. Additional Exclusion Criteria for Part C (Participants with AD) 46
6.2.3. Rationale for Exclusion of Certain Study Candidates 48
6.3. Lifestyle and/or Dietary Requirements 48
6.3.1. Meals and Dietary Restrictions 48
6.3.2. Caffeine, Alcohol, and Tobacco 48
6.3.3. Activity 48
6.3.4. Contraception 48
6.4. Screen Failures 49
7. Treatment 50
7.1. Treatment Administered 50
7.1.1. Packaging and Labeling 51
7.2. Method of Treatment Assignment 52
7.2.1. Selection and Timing of Doses 52
7.3. Blinding 52
7.4. Dose Modification 53
7.4.1. Dose Escalation (Part A) 53
7.4.2. Dose Decision (Part B) 54
7.4.3. Dose Decision (Part C) 54
7.4.4. Special Treatment Considerations 55
7.4.4.1. Premedication for Infusions 55
7.4.4.2. Management of Infusion Reactions 55
7.5. Preparation/Handling/Storage/Accountability 56
7.6. Treatment Compliance 56
7.7. Concomitant Therapy 56
7.8. Treatment after the End of the Study 57
8. Discontinuation Criteria 58
8.1. Discontinuation from Study Treatment 58
8.1.1. Part A (SAD Design in Healthy Participants) 58
8.1.2. Part B and Part C (Repeat-Dose in Healthy Participants and Participants with AD) 58
8.1.3. Discontinuation of Inadvertently Enrolled Participants 59
8.2. Discontinuation from the Study 59
8.3. Participants Lost to Follow-up 60
9. Study Assessments and Procedures 61
9.1. Efficacy Assessments 61
9.2. Adverse Events 62
9.2.1. Serious Adverse Events 63

9.2.1.1. Suspected Unexpected Serious Adverse Reactions....64
9.2.2. Complaint Handling....64
9.3. Treatment of Overdose....64
9.4. Safety....64
9.4.1. Laboratory Tests....64
9.4.2. Vital Signs....64
9.4.3. Electrocardiograms....65
9.4.4. Physical Examination....65
9.4.5. Injection Site Assessments....66
9.4.6. Safety Monitoring....66
9.4.6.1. Hepatic Safety....66
9.5. Pharmacokinetics....67
9.5.1. Bioanalysis....67
9.6. Pharmacodynamics....68
9.6.1. Receptor Occupancy....68
9.6.2. Immunogenicity Assessments....68
9.7. Genetics....68
9.8. Biomarkers....69
9.8.1. Skin Biopsies for Participants with AD....70
9.9. Health Economics....70
10. Statistical Considerations and Data Analysis....71
10.1. Sample Size Determination....71
10.2. Populations for Analyses....71
10.2.1. Study Participant Disposition....71
10.2.2. Study Participant Characteristics....71
10.3. Statistical Analyses....71
10.3.1. Safety Analyses....72
10.3.1.1. Clinical Evaluation of Safety....72
10.3.1.2. Statistical Evaluation of Safety....73
10.3.2. Efficacy Analyses....73
10.3.2.1. Primary Analyses....73
10.3.2.2. Secondary Analyses....73
10.3.2.3. Exploratory Analyses....73
10.3.3. Pharmacokinetic Analyses....74
10.3.3.1. Pharmacokinetic Parameter Estimation....74
10.3.3.2. Pharmacokinetic Statistical Inference....74
10.3.4. Pharmacodynamic Analyses....74
10.3.4.1. Pharmacodynamic Parameter Estimation....74

10.3.5. Pharmacokinetic/Pharmacodynamic Analyses 74
10.3.6. Evaluation of Immunogenicity 74
10.3.7. Data Review during the Study 75
10.3.8. Interim Analyses 75
10.3.9. Data Monitoring Committee (DMC) 76
11. References 77

List of Tables

Table **Page**

Table FRCC.1. Objectives and Endpoints 30
Table FRCC.2. Summary of Participant Cohorts 33
Table FRCC.3. Planned Doses, Projected Human PK Exposure, and Projected RO.........40
Table FRCC.4. Treatments Administered..51

List of Figures

Figure **Page**

Figure FRCC.1. J1B-MC-FRCC study design. 34

List of Appendices

Appendix		Page
Appendix 1.	Abbreviations and Definitions	79
Appendix 2.	Clinical Laboratory Tests	86
Appendix 3.	Study Governance, Regulatory and Ethical Considerations	87
Appendix 4.	Hepatic Monitoring Tests for Treatment-Emergent Abnormality	90
Appendix 5.	Blood Sampling Summary	91
Appendix 6.	Validated Investigator Global Assessment Scale for Atopic Dermatitis (vIGA-AD)	93
Appendix 7.	Protocol Amendment J1B-MC-FRCC(d) Summary Phase 1, Multicenter, Randomized, Placebo-Controlled, Triple-Blind, Single-Ascending Dose and Repeat-Dose Trial in Healthy Participants and Participants with Atopic Dermatitis	94

1. Protocol Synopsis

Title of Study:

Phase 1, Multicenter, Randomized, Placebo-Controlled, Triple-Blind, Single-Ascending Dose and Repeat-Dose Trial in Healthy Participants and Participants with Atopic Dermatitis

Rationale:

This study is a first-in-human trial designed to evaluate safety, tolerability, pharmacokinetics (PK) and target engagement of LY3454738 in healthy participants and participants with atopic dermatitis (AD), with additional assessments of clinical pharmacodynamics and efficacy in participants with AD. The inclusion of healthy Japanese participants in this study will facilitate the inclusion of Japanese participants in subsequent clinical trials. The trial will be triple-blind and placebo-controlled in order to avoid bias in the collection and evaluation of data and to enable characterization of adverse events (AEs).

The study is designed in 3 parts, with staggered initiation. Part A will assess LY3454738 in single-ascending doses across a planned dose range to encompass the predicted clinical efficacious exposure. Part B will assess repeat dosing of LY3454738 at a dose level expected to have submaximal exposure in order to assess any acute safety/tolerability events associated with a second exposure. A portion of the participants in Parts A and B will be Japanese. Parts A and B will be conducted in a confined Clinical Research Unit (CRU) in healthy participants to maximize appropriate safety oversight and to assess the safety, tolerability, PK and target engagement of LY3454738 in a setting with minimal physiologic variability that disease states could introduce. Part C will assess repeat dosing of LY3454738 at a single dose level predicted to give efficacious exposures in participants with AD, in order to evaluate its impact on clinical pharmacodynamics and efficacy. This part of the study will be conducted in an ambulatory setting, given that the steady state exposure achieved over the dosing interval in Part C will not exceed the exposure achieved and previously assessed for safety/tolerability in Part A. Dosing will primarily be intravenous (IV) to maximize achieving the predicted efficacious exposures at this early stage of development. A single subcutaneous (SC) cohort will be included in Part A to provide an estimate of relative bioavailability, given that SC dosing will be the preferred route to treat target populations. The planned dose levels, frequency, and distribution of Japanese and non-Japanese participants may be modified based on emerging data collected during the study.

Atopic dermatitis is primarily driven by a Th2-mediated immune response magnified by pruritic inflammatory skin. The target for LY3454738, the CD200 receptor (CD200R), is expressed on many of the known key cell types important in AD pathogenesis. Further, CD200R expression is increased in lesional skin of patients with AD. CD200R ligand or agonist antibodies (including LY3454738) inhibit immune cell activation and CD200R agonist antibodies have also demonstrated robust in vivo efficacy in preclinical skin inflammation models. Data generated from Part C will guide the future development of the compound in AD.

Objective(s)/Endpoints:

Objectives	Endpoints
**Primary**	
• To assess the safety and tolerability of LY3454738 after single IV and SC dosing in healthy participants and after multiple IV dosing in healthy participants and participants with AD	Incidence of adverse events (AEs), treatment-emergent adverse events (TEAEs), and serious adverse events (SAEs)
• To evaluate the efficacy of LY3454738 after multiple IV dosing in participants with AD at Week 12	Proportion of participants achieving a Validated Investigator's Global Assessment for Atopic Dermatitis (vIGA-AD) score of 0 or 1 with a ≥ 2-point improvement from baseline at Week 12.
**Secondary**	
• To characterize the pharmacokinetics (PK) of LY3454738 following single (IV and SC) and multiple (IV) dosing administration in healthy participants and after multiple IV dosing in participants with AD	maximum observed drug concentration (C_{max}) and area under the concentration versus time curve (AUC)
• To evaluate the efficacy of LY3454738 over time after multiple IV dosing in participants with AD	At Week 1 through Week 12 and/or early discontinuation, the proportion of participants achieving:<br>• vIGA-AD of 0 or 1 with a ≥2-point improvement from baseline,<br>• 50%, 75%, and 90% reduction from baseline in Eczema Area and Severity Index (EASI), and 50%, 75%, and 90% improvement from baseline in SCORing Atopic Dermatitis (SCORAD)<br><br>Mean change from baseline in EASI and SCORAD at Week 1 through Week 12 and/or early discontinuation

Summary of Study Design:

Study J1B-MC-FRCC is a Phase 1, multicenter, randomized, placebo-controlled, triple-blind study with 3 study parts: Part A has a single-ascending dose (SAD) design in healthy participants, Part B has a repeat-dose design in healthy participants, and Part C has a repeat-dose design in participants with AD.

Treatment Arms and Planned Duration for an Individual Participant:

In Part A, 6 IV dose cohorts (1, 5, 25, 100, 300, and 1000 mg) and 1 SC dose cohort (100 mg) are planned and study drug will be administered as a single dose on Day 1, followed by a 12-week follow-up period. In Part B, study drug will be administered on Days 1 and 15, followed by a 12-week follow-up period. In Part C, study drug will be administered every 2 weeks for 12 weeks, followed by a 12-week follow-up period. Individual participants will enroll to a single part of the study only. In Part A, participants will enroll to only 1 cohort. In Part A, sentinel

dosing will be used in Cohorts 1 and 2 for 2 participants (1 placebo and 1 LY3454738). Each IV cohort in all parts of the study will include treatment-matched placebo via the same route of administration as LY3454738.

Number of Participants:

Enrollment for Parts A and B will occur to enable completion of approximately 62 participants (54 participants and 8 participants in Part A and Part B, respectively) in these parts of trial (ie, completion of all scheduled procedures up to and including Day 85 in Part A and Day 99 in Part B). Enrollment for Part C will occur to enable completion of approximately 30 participants in this part of the trial (ie, completion of all scheduled procedures up to and including Day 169).

Statistical Analysis:

All investigational product (IP) and protocol procedure AEs will be listed and, if the frequency of events allows, safety data will be summarized using descriptive methodology. The incidence of symptoms for each treatment will be presented by severity and by association with IP as perceived by the investigator. The number of participants who experience a TEAE and/or SAE (all causalities and related to study drug) will be summarized by study treatment. All TEAEs will be summarized by system organ class and by decreasing frequency within system organ class. Infusion- and injection-site reactions will be summarized by maximum Common Terminology Criteria for Adverse Events (CTCAE) grade. (Please note that CTCAE will only be used for infusion-related reactions and injection/infusion site-related AEs).

For Part C, Fisher's exact test will be used for treatment comparisons of discrete efficacy variables. The percentages and difference in percentages will be reported. Continuous efficacy variables will be analyzed by an analysis of covariance (ANCOVA) with treatment and baseline value in the model. Descriptive statistics will be reported.

2. Schedule of Activities

Study Schedule for Protocol J1B-MC-FRCC (Part A: Single-Ascending Dose Design in Healthy Participants)

Procedures/Assessments	Screening	Baseline/Dosing		Postdose Follow-up										
Visit Number	V1	V2		V3	V4	V5[a]	V6	V7	V8	V9	V10	V11	V12	ED
Study Day	-D28 to -D2	1	2	5 ± 1 d	8 ± 1 d	11 ± 1 d	15 ± 2 d	22 ± 2 d	29 ± 3 d	43 ± 3 d	57 ± 3 d	71 ± 3 d	85 ±3 d	
Week(s)		0			1		2	3	4	6	8	10	12	
Informed consent	X													
Review/confirm I/E criteria	X	X[b]												
Admission to CRU[c]		X												
Discharge from CRU[c]			X											
Clinical Assessments														
Complete medical history	X													
Review preexisting conditions/AEs	X	X[b]	X	X	X	X	X	X	X	X	X	X	X	X
Concomitant medications	X	X[b]	X	X	X	X	X	X	X	X	X	X	X	X
Substance use (alcohol, tobacco use)	X													
Physical examination[d]	X	X											X	X
Weight	X												X	X
Height	X													
Vital signs (pulse rate, blood pressure, and temperature)[e,f]	X	X[b]	X	X	X	X	X	X	X	X	X	X	X	X
ECGs[f,g]	X	X	X		X		X		X		X		X	X
Chest x-ray[h] (posterior-anterior view)	X													

**Procedures/Assessments**	**Screening**	**Baseline/Dosing**		**Postdose Follow-up**										
**Visit Number**	**V1**	**V2**		**V3**	**V4**	**V5[a]**	**V6**	**V7**	**V8**	**V9**	**V10**	**V11**	**V12**	**ED**
**Study Day**	**-D28 to -D2**	**1**	**2**	**5 ± 1 d**	**8 ± 1 d**	**11 ± 1 d**	**15 ± 2 d**	**22 ± 2 d**	**29 ± 3 d**	**43 ± 3 d**	**57 ± 3 d**	**71 ± 3 d**	**85 ±3 d**	
**Week(s)**		**0**			**1**		**2**	**3**	**4**	**6**	**8**	**10**	**12**	
QuantiFERON®-TB Gold test or TST[i]	X													
IP administration (IV or SC)		X												
Laboratory Assessments[j]														
HIV/Hepatitis B (surface antigen and core antibody)/C Testing	X													
Serum immunoglobulins (IgG, IgM, IgA)	X				X		X						X	X
Urinary drug screen	X													
FSH[k]	X													
Pregnancy test[k]	X	X							X		X		X	X
Clinical chemistry	X	X	X		X		X	X	X	X	X	X	X	X
Hematology	X	X	X	X	X		X	X	X	X	X	X	X	X
Urinalysis	X	X		X			X		X	X			X	X
Pharmacogenetics		X[l]												
Immunogenicity[m]		X[l]					X		X				X	X
Blood samples for flow cytometry (safety)		X[l]			X		X		X		X	X		

Procedures/Assessments	Screening	Baseline/Dosing		Postdose Follow-up										
**Visit Number**	**V1**	**V2**		**V3**	**V4**	**V5[a]**	**V6**	**V7**	**V8**	**V9**	**V10**	**V11**	**V12**	**ED**
**Study Day**	**-D28 to -D2**	**1**	**2**	**5 ± 1 d**	**8 ± 1 d**	**11 ± 1 d**	**15 ± 2 d**	**22 ± 2 d**	**29 ± 3 d**	**43 ± 3 d**	**57 ± 3 d**	**71 ± 3 d**	**85 ±3 d**	
**Week(s)**		**0**			**1**		**2**	**3**	**4**	**6**	**8**	**10**	**12**	
LY3454738 concentration (PK)[j]	Schedule depends on IV or SC administration													
IV cohorts[f,m,n]		X	X		X		X		X		X		X	X
SC cohort[f,m,o]		X	X	X	X	X	X	X	X		X		X	X
Receptor occupancy[j]	Schedule depends on IV or SC administration													
IV cohorts[p]		X	X		X				X				X	X
SC cohort[q]		X	X	X	X	X		X	X				X	X
Exploratory serum samples[j]	Schedule depends on IV or SC administration													
IV cohorts[r]		X	X		X				X				X	X
SC cohort[s]		X	X	X	X	X		X	X				X	X

Abbreviations: AE = adverse event; CRU = clinical research unit; D = study day; d = days; ECG = electrocardiogram; ED = early discontinuation; FSH = follicle-stimulating hormone; HIV = human immunodeficiency virus; I/E = inclusion/exclusion; Ig = immunoglobulin; IP = investigational product; IV = intravenous; PK = pharmacokinetic; SC = subcutaneous; TB = tuberculosis; TST = tuberculin skin test; V = visit.

[a] Required for participants receiving SC dosing only.

[b] At the discretion of the clinical site, the baseline measurement for this assessment can be collected at any time prior to dosing on the day of dosing.

[c] Admission to the CRU may occur on the day prior to dosing or during morning of the day of dosing at the discretion of the CRU, and discharge from the CRU should occur following completion of the Day 2 assessments.

[d] Symptom-directed physical examinations may be conducted at any time during the study, as deemed necessary by the investigator.

[e] Vital signs should be taken following an approximate 5-minute rest in supine position. Temperature measurement is required only at screening and baseline.

[f] On days with concurrent ECG, vital sign, and/or PK sampling, these measurements should occur at approximately the same time. ECG recording and vital sign measurements should occur prior to the blood draw. Participants must be supine for approximately 5 to 10 minutes before ECG collection and remain supine but awake during ECG collection.

[g] Times are referenced to end of dosing. ECGs will be obtained on Day 1 (predose [any time prior to dosing on the day of dosing, but within 90 minutes prior to the predose blood draw], end of infusion, and 6 h after the end of infusion for the IV cohort and pre-dose only for the SC cohort), Day 2 (24 h after the end of dosing for both the IV and SC cohorts) and at any time on other specified visits (but prior to any blood draws at same visit).

[h] A posterior–anterior chest x-ray will be performed at screening unless one has been performed within the past 6 months and the x-ray and reports are available.

[i] The follow-up TST reading should occur 2 to 3 days after V1.

[j] If more than 1 laboratory blood draw is scheduled at the same time, these blood draws should be obtained in the following order: (1) safety laboratory tests (2) PK samples, (3) receptor occupancy samples, and (4) exploratory samples.

[k] A serum pregnancy test will be conducted at screening only. Urine pregnancy test will be used at all other time points. For women who are considered to be not of childbearing potential, FSH should be drawn to confirm status as defined in Inclusion Criterion [1b] and to be considered exempt for further pregnancy tests during the study.

[l] This activity should be completed before IP administration (predose).

[m] In the event of drug hypersensitivity reactions (immediate or nonimmediate), up to 3 additional samples will be collected each for PK and immunogenicity at the following time points: as close to the onset of the reaction event as possible, at the resolution of the event, and approximately 30 days following the event.

[n] Time points for IV PK sampling with time windows in parentheses:

- Day 1: predose (-10 minutes), end of infusion (±5 minutes), and 30 minutes (±5 minutes), 1 hour (±5 minutes), 2 hours (±10 minutes), 6 hours (±10 minutes), and 12 hours (±15 minutes) after the end of IV infusion
- Day 2: 24 hours (±15 minutes) after the end of IV infusion
- Days 8, 15, 29, 57, 85, and
- early discontinuation if necessary.

The actual date and exact 24-hour clock time of sample collection should be recorded. Samples are requested to be taken at the specified time; however, aberrations to the specified sampling times will not be considered protocol deviations as long as the samples are taken and the actual sampling time is recorded. It is essential that the actual times of doses and samples are recorded accurately on the appropriate forms.

[o] Time points for SC PK sampling with time windows in parentheses:

- Day 1: predose (-10 minutes), and 6 hours (±10 minutes) and 12 hours (±15 minutes) after SC injection
- Day 2: 24 hours (±15 minutes) after SC injection
- Days 5 (±3 hours), 8, 11, 15, 22, 29, 57, 85, and
- early discontinuation if necessary.

The actual date and exact 24-hour clock time of sample collection should be recorded. Samples are requested to be taken at the specified time; however, aberrations to the specified sampling times will not be considered protocol deviations as long as the samples are taken and the actual sampling time is recorded. It is essential that the actual times of doses and samples are recorded accurately on the appropriate forms.

[p] Time points for receptor occupancy sampling for IV dosing cohorts with time windows in parentheses:

- Day 1: predose (-10 minutes), end of infusion (±5 minutes), and 30 minutes (±5 minutes), 1 hour (±5 minutes), 2 hours (±10 minutes), and 4 hours (±10 minutes) after the end of IV infusion
- Day 2: 24 hours (±15 minutes) after the end of IV infusion

- Days 8, 29, 85, and
- early discontinuation if necessary.

[q] Time points for receptor occupancy sampling for SC dosing cohort with time windows in parentheses:

- Day 1: predose (-10 minutes), and 4 hours (±10 minutes), and 12 hours (±15 minutes) after SC injection
- Day 2: 24 hours (±15 minutes) after SC injection
- Days 5, 8, 11, 22, 29, 85, and
- early discontinuation if necessary.

[r] Time points for exploratory serum sampling for IV dosing cohorts with time windows in parentheses:

- Day 1: predose (-10 minutes), end of infusion (±5 minutes), and at 30 minutes (±5 minutes), 1 hour (±5 minutes), 2 hours (±10 minutes), 6 hours (±10 minutes), and 12 hours (±15 minutes) after the end of IV infusion
- Day 2: 24 hours (±15 minutes) after the end of IV infusion
- Days 8, 29, 85, and
- early discontinuation if necessary.

The scheduled exploratory serum sampling time points for IV administration are the same as the scheduled PK sampling time points for IV administration on days when they are concurrently scheduled.

[s] Time points for exploratory serum sampling for SC dosing cohort with time windows in parentheses:

- Day 1: predose (-10 minutes), and 6 hours (±10 minutes) and 12 hours (±15 minutes) after SC injection
- Day 2: 24 hours (±15 minutes) after SC injection
- Days 5, 8, 11, 22, 29, 85, and
- early discontinuation if necessary.

The scheduled exploratory serum sampling time points for SC administration are the same as the scheduled PK sampling time points for SC administration on days when they are concurrently scheduled.

Study Schedule for Protocol J1B-MC-FRCC (Part B: Repeat-Dose Design in Healthy Volunteers)

Procedures/Assessments	Screening	Baseline/Dosing						Postdosing Follow-up					
Visit Number	V1	V2		V3	V4	V5		V6	V7	V8	V9	V10	ED
Study Day(s)	-D28 to -D2	1	2	4 ± 1 d	8 ± 1 d	15	16	18 ± 1 d	22 ± 2 d	29 ± 3 d	57 ± 3 d	99 ± 3 d	
Week(s)		0			1	2			3	4	8	14	
Informed consent	X												
Review/confirm I/E criteria	X	X[a]											
Admission to CRU[b]		X				X							
Discharge from CRU[b]			X				X						
Clinical Assessments													
Complete medical history	X												
Review preexisting conditions/AEs	X	X[a]	X	X	X	X[a]	X	X	X	X	X	X	X
Concomitant medications	X	X[a]	X	X	X	X[a]	X	X	X	X	X	X	X
Substance use (alcohol, tobacco use)	X												
Physical examination[c]	X	X				X						X	X
Weight	X											X	X
Height	X												
Vital signs (heart rate, blood pressure, and temperature)[d,e]	X	X[a]	X	X	X	X[a]	X	X	X	X	X	X	X
ECGs[e,f]	X	X	X			X	X			X	X	X	X
Chest x-ray[g] (posterior-anterior view)	X												
IP administration (IV)		X				X							
QuantiFERON®-TB Gold test or TST[h]	X												

Procedures/Assessments	Screening	Baseline/Dosing						Postdosing Follow-up					
Visit Number	V1	V2		V3	V4	V5		V6	V7	V8	V9	V10	ED
Study Day(s)	-D28 to -D2	1	2	4 ± 1 d	8 ± 1 d	15	16	18 ± 1 d	22 ± 2 d	29 ± 3 d	57 ± 3 d	99 ± 3 d	
Week(s)		0			1	2			3	4	8	14	
Laboratory Assessments[i]													
HIV/Hepatitis B (surface antigen and core antibody)/C Testing	X												
Serum immunoglobulins (IgG, IgM, IgA)	X					X				X		X	X
Urinary drug screen	X												
FSH[j]	X												
Pregnancy test[j]	X	X				X				X	X	X	X
Clinical chemistry	X	X			X	X			X	X	X	X	X
Hematology	X	X			X	X			X	X	X	X	X
Urinalysis	X	X			X	X			X	X	X	X	X
Pharmacogenetics		X[k]											
Immunogenicity[l]		X[k]				X[k]				X		X	X
Blood samples for flow cytometry (safety)		X[k]			X	X[k]				X	X		
LY3454738 concentration (PK)[i,l,m]		X	X	X		X	X	X	X	X	X	X	X
Receptor Occupancy[i,n]		X	X	X		X	X	X	X	X		X	X
Exploratory serum samples[i,o]		X	X	X		X	X	X	X	X		X	X

Abbreviations: AE = adverse event; CRU = clinical research unit; D = study day; d = days; ECG = electrocardiogram; ED = early discontinuation; FSH = follicle-stimulating hormone; HIV = human immunodeficiency virus; I/E = inclusion/exclusion; Ig = immunoglobulin; IP = investigational product; IV = intravenous; PK = pharmacokinetic; TB = tuberculosis; TST = tuberculin skin test; V = visit.

[a] At the discretion of the clinical site, the baseline measurement for this assessment can be collected at any time prior to dosing on the day of dosing.

[b] Admission to the CRU may occur on the day prior to dosing or during morning of the day of dosing at the discretion of the CRU, and discharge from the CRU should occur following completion of the Day 2 and Day 16 assessments.

[c] Symptom-directed physical examinations may be conducted at any time during the study, as deemed necessary by the investigator.

[d] Vital signs should be taken following an approximate 5-minute rest in supine position. Temperature measurement is required only at screening and baseline.

[e] On days with concurrent ECG, vital sign, and/or PK sampling, these measurements should occur at approximately the same time. ECG recording and vital sign measurements should occur prior to the blood draw. Participants must be supine for approximately 5 to 10 minutes before ECG collection and remain supine but awake during ECG collection.

[f] Times are referenced to end of dosing. ECGs will be obtained on Day 1 (predose [any time prior to dosing on the day of dosing, but within 90 minutes prior to the predose blood draw], end of infusion, and 6 hours after the end of infusion), Day 2 (24 hours after the end of dosing) and at any time on other specified visits (but prior to any blood draws at same visit).

[g] A posterior–anterior chest x-ray will be performed at screening unless one has been performed within the past 6 months and the x-ray and reports are available.

[h] The follow-up TST reading should occur 2 to 3 days after V1.

[i] If more than 1 laboratory blood draw is scheduled at the same time, these blood draws should be obtained in the following order: (1) safety laboratory tests, (2) PK samples, (3) receptor occupancy samples, and (4) exploratory samples.

[j] A serum pregnancy test will be conducted at screening only. Urine pregnancy test will be used at all other time points. For women who are considered to be not of childbearing potential, FSH should be drawn to confirm status as defined in Inclusion Criterion [1b] and to be considered exempt for further pregnancy tests during the study.

[k] This activity should be completed before LY3454738 or placebo administration (predose).

[l] In the event of drug hypersensitivity reactions (immediate or nonimmediate), up to 3 additional samples will be collected each for PK and immunogenicity as close to the onset of the reaction event as possible, at the resolution of the event, and 30 days following the event.

[m] Time points for PK sampling with time windows in parentheses:

- Day 1: predose (-10 minutes), end of infusion (±5 minutes), 30 minutes (±5 minutes), 1 hour (±5 minutes), 2 hours (±10 minutes), 6 hours (±10 minutes), and 12 hours (±15 minutes) after the end of infusion
- Day 2: 24 hours (±15 minutes) after the end of infusion
- Day 4
- Day 15: predose (-10 minutes), end of infusion (±5 minutes), 30 minutes (±5 minutes), 1 hour (±5 minutes), 2 hours (±10 minutes), 6 hours (±10 minutes), and 12 hours (±15 minutes) after the end of infusion
- Day 16: 24 hours (±15 minutes) after the end of infusion
- Days 18, 22, 29, 57, 99, and
- early discontinuation if necessary.

The actual date and exact 24-hour clock time of sample collection should be recorded. Samples are requested to be taken at the specified time; however, aberrations to the specified sampling times will not be considered protocol deviations as long as the samples are taken and the actual sampling time is recorded. It is essential that the actual times of doses and samples are recorded accurately on the appropriate forms.

[n] Time points for receptor occupancy sampling with time windows in parentheses:

- Day 1: predose (-10 minutes), end of infusion (±5 minutes), and at 30 minutes (±5 minutes), 1 hour (±5 minutes), 2 hours (±10 minutes), and 4 hours (±10 minutes) after the end of infusion
- Day 2: 24 hours (±15 minutes) after the end of infusion
- Day 4
- Day 15: predose (-10 minutes), end of infusion (±5 minutes), and at 30 minutes (±5 minutes), 1 hour (±5 minutes), 2 hours (±10 minutes), and 4 hours (±10 minutes) after the end of infusion
- Day 16: 24 hours (±15 minutes) after the end of infusion
- Days 18, 22, 29, 99, and
- early discontinuation if necessary.

[o] Time points for exploratory serum sampling with time windows in parentheses:

- Day 1: predose (-10 minutes), end of infusion (±5 minutes), 30 minutes (±5 minutes), 1 hour (±5 minutes), 2 hours (±10 minutes), 6 hours (±10 minutes), and 12 hours (±15 minutes) after the end of infusion
- Day 2: 24 hours (±15 minutes) after the end of infusion
- Day 4
- Day 15: predose (-10 minutes), end of infusion (±5 minutes), 30 minutes (±5 minutes), 1 hour (±5 minutes), 2 hours (±10 minutes), 6 hours (±10 minutes), and 12 hours (±15 minutes) after the end of infusion
- Day 16: 24 hours (±15 minutes) after the end of infusion
- Days 18, 22, 29, 99, and
- early discontinuation if necessary.

The scheduled exploratory serum sampling time points are the same as the scheduled PK sampling time points on days when they are concurrently scheduled.

Study Schedule for Protocol J1B-MC-FRCC (Part C: Repeat Dose Design in Participants with Atopic Dermatitis)

Procedures/Assessments	Screening	Baseline/Dosing								Post-dosing Follow-Up			
**Visit Number**	**V1**	**V2**	**V3**	**V4**	**V5**	**V6**	**V7**	**V8**	**V9**	**V10**	**V11**	**V12**	**ED**
**Study Day(s)**	**-D28 to -D2**	**1**	**8 ± 1 d**	**15 ± 1 d**	**29 ± 1 d**	**43 ± 1 d**	**57 ± 1 d**	**71 ± 1 d**	**85 ± 1 d**	**113 ± 2 d**	**141 ± 2 d**	**169 ± 2 d**	
**Week(s)**		**0**	**1**	**2**	**4**	**6**	**8**	**10**	**12**	**16**	**20**	**24**	
Informed consent	X												
Review/confirm I/E criteria	X	X											
Clinical Assessments													
Complete medical history	X												
Review pre-existing conditions/AEs	X	X[a]	X	X[a]	X[a]	X[a]	X[a]	X[a]	X[a]	X	X	X	X
Concomitant medications	X	X[a]	X	X[a]	X[a]	X[a]	X[a]	X[a]	X[a]	X	X	X	X
Previous and current AD treatments	X												
Substance use (alcohol, tobacco use)	X												
Physical examination[b]	X												
Weight	X								X				X
Height	X												
Vital signs (heart rate, blood pressure, and temperature)[c,d]	X	X[a]	X	X[a]	X[a]	X[a]	X[a]	X[a]	X[a]	X	X	X	X
ECGs[d,e]	X	X		X	X	X	X	X	X	X	X	X	X
Chest x-ray[f] (posterior-anterior view)	X												
IP administration (IV)		X		X	X	X	X	X	X				
QuantiFERON®-TB Gold test or TST[g]	X												
Scales													
vIGA-AD	X	X	X	X	X	X	X	X	X	X	X	X	X
EASI	X	X	X	X	X	X	X	X	X	X	X	X	X
SCORAD	X	X	X	X	X	X	X	X	X	X	X	X	X
Health Outcome Measures and Other Questionnaires[h]													

Procedures/Assessments	Screening	Baseline/Dosing								Post-dosing Follow-Up			
Visit Number	V1	V2	V3	V4	V5	V6	V7	V8	V9	V10	V11	V12	ED
Study Day(s)	-D28 to -D2	1	8 ± 1 d	15 ± 1 d	29 ± 1 d	43 ± 1 d	57 ± 1 d	71 ± 1 d	85 ± 1 d	113 ± 2 d	141 ± 2 d	169 ± 2 d	
Week(s)		0	1	2	4	6	8	10	12	16	20	24	
POEM	X	X	X	X	X	X	X	X	X	X	X	X	X
DLQI	X	X	X	X	X	X	X	X	X	X	X	X	X
Laboratory Assessments[i]													
HIV/Hepatitis B (surface antigen and core antibody)/C Testing	X												
Serum immunoglobulins (IgG, IgM, IgA)	X								X			X	X
Urinary drug screen	X												
FSH[j]	X												
Pregnancy test[j]	X	X	X	X	X	X	X	X	X	X	X	X	X
Clinical chemistry	X	X	X	X	X		X		X	X	X	X	X
Hematology	X	X	X	X	X		X		X	X	X	X	X
Urinalysis	X	X	X	X	X		X		X	X	X	X	X
Pharmacogenetics		X[k]											
Immunogenicity[l]		X[k]		X[k]	X[k]				X[k]			X	X
Blood samples for flow cytometry (safety)		X[k]		X[k]	X[k]	X[k]			X[k]				
LY3454738 concentration (PK)[i,l,m]		X	X	X	X	X	X	X	X	X			X
Receptor Occupancy[i,n]		X							X				
Serum IL-19		X[k]	X	X[k]	X[k]	X[k]	X[k]	X[k]	X[k]	X	X	X	X
Skin biopsies: non-lesional and lesional[o]		X							X				
Exploratory samples[i,p]		X[q]		X	X	X			X[q]			X	

Abbreviations: AD = atopic dermatitis; AE = adverse event; D = study day; d = days; DLQI = Dermatology Life Quality Index; EASI = Eczema Area and Severity Index; ECG = electrocardiogram; ED = early discontinuation; FSH = follicle-stimulating hormone; HIV = human immunodeficiency virus; I/E = inclusion/exclusion; Ig = immunoglobulin; IL-19 = interleukin 19; IP = investigational product; IV = intravenous; mRNA = messenger ribonucleic acid;

PK = pharmacokinetic; POEM = Patient-Oriented Eczema Measure; SCORAD = SCORing Atopic Dermatitis; TB = tuberculosis; TST = tuberculin skin test; V = visit; vIGA-AD = Validated Investigator's Global Assessment for Atopic Dermatitis.

[a] At the discretion of the clinical site, the baseline measurement for this assessment can be collected at any time prior to dosing on the day of dosing.

[b] Symptom-directed physical examinations may be conducted at any time during the study, as deemed necessary by the investigator.

[c] Vital signs should be taken following an approximate 5-minute rest in supine position. Temperature measurement is required only at screening and baseline.

[d] On days with concurrent ECG, vital sign, and/or PK sampling, these measurements should occur at approximately the same time. ECG recording and vital sign measurements should occur prior to the blood draw. Participants must be supine for approximately 5 to 10 minutes before ECG collection and remain supine but awake during ECG collection.

[e] Times are referenced to end of dosing. ECGs will be obtained on Day 1 (predose [any time prior to dosing on the day of dosing, but within 90 minutes prior to the predose blood draw], end of infusion, and 6 h after the end of infusion) and at any time on other specified visits (but prior to any blood draws at same visit).

[f] A posterior–anterior chest x-ray will be performed at screening unless one has been performed within the past 6 months and the x-ray and reports are available.

[g] The follow-up TST reading should occur 2 to 3 days after V1.

[h] The following measures (POEM and DLQI) should be completed prior to any clinical assessments being performed on days when study visits occur.

[i] If more than 1 laboratory blood draw is scheduled at the same time, these blood draws should be obtained in the following order: (1) safety laboratory tests, (2) PK samples, (3) receptor occupancy samples, and (4) exploratory samples.

[j] A serum pregnancy test will be conducted at screening only. Urine pregnancy test will be used at all other time points. For women who are considered to be not of childbearing potential, FSH should be drawn to confirm status as defined in Inclusion Criterion [1b] and to be considered exempt for further pregnancy tests during the study.

[k] This activity should be completed before IP administration (predose).

[l] In the event of drug hypersensitivity reactions (immediate or nonimmediate), up to 3 additional samples will be collected each for PK and immunogenicity as close to the onset of the reaction event as possible, at the resolution of the event, and 30 days following the event.

[m] Time points for PK sampling with time windows in parentheses:

- Day 1: predose (-10 minutes), end of infusion (±5 minutes), 30 minutes (±5 minutes), 1 hour (±5 minutes), 2 hours (±10 minutes), and 6 hours (±10 minutes) after the end of infusion
- Day 8
- Days 15, 29, 43, 57, and 71: predose
- Day 85: predose (-10 minutes), end of infusion (±5 minutes), 30 minutes (±5 minutes), 1 hour (±5 minutes), 2 hours (±10 minutes), and 6 hours (±10 minutes) after the end of infusion, and
- Follow-up PK samples will be obtained at any time during the day on Day 113 and/or ED.

For visits where PK samples will be collected, the actual date and exact 24-hour clock time of sample collection should be recorded. Samples are requested to be taken at the specified time; however, aberrations to the specified sampling times will not be considered protocol deviations as long as the samples are taken and the actual sampling time is recorded. It is essential that the actual times of doses and samples are recorded accurately on the appropriate forms.

[n] Time points for receptor occupancy sampling on Days 1 and 85 with time windows in parentheses: predose (-10 minutes), end of infusion (±5 minutes), and 30 minutes (±5 minutes), 2 hours (±10 minutes), and 4 hours (±10 minutes) after the end of IV infusion.

[o] At baseline (Visit 2), lesional and non-lesional skin biopsies will be obtained. At Visit 9, only lesional skin biopsies will be obtained.

[p] Exploratory blood samples will be collected for serum, plasma, and mRNA samples except where indicated (footnote q). Samples collected predose (-10 minutes), except where indicated (footnote q).

[q] Serum only on Days 1 and 85 with time windows in parentheses: end of infusion (±5 minutes), and 30 minutes (±5 minutes), 2 hours (±10 minutes), and 4 hours (±10 minutes) after the end of IV infusion.

3. Introduction

3.1. Study Rationale

This study is a first-in-human trial designed to evaluate safety, tolerability, pharmacokinetics (PK), and target engagement of LY3454738 in healthy participants and participants with atopic dermatitis (AD), with additional assessments of clinical pharmacodynamics (PD) and efficacy in participants with AD. The inclusion of healthy Japanese participants in this study will facilitate the inclusion of Japanese participants in subsequent clinical trials. The trial will be triple-blind and placebo-controlled in order to avoid bias in the collection and evaluation of data and to enable characterization of adverse events (AEs).

The study is designed in 3 parts, with staggered initiation. Part A will assess LY3454738 in single-ascending doses across a planned dose range to encompass the predicted clinical efficacious exposure. Part B will assess repeat dosing of LY3454738 at a dose level expected to have submaximal exposure in order to assess any acute safety/tolerability events associated with a second exposure. Parts A and B will be conducted in a confined Clinical Research Unit (CRU) in healthy participants to maximize appropriate safety oversight and to assess the safety, tolerability, PK, and target engagement of LY3454738 in a setting with minimal physiologic variability that disease states could introduce. Part C will assess repeat dosing of LY3454738 at a single dose level predicted to give efficacious exposures in participants with AD, in order to evaluate its impact on clinical PD and efficacy. This part of the study will be conducted in an ambulatory setting, given that steady state exposure achieved over the dosing interval in Part C will not exceed the exposure achieved and previously assessed for safety/tolerability in Part A. Dosing will primarily be intravenous (IV) to maximize achieving the predicted efficacious exposures at this early stage of development. A single subcutaneous (SC) cohort will be included in Part A to provide an estimate of relative bioavailability, given that SC dosing will be the preferred route to treat target populations. The planned dose levels, frequency, and distribution of Japanese and non-Japanese participants may be modified based on emerging data collected during the study.

Atopic dermatitis is primarily driven by a Th2-mediated immune response magnified by pruritic inflammatory skin. The target for LY3454738, the CD200 receptor (CD200R), is expressed on many of the known key cell types important in AD pathogenesis (Broderick et al. 2002; Wright et al. 2003). Further, CD200R expression is increased in lesional skin of patients with AD (Blom et al. 2017). CD200R ligand or agonist antibodies (including LY3454738) inhibit immune cell activation and CD200R agonist antibodies have also demonstrated robust in vivo efficacy in preclinical skin inflammation models (see the LY3454738 Investigator's Brochure [IB] for additional details). Data generated from Part C will guide the future development of the compound in AD.

3.2. Background

LY3454738 is a humanized immunoglobulin G subclass 4 (IgG4)-variant monoclonal antibody (mAb) that binds (affinity: 5.6 nM) and agonizes the human inhibitory checkpoint CD200R. CD200R is an immunoglobulin (Ig) superfamily transmembrane glycoprotein expressed on the

surface of myeloid cells (mast cells, basophils, macrophages, and dendritic cells [DCs]), and also on T cells, B cells, neutrophils, and microglia (Broderick et al. 2002; Wright et al. 2003). The CD200R ligand (CD200) is also an Ig superfamily transmembrane glycoprotein, expressed on the surface of a variety of cell types including vascular endothelial cells, fibroblasts, T and B cell subsets, and neurons (Wright et al. 2003). Unlike many other immune inhibitory receptors, CD200R does not contain a classic immunoreceptor tyrosine-based inhibitory motif (ITIM), but instead contains 3 cytoplasmic tyrosine residues, 2 of which are located in a phosphotyrosine binding domain recognition motif (NPxY). Stimulation by its ligand CD200, leads to the phosphorylation of these tyrosines by Src kinases, leading to phosphorylation of docking protein (Dok)1 and Dok2, binding of Ras GTPase-activating protein 1 (RasGAP) and SH2-containing inositol 5' phosphatase (SHIP), and subsequent downstream inhibition of the Ras-mitogen activated protein kinase (MAPK) pathways, which leads to decreased cell function (cytokine release and proliferation) (Zhang et al. 2004).

Binding of LY3454738 to CD200R induces a negative signal that results in functional inhibition of the target cell activity. LY3454738 has been tested in in vitro bioassays, and surrogate antibodies have been tested in in vivo models of cutaneous skin disease in rodents, while LY3454738 has been assessed in a skin inflammation model in cynomolgus monkeys (affinity for cynomolgus monkey CD200R: 2.3 nM versus 5.6 nM for human CD200R). In in vitro assays, LY3454738 demonstrated activity in reducing both cell activation and inflammatory cytokine production, as well as in the suppression of T cell proliferation. In addition, the projected human PK and receptor occupancy (RO) data support every-other-week, or less frequent, administration in patients (see the LY3454738 IB for additional details).

Both CD200R and CD200 deficient mice have a normal phenotype, but are more prone to induced autoimmune disease (Hoek et al. 2000, Simelyte et al. 2010). Conversely, CD200 overexpression in mice provides resistance to allogeneic transplantation rejection (Yu et al. 2013) and dextran sulfate sodium (DSS)-induced colitis (Chen et al. 2016). Several viruses use homologs of CD200 to suppress the immune system and evade immune elimination by engaging CD200R (Farré et al. 2017).

Atopic dermatitis is characterized by impaired skin barrier function, skin inflammation secondary to a predominant Th2 immune response, and immunoglobulin E (IgE)-mediated sensitization to food and environmental allergens. The disease, especially in adults, is frequently refractory to adequate topical treatment with mid- to high-potency corticosteroids and/or calcineurin inhibitors. Long-term treatment with oral immunosuppressive therapy is often required to control the burden of disease, prevent flare-ups and achieve better patient quality of life outcomes (Megna et al. 2017). Dupilumab, a mAb that binds to the interleukin (IL)-4Ra subunit that is required for binding IL-4 or IL-13, is the only approved biologic for AD. The pathogenesis of AD is orchestrated by both the adaptive and innate immune systems (Egawa and Weniger 2015). Skin-resident cells such as keratinocytes, DCs, mast cells, macrophages, and innate lymphoid cells (ILCs) contribute to skin inflammation. In addition, T cells, plasmacytoid dendritic cells (pDCs), monocytes, and granulocytes, which are recruited from blood circulation, also contribute to the pathology (Weninger et al. 2014).

Immune checkpoint regulators like CD200R are critical modulators of the immune system, allowing the initiation of a productive immune response and preventing the onset of autoimmunity by negatively regulating the response once the pathogen is eliminated. CD200R is expressed on cell types that are key contributors to the pathology of AD. The target expression pattern on pathologic cell types in cutaneous inflammation together with consistent efficacy of CD200R agonistic antibodies in mouse and cynomolgus monkey preclinical models of skin inflammation provides substantial support for treating AD with LY3454738 (see the LY3454738 IB for additional details).

3.3. Benefit/Risk Assessment

The nonclinical safety information for LY3454738 supports the transition from preclinical status to the planned clinical study. The toxicological potential of LY3454738 was assessed in cynomolgus monkeys, a pharmacologically relevant species, administered the molecule for 3 months; additionally, the potential for cytokine release syndrome was evaluated in vitro with human whole blood and peripheral blood mononuclear cells, and the data generated support a low risk of cytokine release in the clinic. These studies indicate an acceptable safety profile and margin to support initial clinical investigation. While LY3454738 has agonist action on CD200R to achieve immune suppressive effects, it is notable that nonclinical toxicology studies of immune-enhancing molecules that act by antagonizing inhibitory checkpoint receptors (eg programmed cell death protein 1 [PD-1], programmed death ligand 1 [PD-L1], cytotoxic T-lymphocyte associated protein [CTLA]) have under-predicted AEs experienced clinically (Naidoo et al. 2015; Saber et al. 2016).

LY3454738 binding to CD200R does not block binding of the ligand (CD200). Both LY3454738 and ligand can bind at the same time and induce activity through CD200R, resulting in the inhibitory signaling cascade. All of the preclinical data support LY3454738 as an agonist to CD200R resulting in induction of the inhibitory cascade, and there is no evidence of any immune activation due to LY3454738 binding to CD200R (see the LY3454738 IB for additional details).

The risk of cytokine release syndrome is considered to be low given that LY3454738 is an agonist of an inhibitory immune checkpoint receptor and has an IgG4-P backbone with low capacity for Fc-mediated cross-linking to cause innate immune cell activation or depletion through antibody-dependent cellular cytotoxicity (ADCC) and/or complement. Importantly, LY3454738 did not produce FcγRIIIa activity or complement-dependent cytotoxicity (CDC) in vitro, and did not induce cytokine release when evaluated alongside negative control and positive control agents in in vitro cytokine release assays using human whole blood or in peripheral blood mononuclear cells (see the LY3454738 IB for additional details).

A repeat-dose toxicity study was conducted in cynomolgus monkeys (Study 20135344) in which LY3454738 was administered once-weekly for 3 months as an SC dose of 0, 15 or 50 mg/kg or an IV dose of 170 mg/kg (13 total doses with necropsy occurring 1 week after the final dose on Day 85). The study included standard toxicokinetic, toxicology, and safety pharmacology (central nervous system, cardiovascular, and respiratory) evaluations and immunotoxicity

endpoints (peripheral blood immunophenotyping). There were no adverse effects related to LY3454738 and a no-observed-adverse-effect level (NOAEL) was established as 170 mg/kg IV. Treatment-related findings were limited to non-adverse inflammation observed histologically at IV administration sites (170 mg/kg group), commonly observed in monkeys following repeated IV injections. Minor and/or inconsistent decreases in total leukocytes, neutrophils, monocytes, and/or lymphocytes were observed in individual animals of the low and mid dose groups and were of uncertain relationship to LY3454738 (see the LY3454738 IB for additional details).

This protocol reflects the fact that LY3454738 has not been administered to humans previously, and the study has been designed to be conducted in accordance with principles outlined in EMA (2017). Any identified risks are considered to be monitorable and manageable at the planned dose range of 1 to 1000 mg for LY3454738 in healthy participants and 500 mg biweekly in participants with AD. Levels of specific immune cell populations will be checked throughout the study per the Schedule of Activities (Section 2) and all participants will be monitored for AEs at every visit and managed appropriately as needed.

More information about the known and expected benefits, risks, serious adverse events (SAEs) and reasonably anticipated adverse events (AEs) of LY3454738 are to be found in the IB.

4. Objectives and Endpoints

Table FRCC.1 shows the primary, secondary, and exploratory objectives of the study.

Table FRCC.1. Objectives and Endpoints

Objectives	Endpoints
**Primary**	
• To assess the safety and tolerability of LY3454738 after single IV and SC dosing in healthy participants and after multiple IV dosing in healthy participants and participants with AD	Incidence of AEs, TEAEs, and SAEs
• To evaluate the efficacy of LY3454738 after multiple IV dosing in participants with AD at Week 12	Proportion of participants achieving a vIGA-AD score of 0 or 1 with a ≥ 2-point improvement from baseline at Week 12.
**Secondary**	
• To characterize the PK of LY3454738 following single (IV and SC) and multiple (IV) dosing administration in healthy participants and after multiple IV dosing in participants with AD	C_{max} and AUC
• To evaluate the efficacy of LY3454738 over time after multiple IV dosing in participants with AD	At Week 1 through Week 12 and/or early discontinuation, the proportion of participants achieving:<br>• vIGA-AD of 0 or 1 with a ≥2-point improvement from baseline,<br>• 50%, 75%, and 90% reduction from baseline in EASI, and 50%, 75%, and 90% improvement from baseline in SCORAD<br><br>Mean change from baseline in EASI and SCORAD at Week 1 through Week 12 and/or early discontinuation
**Exploratory**	
• To assess the potential development of anti-LY3454738 antibodies and their impact on safety and PK of LY3454738	Presence of ADA against LY3454738
• To evaluate relationship of receptor occupancy versus dose after single and multiple dosing of LY3454738	Percent receptor occupancy based on LY3454738 binding to neutrophils from whole blood
• To explore the PD response of LY3454738 after multiple IV dosing in participants with AD	Classical histology, immunohistochemistry, mRNA expression in biopsies, and serum IL-19
• To evaluate the efficacy of LY3454738 over time after multiple IV dosing in participants with AD as assessed by PRO/QoL measures	DLQI and POEM
• To explore the potential associations between exposure and PD/clinical responses	Exposure-response model parameters for select biomarkers and clinical efficacy endpoints

<table>
<tr><th>Objectives</th><th>Endpoints</th></tr>
<tr><td><u>Exploratory (continued)</u>
• To explore the safety, tolerability, PK, and receptor occupancy of LY3454738 in Japanese participants in relation to non-Japanese participants.</td><td>
Parameters may be separated by race for comparison:
• Frequency of TEAEs
• C_{max}, AUC
Titer and incidence of anti-LY3454738 antibodies, including TE ADA+</td></tr>
</table>

Abbreviations: AD = atopic dermatitis; ADA = antidrug antibodies; AE = adverse event; AUC = area under the concentration versus time curve; Cmax = maximum observed drug concentration; DLQI = Dermatology Life Quality Index; EASI = Eczema Area and Severity Index; IL-19 = interleukin 19; IV = intravenous; mRNA = messenger ribonucleic acid; PD = pharmacodynamic; PK = pharmacokinetics; POEM = Patient Oriented Eczema Measure; PRO = patient-reported outcome; QoL = quality of life; SAE = serious adverse event; SC = subcutaneous; SCORAD = SCORing Atopic Dermatitis; TE ADA+ = treatment-emergent antidrug antibody positive; TEAE = treatment-emergent adverse event; vIGA-AD = Validated Investigator's Global Assessment for Atopic Dermatitis.

5. Study Design

5.1. Overall Design

Study J1B-MC-FRCC (FRCC) is a Phase 1, multicenter, randomized, placebo-controlled, triple-blind (ie, blinded to investigator, participant, and sponsor staff who are involved in the treatment or clinical evaluation of the participants), single-ascending dose (SAD) and repeat-dose trial in healthy participants and in participants with AD to explore the safety, tolerability, PK, target engagement, and, in participants with AD, clinical PD and efficacy of LY3454738. A portion of the healthy participants will be Japanese.

There are 3 parts to this trial:

- Part A: SAD design in healthy participants (including healthy Japanese participants)
- Part B: Repeat-dose design in healthy participants (including healthy Japanese participants)
- Part C: Repeat-dose design in participants with AD.

Table FRCC.2 provides a detailed description of participant cohorts and planned doses for Parts A, B, and C. Figure FRCC.1 illustrates the study design and demonstrates the relationship among Parts A, B, and C. Part A will begin first and will inform and thereby trigger the initiation of Parts B and C.

In Parts A and B of the trial, participants may be admitted on the day prior to dosing or during morning of the day of dosing at the discretion of the CRU. Participants will receive LY3454738 or placebo on Day 1 of Part A and Days 1 and 15 of Part B. While not mandatory, participants will be asked not to eat breakfast prior to arriving at the clinical site for dosing, although they may be admitted to the CRU on the evening prior to Day 1. Participants will undergo the study assessments specified in the Schedule of Activities (Section 2) and may be discharged 24 hours after dose administration on Day 2 (Part A) and on Days 2 and 16 (Part B). In case of safety concerns, participants may be required to stay at the clinical site for a longer period at the discretion of the investigator. Participants will return to the clinical site for outpatient visits for procedures specified in the Schedule of Activities (Section 2).

In Part C of the trial, participants will receive LY3454738 or placebo every 2 weeks for 12 weeks on an outpatient basis. While not mandatory, participants will be asked not to eat breakfast prior to arriving at the clinical site for dosing and will undergo the study assessments specified in the Schedule of Activities (Section 2). Participants will come to the clinical site on the morning of each investigational product (IP) administration and will remain at the site until completion of all required post-dosing activities or for at least 30 minutes following the completion of the infusion, whichever is longer. The IP will be administered as a slow IV infusion over at least 30 minutes at a maximum dose of 500 mg per hour (ie, 120 minutes for the 1000-mg dose). Participants will return to the clinical site every 2 weeks for IP administration, in addition to other scheduled visits, to undergo the assessments specified in the Schedule of Activities. Upon completion of the dosing period, follow-up visits will be performed according to the Schedule of Activities (Section 2).

Table FRCC.2. Summary of Participant Cohorts

Cohort #	Planned Dose/ Administration Route	Number of Planned non-Japanese Participants		Number of Planned Japanese Participants		Total Number of Planned Participants
		LY	PBO	LY	PBO	
**Part A**						
SAD Cohort 1[a]	1 mg/IV	6	2	0	0	8
SAD Cohort 2[a]	5 mg/IV	6	2	0	0	8
SAD Cohort 3	25 mg/IV	6	2	0	0	8
SAD Cohort 4a	100 mg/IV	3	1	3	1	8
SAD Cohort 4b	100 mg/SC	3	0	3	0	6
SAD Cohort 5	300 mg/IV	3	1	3	1	8
SAD Cohort 6	1000 mg/IV	3	1	3	1	8
**Part B**						
Repeat-Dose Cohort	200 mg/IV	3	1	3	1	8
**Part C**						
Atopic Dermatitis	500 mg/IV	20	10	0	0	30

Abbreviations: IV = intravenous; LY = LY3454738; PBO = placebo; SAD = single-ascending dose; SC = subcutaneous.

[a] Sentinel dosing will be used in this cohort. Single-ascending dose cohorts 1 and 2 will include sentinel dosing for 2 participants (1 placebo and 1 LY3454738). If safety and tolerability are acceptable in these 2 participants, the remainder of the cohort can commence dosing 48 hours later.

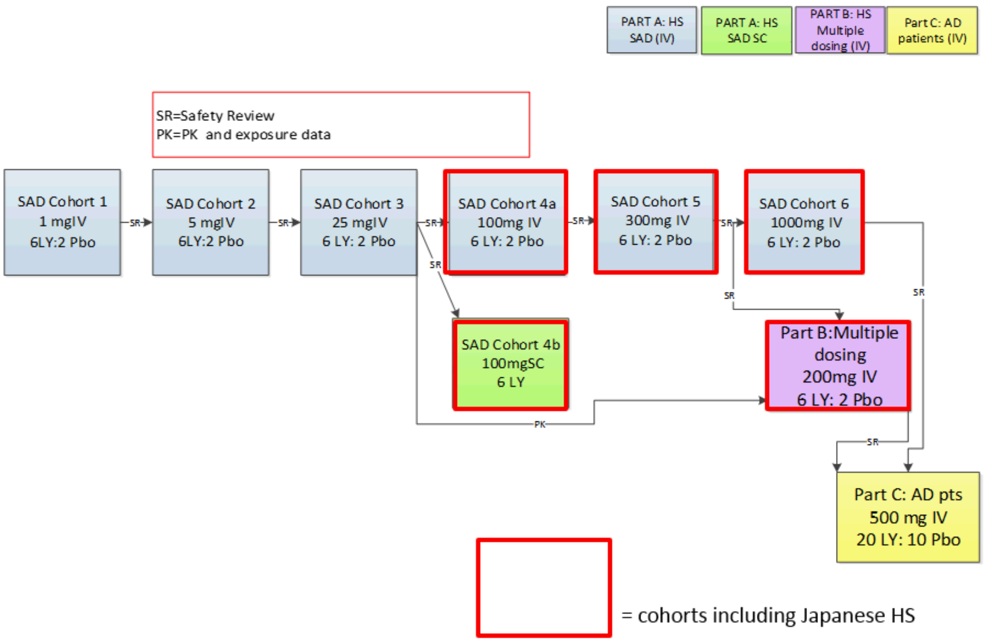

Abbreviations: AD = atopic dermatitis; HS = healthy subjects; IV = intravenous; LY = LY3454738; Pbo = placebo; PK = pharmacokinetics; pts = patients; SAD = single-ascending dose; SC = subcutaneous; SR = safety review.
SAD Cohorts 1 and 2 will include sentinel dosing for 2 participants (1 placebo and 1 LY3454738). If safety and tolerability are acceptable in these 2 participants, the remainder of the cohort can commence dosing 48 hours later.
As part of the safety review, exposure data up to and including the "one dose removed" prior cohort will be included. Additional details are provided in Section 5.1.1.
Exposure data from at least 6 participants in Cohort 5 (Part A) through Day 8 is required before initiation of Part B.

Figure FRCC.1. J1B-MC-FRCC study design.

5.1.1. Part A (SAD Design in Healthy Participants)

Single-ascending doses will be evaluated in Part A in healthy participants. Sentinel dosing will be used for 2 participants (1 placebo and 1 LY3454738) at each of the first 2 dose levels. With sentinel dosing, only 1 participant will be dosed at a time, and the second participant will not be dosed until the first participant's infusion is complete. If safety and tolerability are acceptable in these 2 participants, the remainder of the cohort can commence dosing 48 hours later. Non-sentinel participants will be dosed in sequence, each following, at a minimum, completion of the infusion for the prior participant.

Six cohorts with healthy participants are planned to be randomized to receive single IV doses of LY3454738 (1, 5, 25, 100, 300, 1000 mg) or placebo. Single-ascending dose Cohorts 4a, 5, and 6 are planned to include Japanese participants (4 per cohort [3 LY3454738 : 1 placebo]). A single open-label cohort (Cohort 4b) is planned to be enrolled to receive a single SC dose of 100 mg. Cohort 4b will include 6 healthy participants: approximately 3 non-Japanese participants and 3 Japanese participants. All participants in this cohort will receive LY3454738. This cohort

can run in parallel with Cohort 4a, which will receive a single IV dose of 100 mg. Participants will be followed for 12 weeks after dosing.

Decisions regarding dose escalation in Part A will be made by the safety review committee after safety, tolerability, and exposure are reviewed as described in Section 7.4.1.

5.1.2. Part B (Repeat-Dose Design in Healthy Participants)

A single cohort with healthy participants will be randomized to receive 2 biweekly IV doses of LY3454738 (200 mg) or placebo (Days 1 and 15). Part B will include healthy participants (4 Japanese participants [3 LY3454738 : 1 placebo] and 4 non-Japanese participants [3 LY3454738 : 1 placebo]). The planned dose for this cohort is 200 mg; a dose level expected to have submaximal exposure (Table FRCC.3) in order to assess any acute safety/tolerability events associated with a second exposure. The actual dose may be adjusted up or down based on the data review analysis (Section 7.4.2). The expected exposure at the end of dosing (area under the concentration versus time curve from time 0 to 336 hours [AUC(0-336)]) in Part B (based on predicted accumulation) must have already been evaluated as a single dose. Participants will be dosed in sequence, each following, at a minimum, completion of the infusion for the prior participant. Specific requirements for commencement of Part B are discussed in Section 7.4.2.

Participants will be followed for 12 weeks after the final treatment administration.

5.1.3. Part C (Repeat-Dose Design in Participants with AD)

A single cohort of participants with AD will be randomized 2:1 to receive IV dosing of LY3454738 or placebo, respectively, every 2 weeks for 12 weeks (dosing on Days 1, 15, 29, 43, 57, 71, and 85). The planned dose for this cohort is 500 mg. This dose is predicted to achieve a steady state exposure that is similar to the maximum exposure achieved after the maximum dose tested as a single dose (1000 mg). The actual dose in Part C may be modified after review of the prior exposure data and the PK and RO data from Cohorts 1, 2, and 3 in Part A (Section 10.3.7). Specific requirements for commencement of Part C are discussed in Section 7.4.3. If more than 1 participant is at an individual site, these participants should be dosed in sequence.

Participants will be followed for 12 weeks after the last dose.

5.2. Number of Participants

5.2.1. Part A and Part B (Healthy Participants)

Enrollment for Parts A and B will occur to enable completion of approximately 62 participants (54 participants and 8 participants in Part A and Part B, respectively) in these parts of trial (ie, completion of all scheduled procedures up to and including Day 85 in Part A and Day 99 in Part B).

The planned number of participants to complete each cohort and the planned distribution of Japanese and non-Japanese participants are summarized in Table FRCC.2. If the recruitment of Japanese participants becomes limiting for a particular cohort, then additional non-Japanese participants may be entered into the cohort to allow for dose escalation, to start the next part of

the study, or to progress to the next study. Japanese participants may subsequently be enrolled to fulfill the intended number. Although not required, it is preferred that the non-Japanese participants are non-Asian to allow for robust analyses. Planned distribution of Japanese and non-Japanese participants may be modified based on emerging data collected during the study. Participants randomized to Part A who discontinue prior to IP administration may be replaced. Participants who drop out of Part B prior to administration of the second dose of IP may be replaced. Participants who drop out following administration of the second dose or who withdraw from the trial due to safety reasons may be replaced at the discretion of the investigator and sponsor. The replacement participant should be assigned to the same treatment arm as the discontinued participant and, where possible, should be Japanese or non-Japanese to match the discontinued participant.

Refer to the Schedule of Activities (Section 2) for data to be collected at the time of discontinuation and follow-up.

5.2.2. Part C (Participants with AD)

Enrollment for Part C will occur to enable completion of approximately 30 participants in this part of the trial (ie, completion of all scheduled procedures up to and including Day 169). Participants who drop out of the trial may be replaced to meet the study objectives, or at the discretion of the sponsor. The replacement participant should be assigned to the same treatment arm as the discontinued participant.

Refer to the Schedule of Activities (Section 2) for data to be collected at the time of discontinuation and follow-up.

5.3. End of Study Definition

End of the study is the date of the last visit or last scheduled procedure shown in the Schedule of Activities (Section 2) for the last participant in the trial globally.

5.4. Scientific Rationale for Study Design

This trial is a first-in-human study and will include both single and repeat dosing. Single dosing will be assessed in healthy participants using a dose-escalation design (Part A). Repeat dosing at a single dose level of LY3454738 will be assessed in healthy participants in Part B. Repeat dosing of LY3454738 (every 2 weeks for 12 weeks) at a single dose level predicted to give efficacious exposures will be assessed in participants with AD (Part C).

All parts of the trial will be placebo controlled and triple-blind (except for a limited number of sponsor personnel) in order to avoid bias in the collection and evaluation of data. Placebo has been chosen as the control treatment to assess whether any observed effects are treatment-related or simply reflect the trial conditions. A population of healthy participants was selected to assess the PK, safety, and tolerability of LY3454738 in Parts A and B, given that healthy participants have physiologic reserve and there is substantial margin of safety of the compound based on preclinical toxicity data in cynomolgus monkeys (see Section 5.5). Further, healthy participants have a likelihood of less physiologic variability in the absence of disease states that may affect

multiple organ systems and are usually devoid of other confounding factors, such as concomitant medications.

Part A will use a dose-escalation design using IV administration. Although not considered a compound with high uncertainty (DeGeorge et al. 2018), since LY3454738 is an immune modulator and requires Fcγ receptor engagement for activity, sentinel dosing is planned for the first 2 cohorts in Part A to assess for any unanticipated acute tolerability or safety concerns. Planned dosing increments approximate 5-fold in the lower doses and reduce to approximately 3-fold in higher doses, deemed appropriate given this is not a compound with high uncertainty. Safety reviews will occur between each dose escalation using safety data as the primary criteria for dose-escalation decisions, a reasonable approach given that the safety margins are high (see Section 5.5) and the confidence in PK predictions based on IV dosing of mAbs. Assessment of the LY3454738 concentrations through 1 week postdose in the "one dose removed" prior cohort will be included in safety reviews to monitor actual exposures relative to predicted (see Section 7.4). In addition, data reviews are planned to assess exposure/RO relationship relative to safety and tolerability, to confirm predictions for planned doses for the remaining cohorts in Part A and those for Parts B and C (see Section 10.3.7 for detail). A single cohort of participants will receive LY3454738 via the SC route in Part A, which is appropriate to assess both the initial safety profile of the drug and to estimate the SC bioavailability, while minimizing participant risk. Given that this is a therapeutic antibody with anticipated half-life of 10 to 14 days, safety and tolerability will be assessed after extended exposure duration following single-dose administration across the entire range of exposures planned.

Repeat dosing of a dose predicted to give submaximal exposures will be assessed in Part B in order to assess any acute safety/tolerability events associated with a second exposure. The predicted exposure at this dose level will have previously been assessed for safety and tolerability after a single dose. Parts A and B will be conducted in a confined CRU in healthy participants to maximize appropriate safety oversight and to assess the safety, tolerability, PK, and target engagement of LY3454738.

Based on the anticipated mechanism of action, target expression profile, and supporting preclinical data, LY3454738 is expected to have a therapeutic effect in reducing signs and symptoms of autoimmune diseases, including AD (Part C). Thus, an initial assessment of therapeutic effect will be assessed in AD to inform future development of the compound for this indication. This part of the study will be conducted in an ambulatory setting, given that the steady state exposure achieved over the dosing interval in Part C will not exceed the exposure achieved and previously assessed for safety/tolerability in Part A. Part C will include a single dose level, previously deemed safe and tolerable (from Parts A and B), predicted to achieve maximum exposure at steady state. This dose level is expected to have complete receptor occupancy throughout the dosing duration to maximize the probability of achieving an exposure that provides pharmacologic activity (see Rationale for Dose Selection [Section 5.5]). A 12-week treatment period is standard for AD trials; therefore, Part C will enable a comparison to the effects of other therapies.

A portion of the participants in Parts A and B will be Japanese, in order to enable inclusion of Japanese participants into the next stages of development.

5.5. Justification for Dose

The planned dose range of 1 mg to 1000 mg as a single dose and up to 500 mg every 2 weeks is designed to minimize the risk to the participants while ensuring the evaluation of an exposure range that encompasses the anticipated pharmacologically active exposure range. The dose selection is based on nonclinical pharmacology and in vitro RO and nonclinical monkey PK of LY3454738. The human PK was projected from cynomolgus monkey PK by allometric scaling and the in vitro human neutrophil RO assay in whole blood was used to quantify the LY3454738 target engagement (Table FRCC.3).

Since assessment of the clinical exposure-response relationship will use RO rather than a pharmacologic signal, exposures that provide saturation of the target over the planned dosing interval in target patient populations will be assessed to maximize that pharmacologically active exposures have been achieved. This is a reasonable approach given the substantial margin of safety and that preclinical in vitro data (reduction of cytokine production in cell lines over-expressing CD200R) and in vivo data (surrogate antibody in mouse model of contact dermatitis) indicate a dose response relationship of agonizing the CD200R (see the IB for details).

The starting dose (1 mg) is anticipated to have minimal pharmacologic activity based on the expected RO at maximal serum concentration (C_{max}=EC_{10-15}). The planned second and third dose levels, 5 mg and 25 mg, are expected to achieve approximately 43% RO and 81% RO, respectively, at the average drug concentration (C_{ave}) during the first 24 hours after drug administration (Table FRCC.3). For the subsequent cohorts in the SAD, planned doses were selected based on the predicted minimum observed drug concentration (C_{min}) over the planned dosing interval for efficacy (2 weeks) in order to achieve the exposures predicted to achieve the desired RO resulting in anticipated pharmacology and efficacy. This dosing scheme is deemed appropriate given that all participants in the SAD will be closely monitored in the first 24 hours at the clinical sites, potential acute safety/tolerability concerns will have been addressed in the first 2 cohorts, and considering the expected pharmacology of the compound (immune suppression). The dose to be assessed for efficacy in humans is targeted to achieve the saturation of receptor at C_{min} at steady state. While an every 2-week dose of 300 mg is likely to achieve this goal for a 70 kg participant based on the projection approach described above, to account for the uncertainty given the use of RO as a surrogate for pharmacological activity, an approximately 1.7x factor has been added to give a planned dose for Part C of 500 mg. A single dose of 1000 mg will allow assessment of safety and tolerability in a controlled clinical setting in healthy participants at this exposure. Therefore, 1, 5, 25, 100, 300, and 1000 mg are the planned doses for Part A with dose escalation not exceeding 1000 mg, and a 500-mg dose every 2 weeks is planned for Part C. A dose of 200 mg every 2 weeks for 2 doses per participant is planned for Part B to allow for assessment of safety and tolerability after a repeat dose in healthy participants under close monitoring conditions at a lower dose and lower exposure than that planned to assess efficacy.

Dose selection is further supported by preclinical toxicity data; LY3454738 was evaluated in cynomolgus monkeys over a 3-month dosing period, during which LY3454738 was administered once weekly at doses of 0, 15 or 50 mg/kg SC or 170 mg/kg IV (13 total doses with necropsy occurring 1 week after the final dose on Day 85). The NOAEL in this study was 170 mg/kg and provides margins of safety of ≥10,000-fold compared to the planned starting dose of 1 mg (based on mg/kg dose or estimated human exposure), 10-fold (based on mg/kg dose), and 7.6-fold (based on estimated human exposure) to the planned high single dose of 1000 mg, and ≥33-fold (based on mg/kg dose or estimated human exposure) to a repeated dose of 500 mg every 2 weeks. The details of the preclinical data are described in the IB for LY3454738.

Table FRCC.3. Planned Doses, Projected Human PK Exposure, and Projected RO

LY3454738 Exposure after Single Dose					LY3454738 Exposure at Steady State of Q2W Dosing		RO after Single Dose (%)			RO at Steady State of Q2W (%)	
IV Dose (mg)	C_{max} (ng/mL)	$C_{(ave,24\ h)}$ (ng/mL)	$C_{336\ h}$ (ng/mL)	$AUC_{(0\text{-}336\ h)}$ (ng•hr/mL)	$C_{(max,ss)}$ (ng/mL)	$C_{(336\ h\_ss)}$ (ng/mL)	RO at C_{max}	RO at $C_{(ave,24\ h)}$	RO at 336 h	RO at $C_{(max,ss)}$	RO at 336 h,ss
**1**	358.65	258.86	5.45	17286.68	364.77	6.05	14.85	11.02	0.21	15.07	0.24
**5**	1808.65	1436.06	70.56	140165.4	1911.31	101.66	48.81	42.80	3.07	50.26	4.43
**25**	9072.39	7484.15	1890.23	1150840	15019.94	6417.25	83.83	80.90	49.97	89.80	78.28
**100**	36315.38	30212.72	11068.37	5293041	70754.75	37229.58	95.70	94.83	86.46	97.82	95.80
**200**	72630.76	60425.45	22136.74	10586082	141509.51	74459.17	97.87	97.43	92.97	98.93	97.93
**300**	108963.96	90829.55	35838.35	16384373	219813.82	119824.31	98.60	98.31	95.64	99.33	98.73
**500**	181612.58	151446.92	60626.59	27478697	368897.10	202442.66	99.18	99.01	97.44	99.61	99.27
**1000**	363234.16	302990.58	122604.86	55215781	741615.31	408998.24	99.60	99.52	98.76	99.81	99.65

Abbreviations: $AUC_{(0\text{-}336\ h)}$ = area under the concentration curve of LY3454738 from time 0 to 336 hours after the starting dose; $C_{336\ h}$ = average LY3454738 concentration at 336 hours after the starting dose; $C_{(336\ h\ ss)}$ = average LY3454738 concentration at 336 hours at steady state; $C_{(ave,24\ h)}$ = average LY3454738 concentration over the first 24 hours since the starting dose; C_{max} = maximal LY3454738 concentration after dosing; $C_{(max,ss)}$ = maximal LY3454738 concentration after dosing at steady state; IV = intravenous; K = pharmacokinetic; Q2W = every 2 weeks; RO = receptor occupancy; RO at 336 h,ss = receptor occupancy at 336 hours at steady state of dosing.

6. Study Population

Eligibility of study participants will be based on the results of screening medical history, physical examination, vital signs, clinical laboratory tests, and electrocardiograms (ECGs). The nature of any conditions present at the time of the physical examination and any preexisting conditions will be documented.

Screening may occur up to 28 days prior to enrollment. Participants who are not enrolled within 28 days of screening may be subjected to an additional medical assessment and/or clinical measurements to confirm their eligibility.

Prospective approval of protocol deviations to recruitment and enrollment criteria, also known as protocol waivers or exemptions, are not permitted.

6.1. Inclusion Criteria for All Participants

Participants are eligible for inclusion in the study only if they meet all of the following criteria at screening and/or enrollment:

[1] are overtly healthy males or females, as determined by medical history and physical examination. To qualify as Japanese for the purpose of this study, the participant must be first-generation Japanese, defined as the participant's biological parents and all of the participant's biological grandparents must be of exclusive Japanese descent, and must have been born in Japan.

[1a] male participants:

agree to either remain abstinent (if this is their preferred and usual lifestyle) or use condoms with spermicide as well as 1 additional effective method of contraception during the study and for 90 days following the last dose (Section 6.3.4).

[1b] female participants:

Women of child-bearing potential who are abstinent (if this is complete abstinence, as their preferred and usual lifestyle) or in a same-sex relationship (as part of their preferred and usual lifestyle) must agree to either remain abstinent or stay in a same-sex relationship without sexual relationships with males. Periodic abstinence (eg, calendar, ovulation, symptothermal, post-ovulation methods), declaration of abstinence just for the duration of the trial, and withdrawal are not acceptable methods of contraception.

Otherwise, women of child-bearing potential must use 2 effective methods of contraception until 90 days following last dose of study drug. Abstinence or contraception must continue following completion of IP administration until their plasma concentrations are below the level that could result in a relevant potential exposure to a possible fetus, predicted to be 90 days following last dose of study drug (for additional details, see Section 6.3.4).

A. Women of child-bearing potential participating must test negative for pregnancy prior to initiation of treatment as indicated by a negative serum pregnancy test at the screening visit followed by a negative urine pregnancy test within a day prior to exposure. Women must also test negative for pregnancy prior to each dose.

B. Two effective methods of contraception (such as male or female condoms with spermicide, diaphragms with spermicide, or cervical sponges) will be used. The participant may choose to use a double-barrier method of contraception. Barrier protection methods without concomitant use of a spermicide are not a reliable or acceptable method. Thus, each barrier method must include use of a spermicide. It should be noted that the use of male and female condoms as a double-barrier method is not considered acceptable due to the high failure rate when these methods are combined.

i. Of note, 1 of the 2 methods of contraception may be a highly effective (less than 1% failure rate) method of contraception (such as combination oral contraceptives, implanted contraceptives, or intrauterine devices).

Women not of child-bearing potential, as defined by at least 1 of the following:

A. infertile due to surgical sterilization (hysterectomy, bilateral oophorectomy, or tubal ligation), congenital anomaly such as mullerian agenesis; or

B. post-menopausal – defined as either

i. A woman at least 50 years of age with an intact uterus, not on hormone therapy, who has had either

a) cessation of menses for at least 1 year, or

b) at least 6 months of spontaneous amenorrhea with a follicle-stimulating hormone >40 mIU/mL; or

ii. A woman at least 55 years of age not on hormone therapy, who has had at least 6 months of spontaneous amenorrhea; or

iii. A woman at least 55 years of age with a diagnosis of menopause prior to starting hormone replacement therapy.

C. female infertility due to other causes that have been discussed with, and accepted, by the sponsor.

[2] are between 18 (20 for Japanese participants) and 65 years of age, inclusive, at the time of screening.

[3] have a body mass index of 18.0 to 32.0 kg/m^2, inclusive, for Parts A and B, and 18.0 to 45.0 kg/m^2, inclusive, for Part C, and a minimum body weight of 50 kg.

[4] have clinical laboratory test results within normal reference range for the population or investigative site, or results with acceptable deviations that are judged to be not clinically significant by the investigator.

[5] have venous access sufficient to allow for blood sampling and administration of IP for IV administration as per the protocol.

[6] are reliable and willing to make themselves available for the duration of the study and are willing to follow study procedures.

[7] are able and willing to give signed informed consent.

[8] have blood pressure, pulse rate, and an ECG reading that is considered to be within normal limits as determined by the investigator.

6.1.1. Additional Inclusion Criteria for Part C (Participants with AD)

[9] have a diagnosis of AD at least 12 months prior to screening, as defined by the American Academy of Dermatology (Eichenfield et al. 2014).

[10] have AD, including all of the following:

a. Eczema Area and Severity Index (EASI) score ≥12 at screening (Visit 1) and at randomization (Visit 2)

b. Validated Investigator's Global Assessment for Atopic Dermatitis (vIGA-AD) score of ≥ 3 at screening (Visit 1) and at randomization (Visit 2)

c. ≥7% of body surface area (BSA) involvement at screening (Visit 1) and at randomization (Visit 2).

[11] have a history, documented by a physician and/or investigator, of inadequate response to existing topical medications within 6 months preceding screening, or a history of intolerance to topical therapy as defined by at least 1 of the following:

a. inability to achieve good disease control defined as mild disease or better (eg, vIGA-AD ≤2) after use of at least a medium potency topical corticosteroid (TCS) for at least 4 weeks, or for the maximum duration recommended by the product prescribing information (eg, 14 days for super-potent TCS), whichever is shorter. Topical corticosteroids may be used with or without topical calcineurin inhibitors (TCNIs).

b. documented history of clinically significant adverse reactions with the use of TCS such as skin atrophy, allergic reactions, or systemic effects that, in the opinion of the investigator, outweighs the benefits of retreatment.

c. Participants who failed systemic therapies intended to treat AD, such as cyclosporine, methotrexate, azathioprine, and mycophenolate mofetil, within 6 months preceding screening, will also be considered as having inadequate response to topical therapy.

[12] agree to discontinue use of the following excluded medications for at least 2 weeks prior to randomization (Visit 2) and throughout the study:

a. Topical corticosteroids or topical immune modulators (eg, tacrolimus or pimecrolimus)

b. Topical phosphodiesterase type 4 (PDE-4) inhibitor (crisaborole)

[13] have applied emollients daily for at least 14 days prior to randomization and agree to use emollient daily throughout the treatment period.

[14] are willing and able to undergo punch biopsies according to the Schedule of Activities (Section 2).

[15] Participants who are receiving chronic treatments to improve sleep should be on a stable dose for at least 2 weeks prior to screening as determined by the investigator. Sedating antihistamines (Exclusion Criterion #45c) are not permitted.

6.2. Exclusion Criteria for All Participants

Participants will be excluded from study enrollment if they meet any of the following criteria at screening and/or enrollment:

[16] are investigative site personnel directly affiliated with this study and their immediate families. Immediate family is defined as a spouse, biological or legal guardian, child, or sibling.

[17] are Lilly employees or are employees of a third-party organization involved with the study.

[18] are currently enrolled in a clinical study involving an IP or any other type of medical research judged not to be scientifically or medically compatible with this study or have received any nonbiologic IP within 30 days or 5 half-lives (whichever is longer) of their initial screening visit.

[19] have previously completed a clinical trial investigating any other molecule targeting IL-33 and/or the IL-33 receptor (ST2).

[20] have a current or recent acute active infection. For at least 30 days prior to screening, patients must have no significant symptoms including fever of 100.5°F (38°C) or above, at screening or baseline, and/or signs of confirmed or suspected infection.

[21] had any surgical procedure (except for minor surgery requiring local or no anesthesia and without any complications or sequelae) within 12 weeks prior to screening, or any planned surgical procedure scheduled to occur during the study.

[22] have received live vaccine(s) (including attenuated live vaccines) within 28 days of screening or intend to receive during the study (non-live or inactivated vaccinations are allowed).

[23] have a history or presence of multiple or severe allergies, or an anaphylactic reaction, to prescription or non-prescription drugs.

[24] have a history or presence of allergy to mAbs or to the drug excipients, or have clinically significant multiple or severe drug allergies, intolerance to TCSs, or a history of severe post-treatment hypersensitivity reactions (including, but not limited to, erythema multiforme major, linear immunoglobulin A [IgA] dermatosis, toxic epidermal necrolysis, or exfoliative dermatitis).

[25] have had serious, opportunistic, or chronic/recurring infection within 6 months prior to screening. Examples include, but are not limited to, infections requiring IV antibiotics, hospitalization, or prolonged anti-infective treatment.

[26] had any malignancy within the past 5 years. Exceptions: successfully treated basal cell skin carcinoma or squamous cell skin carcinoma, with no evidence of recurrence or metastatic disease within the 3 years prior to baseline.

[27] current smoker using >10 cigarettes or other tobacco products per day. Are unable/unwilling to stop smoking tobacco products while in the study unit. Heavy smokers (as per judgment of the investigator) should be excluded from the study.

[28] are regular users of known drugs of abuse and/or have positive findings on urinary drug tests at screening, OR an average weekly alcohol intake that exceeds 21 units per week (males) or 14 units per week (females), OR are unwilling to stop alcohol consumption during study visits/time in the research unit (1 unit of alcohol = 12 oz or 360 mL of beer; 5 oz or 150 mL of wine; 1.5 oz or 45 mL of distilled spirits).

[29] have donated blood of more than 500 mL within the previous 30 days of study screening.

[30] show evidence of active or latent tuberculosis (TB), as documented through medical history and examination, chest x-rays (posterior to anterior, read by a radiologist, pulmonologist, or designee; a lateral chest x-ray may be performed if clinically or radiologically indicated), and TB testing: either a positive tuberculin skin test (TST; defined as a skin induration >5 mm at 48 to 72 hours, regardless of Bacillus Calmette–Guérin or other vaccination history) or a positive QuantiFERON®-TB Gold test. If the QuantiFERON®-TB Gold test result is indeterminate, it may be repeated only once and, if the repeat test is indeterminate, the participant will be deemed ineligible. The choice to perform a TST or a QuantiFERON-TB Gold test will be made by the investigator according to local standard of care. **Please note if a chest x-ray has been performed in the past six months, found to be normal and the report is available, a further chest x-ray is not required** provided there is no known TB exposure since the x-ray was performed.

[31] have known hypogammaglobulinemia or a screening serum immunoglobulin G (IgG) <565 mg/dL, immunoglobulin M (IgM) <40 mg/dL, or IgA <70 mg/dL, as measured by the central laboratory.

[32] are immunocompromised.

[33] have presence of significant uncontrolled cerebro-cardiovascular (eg, myocardial infarction, unstable angina, hypertension, moderate to severe [New York Heart Association Class III/IV] heart failure, or cerebrovascular accident), respiratory, hepatic, renal, gastrointestinal, endocrine, hematologic, neurologic or neuropsychiatric disorders or abnormal laboratory values at screening that, in the opinion of the sponsor or investigator, pose an unacceptable risk to the participant if participating in the study or of interfering with the interpretation of data.

[34] intend to use herbal, over-the-counter, or prescription medication within 14 days prior to dosing and during the study, other than estrogen/progesterone as a form of hormone replacement therapy and/or oral contraceptive. Participants taking these medications should be on stable doses for at least 28 days prior to screening. Certain medications (eg, vitamin supplements) may be permitted at the discretion of the investigator.

[35] have evidence of chronic viral infection:

a. show evidence of hepatitis C and/or positive hepatitis C antibody.

b. show evidence of possible hepatitis B infection defined as a positive test for hepatitis B surface antigen (HBsAg) and/or hepatitis B core antibody (HBcAb).

c. show evidence of human immunodeficiency virus (HIV) infection and/or positive for HIV antibodies at screening.

d. have had symptomatic herpes zoster within 3 months prior to screening that constitutes (per investigator's judgment) a risk to the participant when taking the study medication or that may interfere with the interpretation of study data.

[36] in the opinion of the investigator or sponsor, are unsuitable for inclusion in the study.

6.2.1. Additional Exclusion Criterion for Part A (Healthy Participants)

[37] are not willing to receive multiple SC injections (applicable to Cohort 4b participants only).

6.2.2. Additional Exclusion Criteria for Part C (Participants with AD)

[38] are currently experiencing or have a history of other concomitant skin conditions (eg, psoriasis or lupus erythematosus) that would interfere with evaluations of the effect of study medication on AD.

[39] participants who, in the opinion of the investigator, are currently experiencing or have a history of erythrodermic, refractory, or unstable skin disease that requires frequent hospitalizations and/or IV treatment for skin infections that may interfere with participation in the study.

[40] a history of eczema herpeticum within 12 months prior to screening.

[41] a history of 2 or more episodes of eczema herpeticum in the past.

[42] participants who are currently experiencing a skin infection that requires treatment, or is currently being treated, with topical or systemic antibiotics.

Note: Participants may not be rescreened until at least 4 weeks after the date of their previous screen failure and at least 2 weeks after resolution of the infection.

[43] have any serious concomitant illness that is anticipated to require the use of systemic corticosteroids or otherwise interfere with study participation or require active frequent monitoring (eg, unstable chronic asthma).

[44] have been treated with the following therapies:

a. mAb (eg, ustekinumab, omalizumab, dupilumab, and other experimental biologic agents) within 5 half-lives prior to randomization (Visit 2).

b. received any parenteral corticosteroid administered by intramuscular or IV injection within 2 weeks prior to study entry (Visit 1) or within 6 weeks prior to planned randomization (Visit 2) or are anticipated to require parenteral injection of corticosteroids during the study.

c. have had an intra-articular corticosteroid injection within 2 weeks prior to study entry (Visit 1) or within 6 weeks prior to planned randomization (Visit 2).

Note: Intranasal or inhaled steroid use is allowed during the trial.

[45] have received the following excluded medications/treatments within 4 weeks prior to randomization (Visit 2) or plan to continue use throughout the study:

a. oral systemic corticosteroids and leukotriene inhibitors.

b. systemic immunomodulators, including, but not limited to, cyclosporine, methotrexate, mycophenolate mofetil, azathioprine, and janus kinase (JAK) inhibitors (tofacitinib, ruxolitinib).

c. sedating antihistamines, including, but not limited to, alimemazine, chlorphenamine, clemastine, cyproheptadine, diphenhydramine, hydroxyzine, ketotifen, and promethazine.

Note: Participants may use newer, less sedating antihistamines (eg, fexofenadine, loratadine, cetirizine).

d. any other systemic therapy used to treat AD or symptoms of AD (approved or off-label use).

e. phototherapy, including therapeutic phototherapy (psoralen plus ultraviolet-A, ultraviolet-B), excimer laser, or tanning beds.

6.2.3. Rationale for Exclusion of Certain Study Candidates

Exclusion Criteria [16] and [17] prevent conflict of interest in study participants. Exclusion Criteria [18] through [45] exclude medical conditions, medication intolerance, and concomitant medication use that may constitute a risk for the participant and/or may confound the assessment of study endpoints.

6.3. Lifestyle and/or Dietary Requirements

Throughout the study, participants may undergo medical assessments and review of compliance with requirements before continuing in the study.

6.3.1. Meals and Dietary Restrictions

While not mandatory, participants will be asked not to eat breakfast prior to arriving at the clinical site for dosing. A normal diet may be consumed at all other times during the study.

6.3.2. Caffeine, Alcohol, and Tobacco

Consumption of caffeine-containing beverages is permitted during the study. Participants should not consume alcohol for at least 24 hours prior to dosing and 2 days postdose. During outpatient periods, all participants should be advised to limit alcohol consumption to no more than 2 units per day. Participants should be willing and able to abide by smoking restrictions at the study site during both the in-house period and outpatient visits.

6.3.3. Activity

Participants should avoid strenuous physical activity for at least 48 hours prior to dosing until discharge from the study or completion of all study procedures. When certain study procedures are in progress at the site, participants may be required to remain supine or sitting.

6.3.4. Contraception

Men with partners of child-bearing potential must either remain abstinent (if this is their preferred and usual lifestyle) or use condoms as well as 1 additional highly effective (less than 1% failure rate) method of contraception (such as a combination of oral contraceptives, implanted contraceptives, or intrauterine devices) or effective method of contraception (such as diaphragms with spermicide or cervical sponges) for the duration of the study and until their plasma concentrations are below the level that could result in a relevant potential exposure to a possible fetus, predicted to be 90 days following the last dose of study drug.

- Men and their partners may choose to use a double-barrier method of contraception. Each barrier method must include use of a spermicide (ie, condom with spermicide, diaphragm with spermicide, female condom with spermicide). The use of male and female condoms as a double-barrier method is not considered acceptable due to the high failure rate when these barrier methods are combined.

- Periodic abstinence (eg, calendar, ovulation, symptothermal, post-ovulation methods), declaration of abstinence just for the duration of the trial, and withdrawal are not acceptable methods of contraception.
- Men with pregnant partners should use condoms during intercourse for the duration of the study and for 90 days following the last dose of study drug.
- Men should refrain from sperm donation for the duration of the study and until their plasma concentrations are below the level that could result in a relevant potential exposure to a possible fetus, predicted to be 90 days following the last dose of study drug.
- Men who are in exclusively same-sex relationships (as their preferred and usual lifestyle) are not required to use contraception.

Women of child-bearing potential must use 2 effective methods of contraception (such as male or female condoms with spermicide, diaphragms with spermicide or cervical sponges) and should be used until their plasma concentrations are below the level that could result in a relevant potential exposure to a possible fetus, predicted to be 90 days following last dose of study drug . Women and their partners may choose to use a double-barrier method of contraception. Each barrier method must include use of a spermicide. The use of male and female condoms as a double-barrier method is not considered acceptable due to the high failure rate when these methods are combined.

- Of note, one of the 2 methods of contraception may be a highly effective (less than 1% failure rate) method of contraception (such as a combination of oral contraceptives, implanted contraceptives or intrauterine devices).

6.4. Screen Failures

Individuals who do not meet eligibility criteria for participation in this study (screen failure) may not be rescreened until at least 4 weeks after the date of their previous screen failure and at least 2 weeks after resolution of a skin infection, unless the rescreening is for administrative reasons (see below). Blood tests may be repeated at the discretion of the investigator during screening without being considered a screen failure. Participants may be rescreened 1 time. When rescreening is performed, the individual must sign a new informed consent form (ICF) and will be assigned a new identification number.

Allowed rescreening for administrative reasons

An individual may be rescreened once for an administrative reason such as falling out of the screening window because of scheduling conflicts. The sponsor does not need to approve rescreening for an administrative reason. The rescreening can start immediately after the administrative reason has been resolved.

7. Treatment

7.1. Treatment Administered

LY3454738 is supplied for clinical trial use as a lyophilized powder formulation in a glass vial. After reconstitution of the vial contents containing 75 mg of LY3454738, with 3.2 mL of sterile water for IV infusion or 1.5 mL of sterile water for SC injection, LY3454738 can be administered as a clear/slightly opalescent solution at a concentration of 25 mg/mL or 50 mg/mL. To achieve the various doses proposed by this protocol, the drug product might be diluted using the sterile diluent provided by the sponsor for the study. Placebo for this study will be normal saline (0.9% NaCl).

Detailed instructions for the preparation and handling of the administered products will be provided by the sponsor in the pharmacy binder. The products must be prepared by an unblinded pharmacist who is not involved in any other study-related procedures. When prepared for dosing according to instructions, it will not be possible to distinguish between LY3454738 and placebo.

The IP will be administered either as an SC injection or as a slow IV infusion. Table FRCC.4 shows the planned dose levels and dosage formulations. For IV infusion, the IV will occur over at least 30 minutes at a maximum dose of 500 mg per hour (ie, 120 minutes for the 1000-mg dose). For SC injections, the drug product should be administered as one 2 mL injection, but can be administered as two 1 mL injections at the investigator's discretion. Injection sites selected for SC administration should be the abdominal region approximately 5 cm from the umbilicus and the treatment administered with the needle applied at approximately 45 degrees with pinching of the skin. Subcutaneous administration of LY3454738 should be given by a limited number of trial site personnel for consistency. Where multiple injections are to be administered, the subsequent injection(s) should be given within 5 minutes following the previous and administered to another abdominal quadrant.

Table FRCC.4. Treatments Administered

Treatment Name	LY3454738	Placebo
**Dosage Formulation**	Lyophilized powder	0.9% Sodium Chloride
**Dosage Levels**	1, 5, 25, 100, 200, 300, 500, and 1000 mg (IV)<br>100 mg (SC)	NA
**Routes of Administration**	IV infusion or SC injection	IV infusion or SC injection
**Dosing Instructions**	Part A[a]: Single Dose on Day 1<br>Part B: Repeat dose on Days 1 and 15<br>Part C: One dose every 2 weeks for 12 weeks	Part A: Single Dose on Day 1<br>Part B: Repeat dose on Days 1 and 15<br>Part C: One dose every 2 weeks for 12 weeks

Abbreviations: IV = intravenous; LY = LY3454738; NA = not applicable; SC = subcutaneous.

a Includes Cohort 4b, which is the only cohort in this study receiving LY3454738 SC injections.

Sites must have resuscitation equipment, emergency drugs, and appropriately trained staff available during the infusion and for at least 24 hours after participants have completed their infusion in Parts A and B of the study and until completion of all required post-dosing activities or for at least 30 minutes following the completion of the infusion (whichever is longer) in Part C.

The investigator or designee is responsible for:

- explaining the correct use of the IP(s) to the participant,
- verifying that instructions are followed properly,
- maintaining accurate records of IP dispensing and collection, and
- returning all unused medication to Lilly or its designee at the end of the study

Note: In some cases, sites may destroy the material if, during the investigative site selection, the evaluator has verified and documented that the site has appropriate facilities and written procedures to dispose of clinical materials.

7.1.1. Packaging and Labeling

LY3454738 will be supplied to the investigative sites by Lilly or its designee. Clinical trial materials will be labeled according to the country's regulatory requirements. All IPs will be stored, inventoried, reconciled, and destroyed according to applicable regulations. Clinical trial materials are manufactured in accordance with current good manufacturing practices.

LY3454738 will be supplied for clinical trial use as lyophilized powder in a glass vial. The vial is manufactured to deliver 75 mg of LY3454738. Vials will be supplied in cartons, with the appropriate quantity specific to the planned dispensing schedule of the IP.

Placebo for all cohorts is 0.9% sodium chloride (sterile saline).

When prepared for dosing according to instructions, it will not be possible to distinguish between LY3454738 and placebo.

The IP must be prepared by an unblinded pharmacist who is not involved in any other study-related procedures.

7.2. Method of Treatment Assignment

Randomization tables for allocation of LY3454738 or placebo will be prepared by the statistician or designee for the study and provided to the site pharmacists involved in dose preparation; an interactive web response system (IWRS) will be utilized for study Part C. The allocation and dispensation of the IP will be fully documented and verified by a second person. Detailed records of the amounts of the IP received, dispensed, and remained at the end of the study will be maintained by the site pharmacy.

7.2.1. Selection and Timing of Doses

Refer to Section 5.5 for dosing details.

Participants will be asked not to eat breakfast prior to arriving at the clinical site for dosing. The actual time of all dose administrations will be recorded in the participant’s electronic case report form (eCRF).

7.3. Blinding

Blinding will be maintained throughout the conduct of the study as described in the separate Unblinding Plan.

For Parts and A and B of the study, emergency codes will be available to the investigator. A code that reveals the treatment group for a specific study participant may be opened during the study only if the participant’s well-being requires knowledge of the participant’s treatment assignment.

For Part C of the study, emergency unblinding for AEs may be performed through the IWRS, which may supplement or take the place of emergency codes generated by a computer drug-labeling system. This option may be used ONLY if the participant’s well-being requires knowledge of the participant’s treatment assignment. All actions resulting in an unblinding event are recorded and reported by the IWRS.

If a participant’s study treatment assignment is unblinded, the participant must be discontinued from the study, unless the investigator obtains specific approval from a Lilly clinical pharmacologist or clinical research physician (CRP) for the study participant to continue in the study. During the study, emergency unblinding should occur only by accessing the study participant’s emergency code.

In case of an emergency, the investigator has the sole responsibility for determining if unblinding of a participant’s treatment assignment is warranted for medical management of the event. The

participant's safety must always be the first consideration in making such a determination. If the investigator decides that unblinding is warranted, it is the responsibility of the investigator to promptly document the decision and rationale and notify Lilly as soon as possible.

Upon completion of the study, all codes must be returned to Lilly or its designee.

7.4. Dose Modification

7.4.1. Dose Escalation (Part A)

Safety data will be the primary criteria for the dose escalation. In addition, exposure, PK (C_{max} and area under the concentration versus time curve [AUC]) and RO results will be used as supporting data. Dose escalation during the Part A will occur as follows:

- Completion of dosing for all planned participants in the current dosing period
- Review of safety/tolerability data through 2 weeks after dosing (ie, Day 15), including laboratory data through Week 1, for at least 6 participants in the current dosing period.
- Assessment of the LY3454738 concentrations through 1 week postdose in the "one dose removed" prior cohort (ie, Cohort 1 exposure data will be reviewed prior to dosing of Cohort 3, Cohort 2 exposure data will be reviewed prior to dosing of Cohort 4, etc.). All other available exposure data will also be considered.
- Cohort 4b (100 mg LY3454738 SC) is not considered part of the dose escalation and can run in parallel with Cohort 4a (100 mg LY3454738 IV).

A data review is scheduled to occur after each cohort, prior to dose escalation, in order to review emerging safety, tolerability, PK, and target engagement data. This review is described in further detail in Section 10.3.7.

Prior to dose escalation, a blinded safety review meeting will be undertaken by the sponsor and the investigator to evaluate the safety data and agree on the appropriate dose for the next cohort. No dose decision can occur without prior discussion and agreement between the investigator and the Lilly clinical pharmacologist or CRP. If considered appropriate, previous dose levels may be repeated, or lower/intermediate dose levels may be tested. The magnitude of the dose escalation may be reduced after data review, but subsequent escalations from a reduced dose cannot be increased by more than the previously planned dose increment specified in the originally planned dose schedule.

If any of the following scenarios occur, dosing at the current level and further dose escalation will be discontinued:

A single participant experiences a serious adverse event (SAE) or clinically significant event that is related to LY3454738 administration

OR

Two or more participants develop AEs within 14 days of dosing that are considered to be related to study treatment and graded as at least moderate, clinically significant, and not responsive to supportive care

OR

One or more participants develop AEs within 14 days of dosing that are considered to be related to study treatment and graded as severe

OR

Two or more participants develop AEs that are graded as severe, unless there is an obvious explanation other than IP or study procedures for the event(s)

OR

Two or more participants develop (according to Common Terminology Criteria for Adverse Events [CTCAE]) ≥ Grade 2 acute AEs related to the infusion, during or within 2 hours of completing the infusion that do not resolve with a reduced infusion rate and/or supportive care.

7.4.2. Dose Decision (Part B)

The decision to initiate the first participant in Part B will be based on:

1. Completion of dosing in Cohorts 1-5 of Part A and review of safety/tolerability data through 2 weeks after dosing (ie, Day 15), including laboratory data through Week 1, for at least 6 participants in the Cohort 5, along with the cumulative laboratory data from prior cohorts.
2. Assessment of the LY3454738 concentrations through 1 week postdose (Day 8) in Cohorts 1 through 5 of Part A, and any additional available concentration data.
3. Results from the data review (Section 10.3.7).

The planned dose for Part B, desired to achieve a submaximal exposure in order to assess any acute safety/tolerability events associated with a second exposure, is 200 mg. This dose may be modified up or down based on the results from the data review (Section 10.3.7); however, the final dose selected will not exceed one with predicted exposure already assessed and deemed safe and tolerable in Part A.

7.4.3. Dose Decision (Part C)

Part C will be initiated after completion of dosing in Part A and Part B, review of safety and tolerability data from at least 6 participants in Part B through Day 29 (15 days following the second dose), including laboratory, PK, and exposure data through Day 8 following the second dose from at least 6 participants in Part B (additional data reviews scheduled to occur during the study are described in Section 10.3.7).

The planned dose for Part C, desired to achieve a steady state exposure as the maximum exposure achieved after a single dose, is 500 mg. This dose may be modified up or down based on the results from the data review; however, the final dose selected will not exceed one with predicted exposure already assessed and deemed safe and tolerable in Part A.

7.4.4. Special Treatment Considerations

7.4.4.1. Premedication for Infusions

Premedication for the infusions is not planned. However, if an infusion reaction occurs, appropriate medication may be used as determined by the study investigator(s). If infusion reactions are observed, but review of the data suggests that dose escalation may continue, administration of acetaminophen 500 to 1000 mg and/or an antihistamine may be administered orally 30 to 60 minutes prior to the start of infusion for subsequent participants.

The decision to implement premedication for infusions in subsequent cohorts will be made by the investigator and sponsor and recorded in the study documentation, along with the dose-escalation decision.

Any premedications given will be documented as a concomitant therapy (see Section 7.7).

7.4.4.2. Management of Infusion Reactions

There is a risk of infusion reaction with any biological agent; therefore, all participants should be monitored closely. Symptoms and signs that may occur as part of an infusion reaction include, but are not limited to, fever, chills, nausea, headache, bronchospasm, hypotension, angioedema, throat irritation, rash, pruritus, myalgia, and dizziness. In the event that a significant infusion reaction occurs, the following guidance should be followed:

- The IP infusion should be slowed (for example, reduce infusion rate by 50% [eg, an infusion rate of 12 mL/h becomes 6 mL/h or slower]) or stopped, depending on the symptoms/signs present:
  - if slowed, the infusion should be completed at the slower rate, as tolerated
  - if determined by the investigator that the infusion should no longer continue, no further attempts to dose the participant should be made
- Supportive care should be employed in accordance with the symptoms/signs
- If a participant's infusion reaction is sufficiently severe to discontinue the infusion, subsequent infusions may be administered with premedication at the discretion of the investigator following agreement with the Lilly CRP or clinical pharmacologist
- If a participant's infusion rate is reduced due to an infusion reaction, subsequent infusions may be administered at the discretion of the investigator following agreement with the Lilly CRP or clinical pharmacologist. If further infusions are administered, the infusion rate must not exceed the slowest rate used to complete the infusion on the occasion the infusion reaction occurred. Premedication may be administered at the discretion of the investigator
- If it is determined the participant should not receive further doses of IP, the participant should complete AE and other follow-up procedures per the Schedule of Activities (Section 2).

7.5. Preparation/Handling/Storage/Accountability

Follow storage and handling instructions on the IP packaging. Detailed instructions for the preparation and handling of LY3454738 will be provided by the sponsor.

The investigator or designee must confirm appropriate temperature conditions have been maintained, as communicated by sponsor, during transit for all IP received and any discrepancies are reported and resolved before use of the study treatment.

Only participants enrolled in the study may receive IP or study materials, and only authorized site staff may supply or administer IP. All IP should be stored in an environmentally controlled and monitored (manual or automated) area in accordance with the labeled storage conditions with access limited to the investigator and authorized site staff.

The investigator, institution, or the head of the medical institution (where applicable) is responsible for study treatment accountability, reconciliation, and record maintenance (such as receipt, reconciliation, and final disposition records).

7.6. Treatment Compliance

The IP will be administered at the clinical site and documentation of treatment administration will occur at the site.

7.7. Concomitant Therapy

All concomitant medication used at baseline and/or during the course of the trial, whether prescription or over-the-counter, must be recorded on the Concomitant Medication eCRF. Participants will be instructed to consult the investigator or other appropriate study personnel at the clinical site before taking any new medications or supplements during the study.

For AD therapies permitted as part of rescue therapy, see below:

Criteria for rescue therapy initiation

- Investigators should attempt to manage participants with emollients; however, investigators are allowed to rescue participants who are experiencing unacceptable or worsening symptoms of AD at Week 8. Prior to rescue, it is recommended that increased frequency of emollient use is attempted to at least twice a day or more in an effort to control symptoms. The rationale for rescue will be documented.

Choice of rescue therapy treatment

- Triamcinolone 0.1% cream and/or hydrocortisone 2.5% ointment. In the event where providing 1 or both of these topical formulations is not possible, an alternate, equivalent potency TCS cream and/or ointment may be provided.
    - Commercially available triamcinolone 0.1% cream and/or hydrocortisone 2.5% ointment may be supplied by the clinical site. Where providing triamcinolone 0.1% cream and/or hydrocortisone 2.5% ointment is not possible, an equivalent potency TCS cream and/or ointment that is in line with local practices can be supplied.

    - TCS cream and/or ointment that is of appropriate potency and in line with local practices can be supplied by the clinical site.
- Investigators may elect to use TCNIs and/or crisaborole where approved, although use of either during the study is not encouraged. If TCNIs are prescribed, use should be limited to problem areas only (eg, face, neck, skin folds, and genital areas).
- On the days of study visits, topical therapy should not be applied before the participant has undergone all study procedures and clinical evaluations in order to allow adequate assessment of skin dryness.
- Participants receiving topical therapy will remain on study and continue to take IP. Use of rescue therapy will be documented in the eCRF.

In participants who do not improve sufficiently with the provided rescue topical therapy after 7 days, a higher potency TCS may be used and IP may continue. It is recommended that if a participant achieves “clear” to “almost clear” skin after topical rescue, then medium- and/or high-potency TCSs and TCNIs should be stopped, and low-potency TCSs (eg, hydrocortisone 2.5% ointment) should be used once daily for an additional 7 days, then stopped. If lesions return, participants can be retreated with TCSs with or without TCNIs and/or crisaborole as before at the discretion of the investigator.

If topical rescue therapy as described above fails to sufficiently control AD symptoms, then oral systemic medications may be used as rescue (eg, corticosteroids, cyclosporine, methotrexate); however, IP will be required to be permanently discontinued for the remainder of the 12-week study duration. If these medications are needed for other medical conditions (eg, asthma flare), they will still be treated as rescue medications.

Investigators should make every attempt to conduct efficacy and safety assessments immediately before administering any rescue treatment. An unscheduled visit can be used for this purpose if necessary.

7.8. Treatment after the End of the Study

LY3454738 will not be available to participants following completion of the study.

8. Discontinuation Criteria

Participants discontinuing from the treatment prematurely for any reason should complete AE and other follow-up procedures per the Schedule of Activities (Section 2).

Participants discontinuing from the study prematurely for any reason must complete AE and follow-up procedures per the Schedule of Activities (Section 2).

8.1. Discontinuation from Study Treatment

8.1.1. Part A (SAD Design in Healthy Participants)

Not applicable for the SAD portion of the study.

8.1.2. Part B and Part C (Repeat-Dose in Healthy Participants and Participants with AD)

Discontinuation of the IP for abnormal liver tests **should be considered** by the investigator when a participant meets 1 of the following conditions after consultation with the Lilly designated medical monitor:

- alanine aminotransferase (ALT) or aspartate aminotransferase (AST) >[5X for healthy participants, 8X for participants with AD] upper limit of normal (ULN)
- ALT or AST >[3X ULN for healthy participants, 5X ULN for participants with AD] sustained for more than 2 weeks (applicable to Part C only, on account of repeat dosing beyond 2 weeks) or
- ALT or AST >3X ULN and total bilirubin level (TBL) >2X ULN or international normalized ratio (INR) >1.5 or
- ALT or AST >3X ULN the appearance of fatigue, nausea, vomiting, right upper-quadrant pain or tenderness, fever, rash, and/or eosinophilia (>5%)
- Alkaline phosphatase (ALP) >3X ULN
- ALP>2.5X ULN and TBL >2X ULN
- ALP>2.5 ULN with the appearance of fatigue, nausea, vomiting, right quadrant pain or tenderness, fever, rash, and/or eosinophilia (>5%).

Any participant who experiences an event that meets the stopping criteria defined in Section 7.4.1 will not receive additional planned doses but will remain in the study for safety follow-up.

In addition, participants will be permanently discontinued from IP if they experience any 1 of the following events on 2 consecutive samples taken at least 48 hours apart:

- Total white blood cell (WBC) count <1000 cells/µL.
- Absolute neutrophil count (ANC) <500 cells/µL.

- Absolute lymphocyte count (ALC) <200 cells/µL.

Criteria for Temporary Interruption (Withholding) of Study Treatment

In the repeat-dose parts of this study (Parts B and C), the following criteria will be used to interrupt and resume dosing of IP in participants who experience reductions ***in WBC counts and are not permanently discontinued per the above criteria.***

- interrupt if total WBC count is <2000 cells/µL (leukopenia) and resume when total WBC count is ≥2000 cells/µL
- interrupt if ANC is <1000 cells/mL (neutropenia) and resume when ANC is ≥1000 cells/µL
- interrupt if ALC is <500 cells/µL (lymphopenia) and resume when ALC is ≥500 cells/µL.

Temporary withholding of study intervention is required for the development of any infections. Study intervention is to be withheld until resolution of all acute clinical signs and symptoms, and completion of all appropriate anti-infective treatment.

8.1.3. Discontinuation of Inadvertently Enrolled Participants

If the sponsor or investigator identifies a participant who did not meet enrollment criteria and was inadvertently enrolled, a discussion must occur between the Lilly clinical pharmacologist or CRP and the investigator to determine if the participant may continue in the study. If both agree that it is medically appropriate to continue, the investigator must obtain documented approval from the Lilly clinical pharmacologist or CRP to allow the inadvertently enrolled participant to continue in the study with or without continued treatment with IP.

8.2. Discontinuation from the Study

Participants will be discontinued in the following circumstances:

- Enrollment in any other clinical study involving an IP or enrollment in any other type of medical research judged not to be scientifically or medically compatible with this study
- Participation in the study needs to be stopped for medical, safety, regulatory, or other reasons consistent with applicable laws, regulations, and good clinical practice (GCP)
- Investigator Decision
  - the investigator decides that the participant should be discontinued from the study
  - if the participant, for any reason, requires treatment with another therapeutic agent that has been demonstrated to be effective for treatment of the study indication, discontinuation from the study occurs prior to introduction of the new agent
- Participant Decision
  - the participant, or legal representative, requests to be withdrawn from the study.

8.3. Participants Lost to Follow-up

A participant will be considered lost to follow-up if he or she repeatedly fails to return for scheduled visits and is unable to be contacted by the study site. Site personnel are expected to make diligent attempts to contact participants who fail to return for a scheduled visit or were otherwise unable to be followed up by the site.

9. Study Assessments and Procedures

Section 2 lists the Schedule of Activities, detailing the study procedures and their timing (including tolerance limits for timing).

Appendix 2 lists the laboratory tests that will be performed for this study.

Appendix 5 provides a summary of the maximum number and volume of invasive samples, for all sampling, during the study.

Unless otherwise stated in subsections below, all samples collected for specified laboratory tests will be destroyed within 60 days of receipt of confirmed test results. Certain samples may be retained for a longer period, if necessary, to comply with applicable laws, regulations, or laboratory certification standards.

9.1. Efficacy Assessments

In Part C of the trial, the following efficacy assessments will be performed:

Clinician-rated Scales:

- **Validated Investigator's Global Assessment for Atopic Dermatitis (vIGA-AD)**: The vIGA-AD measures the investigator's global assessment of the participant's overall severity of their AD, based on a static, numeric 5-point scale from 0 (clear skin) to 4 (severe disease). The score is based on an overall assessment of the degree of erythema, papulation/induration, oozing/crusting, and lichenification. A copy of the vIGA-AD scale is provided in Appendix 6.
- **Eczema Area and Severity Index (EASI):** The EASI assesses 4 clinical signs of disease, each on a 0 to 3 scale: (1) erythema, (2) induration/papulation, (3) excoriation, and (4) lichenification. Scores range from 0 to 72. The EASI evaluates 2 dimensions of AD: disease extent and clinical signs. Body surface area affected by AD will be derived from data collected as part of the EASI assessment.
- **SCORing Atopic Dermatitis (SCORAD):** The SCORAD index assesses: 1) 6 clinical signs of disease, each on a 0 to 3 scale, 2) extent of involved surface area, up to 100%, and 3) subjective evaluations of itch and sleeplessness, each on a 0 to10 scale. Scores range from 0 to 103.

Participant-rated Scales:

- **Dermatology Life Quality Index (DLQI):** The DLQI is a simple, patient-administered, 10-item questionnaire that covers 6 domains including symptoms and feelings, daily activities, leisure, work and school, personal relationships, and treatment. The recall period for this scale is over the "last week." Response categories include "a little," "a lot," and "very much," with corresponding scores of 1, 2, and 3, respectively, and "not at all", or unanswered ("not relevant") responses scored as 0. Scores range from 0 to 30, with higher scores indicating greater impairment of quality of life. A DLQI total score of

0 or 1 is considered to indicate no effect on a participant's health-related quality of life (Hongbo et al. 2005), and a 4-point change from baseline is considered the minimal clinically important difference threshold (Basra et al. 2015).

- **Patient Oriented Eczema Measure (POEM):** The POEM assesses the frequency of 7 symptoms (itching, sleep disturbance, bleeding, weeping/oozing, cracking, flaking, and dryness/roughness) over the last week. Response categories include "No days," "1-2 days," "3-4 days," "5-6 days," and "Every day," with corresponding scores of 0, 1, 2, 3, and 4, respectively. Scores range from 0 to 28, with higher total scores indicating greater disease severity.

9.2. Adverse Events

Investigators are responsible for monitoring the safety of participants who have entered this trial and for alerting Lilly to any event that seems unusual, even if this event may be considered an unanticipated benefit to the participant. The investigator is responsible for the appropriate medical care of participants during the study.

Investigators must document their review of each laboratory safety report.

The investigator remains responsible for following, through an appropriate health care option, AEs that are serious or otherwise medically important, considered related to the IP or the study, or that caused the participant to discontinue the IP before completing the study. The participant should be followed until the event resolves, stabilizes with appropriate diagnostic evaluation, or is reasonably explained. The frequency of follow-up evaluations of the AE is left to the discretion of the investigator.

Lack of drug effect is not an AE in clinical studies, because the purpose of the clinical study is to establish treatment effect. Cases of pregnancy that occur during maternal or paternal exposures to the IP should be reported. Data on fetal outcome and breast-feeding are collected for regulatory reporting and drug safety evaluation.

After the ICF is signed, study site personnel will record, via eCRF, the occurrence and nature of each participant's preexisting conditions, including clinically significant signs and symptoms of the disease under treatment in the study. Additionally, study site personnel will record any change in the condition(s) and the occurrence and nature of any AEs. Investigators should record their assessment of the potential relatedness of each AE to IP, via eCRF.

The investigator will interpret and document whether or not an AE has a reasonable possibility of being related to study treatment or a study procedure, taking into account the disease, concomitant treatment, or pathologies. A "reasonable possibility" means that there is a cause-and-effect relationship between the IP, study device, and/or study procedure and the AE. The investigator answers yes/no when making this assessment.

Planned surgeries and nonsurgical interventions should not be reported as AEs unless the underlying medical condition has worsened during the course of the study.

If a participant's IP is discontinued as a result of an AE, study site personnel must report this to Lilly or its designee via eCRF, clarifying if possible the circumstances leading to any dosage modifications, or discontinuations of treatment.

9.2.1. Serious Adverse Events

An SAE is any AE from this study that results in 1 of the following outcomes:

- death
- initial or prolonged inpatient hospitalization
- a life-threatening experience (ie, immediate risk of dying)
- persistent or significant disability/incapacity
- congenital anomaly/birth defect
- important medical events that may not be immediately life-threatening or result in death or hospitalization but may jeopardize the participant or may require intervention to prevent 1 of the other outcomes listed in the definition above. Examples of such medical events include allergic bronchospasm requiring intensive treatment in an emergency room or at home, blood dyscrasias or convulsions that do not result in inpatient hospitalization, or the development of drug dependency or drug abuse.

All AEs occurring after signing the ICF are recorded in the eCRF and assessed for serious criteria. The SAE reporting to the sponsor begins after the participant has signed the ICF and has received IP. However, if an SAE occurs after signing the ICF, but prior to receiving IP, the SAE should be reported to the sponsor as per SAE reporting requirements and timelines if it is considered reasonably possibly related to study procedure.

Study site personnel must alert the Lilly CRP, clinical pharmacologist, or its designee, of any SAE as soon as practically possible.

Additionally, study site personnel must alert Lilly Global Patient Safety, or its designee, of any SAE within 24 hours of investigator awareness of the event via a sponsor-approved method. If alerts are issued via telephone, they are to be immediately followed with official notification on study-specific SAE forms. This 24-hour notification requirement refers to the initial SAE information and all follow-up SAE information. Participants with a serious hepatic AE should have additional data collected using the hepatic safety eCRF.

Pregnancy (during maternal or paternal exposure to IP) does not meet the definition of an AE. However, to fulfill regulatory requirements, any pregnancy should be reported following the SAE process to collect data on the outcome for both mother and fetus.

Investigators are not obligated to actively seek AEs or SAEs in participants once they have discontinued and/or completed the study (the participant summary eCRF has been completed). However, if the investigator learns of any SAE, including a death, at any time after a participant

has been discharged from the study, and he/she considers the event reasonably possibly related to the study treatment or study participation, the investigator must promptly notify Lilly.

9.2.1.1. Suspected Unexpected Serious Adverse Reactions

Suspected unexpected serious adverse reactions (SUSARs) are serious events that are not listed in the IB and that the investigator reports as related to IP or procedure. Lilly has procedures that will be followed for the recording and expedited reporting of SUSARs that are consistent with global regulations and the associated detailed guidances.

9.2.2. Complaint Handling

Lilly collects product complaints on IPs and drug delivery systems used in clinical trials in order to ensure the safety of study participants, monitor quality, and facilitate process and product improvements.

Participants should be instructed to contact the investigator as soon as possible if he or she has a complaint or problem with the IPs so that the situation can be assessed.

9.3. Treatment of Overdose

For the purposes of this study, an overdose of LY3454738 is considered any dose higher than the dose assigned through randomization.

The treatment for overdose is supportive care (refer to the IB for LY3454738).

9.4. Safety

9.4.1. Laboratory Tests

For each participant, laboratory tests detailed in Appendix 2 should be conducted according to the Schedule of Activities (Section 2).

With the exception of safety laboratory test results that may unblind the study, Lilly or its designee will provide the investigator with the results of laboratory tests analyzed by a central vendor, if a central vendor is used for the study.

9.4.2. Vital Signs

Vital signs will be assessed as specified in the Schedule of Activities (Section 2) and as clinically indicated. Additional vital signs may be measured during each study period as per judgment of the investigator. Blood pressure and pulse rate should be measured after at least 5 minutes supine.

Orthostatic vital signs should be assessed, if possible, during any AE of dizziness or posture-induced symptoms. If orthostatic measurements are required, participants should be supine for at least 5 minutes and stand for at least 3 minutes. If the participant feels unable to stand, supine vital signs only will be recorded.

9.4.3. Electrocardiograms

For each participant, 12-lead digital ECGs will be collected according to the Schedule of Activities (Section 2). Electrocardiograms should be recorded before collecting any blood for safety or PK tests. Participants must be supine for approximately 5 to 10 minutes before ECG collection and remain supine but awake during ECG collection.

Electrocardiograms are requested to be taken at the specified time; however, aberrations to specified recording times will not be considered protocol deviations as long as the ECGs are taken and the actual recording time is documented. Electrocardiograms may be obtained at additional times when deemed clinically necessary. Collection of additional ECGs at a particular time point is allowed to ensure high quality records. Electrocardiograms will be interpreted by a qualified physician (the investigator or qualified designee) at the study site as soon after the time of ECG collection as possible, and ideally while the participant is still present, to determine whether the participant meets entry criteria at the relevant visit(s) and for immediate participant management, should any clinically relevant findings be identified.

If a clinically significant finding is identified (including, but not limited to changes in QT/corrected QT interval [QTc] from baseline) after enrollment, the investigator in conjunction with the sponsor will determine if the participant can continue in the study. The investigator or qualified designee is responsible for determining if any change in participant management is needed and must document his/her review of the ECG printed at the time of collection. If clinically significant changes are observed, triplicate ECGs may be collected (at approximately 1-minute intervals) for this participant and/or other participants and at least 1 of the replicate ECGs from each time point will be reviewed. Any new clinically relevant finding should be reported as an AE.

A central ECG laboratory will perform a basic quality control check (eg, demographics and study details) and will then store the ECGs in a database. At a future time, the stored ECG data may be overread at the central ECG laboratory for further evaluation of machine-read measurements or to meet regulatory requirements.

The machine-read ECG intervals and heart rate may be used for data analysis and report writing purposes unless a cardiologist over-read of the ECGs is conducted prior to completion of the final study report (in which case the over-read data would be used).

9.4.4. Physical Examination

Physical examinations and routine medical assessments (including body weight and height measurements) will be conducted as specified in the Schedule of Activities (Section 2) and as clinically indicated.

Height will only be recorded once, at screening. The participant should not wear shoes during measurement.

9.4.5. Injection Site Assessments

If the investigator determines that any injection site reaction (ISR) is clinically significant or if a participant indicates symptoms are indicative of an ISR (unsolicited event; volunteered by participant), the event will be captured as an AE. If an ISR is deemed to be an AE, an ISR form will be used to capture specific information about this reaction (eg, degree and area of erythema, presence of induration or specific details about the pain experienced).

If there is more than 1 symptom of an injection site reaction that meets the definition of an AE, a single AE of injection site reaction will be recorded on the AE page of the eCRF. If 1 or more symptom(s) of an injection-/infusion-site reaction is reported during the assessment, a single AE for injection or infusion-site reaction will be recorded on the AE page of the eCRF.

9.4.6. Safety Monitoring

The Lilly clinical pharmacologist and/or CRP will monitor safety data throughout the course of the study.

Lilly will review SAEs within time frames mandated by company procedures. The Lilly clinical pharmacologist or CRP will consult with the functionally independent Global Patient Safety medical physician or clinical research scientist when appropriate, and periodically review:

- trends in safety data
- laboratory analytes including flow cytometry data
- AEs

9.4.6.1. Hepatic Safety

If a study participant experiences elevated ALT ≥3X ULN, ALP ≥2X ULN, or elevated total bilirubin ≥2X ULN, liver tests (Appendix 4) should be repeated within 3 to 5 days including ALT, AST, ALP, TBL, direct bilirubin, gamma-glutamyl transferase (GGT), and creatinine kinase to confirm the abnormality and to determine if it is increasing or decreasing. If the abnormality persists or worsens, clinical and laboratory monitoring should be initiated by the investigator based on consultation with the Lilly clinical pharmacologist or CRP. Monitoring should continue until levels normalize and/or are returning to approximate baseline levels.

Additional safety data should be collected if 1 or more of the following conditions occur:

- elevation of serum ALT to ≥ 5X ULN on 2 or more consecutive blood tests
- elevated serum TBL to ≥ 2X ULN (except for cases of known Gilbert's syndrome)
- elevation of serum ALP to ≥ 2X ULN on 2 or more consecutive blood tests
- participant discontinued from treatment due to a hepatic event or abnormality of liver tests
- hepatic event considered to be a SAE.

9.5. Pharmacokinetics

At the visits and times specified in the Schedule of Activities (Section 2), venous blood samples will be collected to determine the serum concentrations of LY3454738 (Appendix 5). The proposed sampling schedule may be modified during the study based on the results from the data review PK data snapshots. In particular, sampling duration may be extended if required. In addition, up to 3 additional blood samples may be collected during the study if warranted and agreed on between both the investigator and sponsor. In case of early termination, a PK sample will be taken at the early discontinuation (ED) visit. Drug concentration information that would unblind the study will not be reported to investigative sites or blinded personnel.

Blood samples are requested to be taken at the specified time. However, deviations from the specified sampling times will not be considered protocol deviations as long as the samples are taken and the actual sampling time is recorded. It is essential that the actual times of doses and samples are recorded accurately on the appropriate forms. Instructions for the collection and handling of blood samples will be provided by the sponsor.

When there is a scheduling conflict between PK sample collection and other study activities, PK samples are to take priority over all other study activities, except for safety interventions in the case of an AE. The actual time of PK sample collection should be recorded to the nearest minute, as close as possible to the times shown in the proposed schedule. The timing of ECG and vital sign recording (and other study activities) can be altered slightly, if necessary to accommodate PK sampling.

Drug concentration information that may unblind the study will not be reported to investigative sites or blinded personnel until the study has been unblinded.

9.5.1. Bioanalysis

Samples will be analyzed at a laboratory approved by the sponsor and stored at a facility designated by the sponsor.

Concentrations of LY3454738 will be assayed using a validated enzyme-linked immunosorbent assay (ELISA) method. Analyses of samples collected from placebo-treated participants are not planned.

Bioanalytical samples collected to measure IP concentrations will be retained for a maximum of 1 year following last participant visit for the study.

The remaining sample collected for PK testing may be pooled and used for exploratory metabolism or exploratory analyses such as bioanalytical assay validation or cross-validation exercise.

9.6. Pharmacodynamics

9.6.1. Receptor Occupancy

At the times specified in the Schedule of Activities (Section 2) venous blood samples will be collected and used to determine the target engagement (RO) effects of LY3454738. Blood will be collected in order to evaluate RO activity.

Supplies required for the collection and shipment of the participants' samples will be supplied by the sponsor. Sample handling and shipment to the central laboratory will occur per instructions given to the study site. Any unused blood samples will be destroyed in accordance with local regulations.

9.6.2. Immunogenicity Assessments

At the visits and times specified in the Schedule of Activities (Section 2), venous blood samples will be collected to determine antibody production against LY3454738. To interpret the results of immunogenicity, blood samples will be collected at the same time points to determine the serum concentrations of LY3454738. All samples for immunogenicity should be taken predose when applicable and possible. In the event of an infusion-related reaction (IRR), blood samples will be collected for PK, immunogenicity, and exploratory hypersensitivity analyses at the following time points, as close as possible to: (i) the onset of the IRR, (ii) the resolution of the IRR, and (iii) 30 [±3] days following the IRR. Exploratory hypersensitivity samples may be analyzed for markers of basophil/mast cell activation (eg, tryptase), immune complex formation (eg, C3 levels) and cytokine release (eg, IL-6) as appropriate for the clinical presentation.

Instructions for the collection and handling of blood samples will be provided by the sponsor. The actual date and time (24-hour clock time) of each sampling will be recorded.

Treatment-emergent antidrug antibodies (ADAs) are defined in Section 10.3.6.

Immunogenicity will be assessed by a validated assay designed to detect ADAs in the presence of the LY3454738 at a laboratory approved by the sponsor. Samples will be stored at -70°C and tests for immunogenicity will be run as batches. Antibodies may be further characterized and/or evaluated for their ability to neutralize the activity of LY3454738. In the event of any AE suspected of being related to immunogenicity, the presence of ADAs may also be evaluated.

Samples may be stored for a maximum of 15 years following last participant visit for the trial at a facility selected by the sponsor to enable further analysis of immune responses to LY3454738. The duration of storage allows the sponsor to respond to regulatory requests related to LY3454738. Any samples remaining after 15 years will be destroyed.

9.7. Genetics

A blood sample will be collected for pharmacogenetic analysis as specified in the Schedule of Activities (Section 2), where local regulations allow.

Samples will not be used to conduct unspecified disease or population genetic research either now or in the future. Samples will be used to investigate variable exposure or response to

LY3454738 and to investigate genetic variants thought to play a role in AD. Assessment of variable response may include evaluation of AEs or differences in efficacy.

All samples will be coded with the participant number. These samples and any data generated can be linked back to the participant only by the investigative site personnel.

Samples will be retained for a maximum of 15 years after the last participant visit, or for a shorter period if local regulations and/or institutional review boards (IRBs) impose shorter time limits, for the study at a facility selected by Lilly or its designee. This retention period enables use of new technologies, response to regulatory questions, and investigation of variable response that may not be observed until later in the development of LY3454738 or after LY3454738 is commercially available.

Molecular technologies are expected to improve during the 15-year storage period and therefore cannot be specifically named. However, existing approaches include whole genome or exome sequencing, genome wide association studies, multiplex assays, and candidate gene studies. Regardless of technology utilized, data generated will be used only for the specific research scope described in this section.

9.8. Biomarkers

Biomarker research is performed to address questions of relevance to drug disposition, target engagement, pharmacodynamics, mechanism of action, variability of participant response (including safety), and clinical outcome. Sample collection is incorporated into clinical studies to enable examination of these questions through measurement of biomolecules including deoxyribonucleic acid (DNA), ribonucleic acid (RNA), proteins, lipids, and other cellular elements.

Serum, plasma, whole blood RNA, and samples for non-pharmacogenetic biomarker research will be collected at the times specified in the Schedule of Activities (Section 2) where local regulations allow.

Samples will be used for research on the drug target, disease process, variable response to LY3454738 pathways associated with AD (Part C), mechanism of action of LY3454738, and/or research method, or for validating diagnostic tools or assay(s) related to AD (Part C).

Target engagement will be assessed using a whole blood assay measuring the binding of LY3454738 to CD200R expressed on neutrophils by flow cytometry. Receptor occupancy will then be calculated for each sample at each time point tested and graphed to demonstrate binding over time. Separate samples for the expressed purpose of evaluating serum IL-19 levels will be collected in Part C.

All samples will be coded with the participant number (coded). These samples and any data generated can be linked back to the participant number (coded) only by the investigative site personnel.

Samples will be retained for a maximum of 15 years after the last participant visit, or for a shorter period if local regulations and/or IRB impose shorter time limits, at a facility selected by

Lilly or its designee. This retention period enables use of new technologies, response to regulatory questions, and investigation of variable response that may not be observed until later in the development of LY3454738 or after LY3454738 or is commercially available.

9.8.1. Skin Biopsies for Participants with AD

All participants in Part C will be required to have skin biopsies, which will be collected at the times shown in the Schedule of Activities (Section 2). Detailed instructions for handling the biopsies at the study site will be provided by the sponsor. These biopsies will be analyzed in relation to the changes in disease activity measures within the same participant with AD.

Biopsies will be retained for a maximum of 15 years after the last participant visit, or for a shorter period if local regulations and ethical review boards (ERBs) allow, at a facility selected by the sponsor. The duration allows the sponsor to respond to future regulatory requests related to the IP. Any samples remaining after 15 years will be destroyed.

9.9. Health Economics

This section is not applicable for this study.

10. Statistical Considerations and Data Analysis

10.1. Sample Size Determination

The sample sizes in Part A (54 participants) and Part B (8 participants) are not based on statistical calculations, as these cohorts are designed primarily to seek information on safety, tolerability, PK, and target engagement. The sample sizes are not adjusted to account for drop-outs. However, the protocol allows replacement if it is deemed necessary to obtain sufficient data for interpretation.

For Part C, assuming 50% of participants receiving LY3454738 and 8% of participants receiving placebo achieve a vIGA-AD 0/1, a sample size of 30 completers will provide approximately 70% power using a 2-sided Fisher's exact test at the 0.10 significance level.

Participants who are randomized but not administered treatment may be replaced to ensure that approximately enough participants may complete the study.

10.2. Populations for Analyses

For purposes of analysis, the following populations are defined:

Population	Description
Enrolled	All participants who sign informed consent
Intent-to-Treat (ITT)	All randomized participants, even if the participant does not take the assigned treatment, does not receive the correct treatment, or otherwise does not follow the protocol

10.2.1. Study Participant Disposition

A detailed description of participant disposition will be provided at the end of the study.

All participants who discontinue from the study will be identified, and the extent of their participation in the study will be reported. If known, a reason for their discontinuation will be given.

10.2.2. Study Participant Characteristics

The participant's baseline characteristics and other demographic characteristics will be recorded, listed, and summarized by treatment group and overall.

10.3. Statistical Analyses

Statistical analysis of this study will be the responsibility of Lilly or its designee. Summary statistics, data tabulations, and data graphs by population (overall, Japanese, and non-Japanese) will be provided, as appropriate.

Pharmacodynamic analyses will be conducted on data from all participants who receive at least 1 dose of the IP.

Pharmacokinetic analyses will be conducted on data from all participants who receive at least 1 dose of the IP and have evaluable PK. Clinical activity and PD analyses will be conducted for all participants with AD who receive the IP (Part C only).

Safety analyses will be conducted for all enrolled participants, whether or not they completed all protocol requirements.

For Part C, efficacy analyses will be conducted on the ITT population. This set includes all data from all randomized participants receiving at least 1 dose of the IP according to the treatment the participants were assigned.

All protocol deviations that occur during the study will be considered for their severity/impact and will be taken into consideration when participants are assigned to analysis populations prior to database lock and unblinding. Details of participant assignment to the analysis populations will be listed.

For Part A and Part B, the placebo data will be pooled across all dose-escalation cohorts for the purpose of analysis.

Additional exploratory analyses of the data will be conducted as deemed appropriate. Study results may be pooled with the results of other studies for population PK analysis purposes to avoid issues with post hoc analyses and incomplete disclosures of analyses.

10.3.1. Safety Analyses

10.3.1.1. Clinical Evaluation of Safety

All IP and protocol procedure AEs will be listed and, if the frequency of events allows, safety data will be summarized using descriptive methodology.

The incidence of symptoms for each treatment will be presented by severity and by association with IP as perceived by the investigator. Symptoms reported to occur prior to treatment with IP will be distinguished from those reported as new or increased in severity during the study. All infusion and injection site reactions, related treatment-emergent adverse event (TEAE) terms, and severity grades will be assigned by the investigator using the CTCAE. All other AE terms will be graded as mild, moderate, or severe by the investigator, and classified by the most suitable term from the medical regulatory dictionary.

A TEAE is defined as an event that emerges after treatment (ie, after administration of placebo or LY3454738), having been absent pretreatment, or worsens relative to the pretreatment state. The number of participants who experience a TEAE and/or SAE (all causalities and related to IP) will be summarized by study treatment. All TEAEs will be summarized by system organ class and by decreasing frequency within system organ class. Infusion and injection site reactions will be summarized by maximum CTCAE grade.

The number of IP-related SAEs will be reported.

10.3.1.2. Statistical Evaluation of Safety

Safety parameters that will be assessed include safety laboratory parameters, vital signs, and ECG parameters. The parameters will be listed and summarized using standard descriptive statistics. Additional analysis will be performed if warranted upon review of the data.

Baseline for safety parameters will be defined as the last evaluable value before the first dose.

10.3.2. Efficacy Analyses

All efficacy analyses will be performed for Part C of the study only.

Fisher's exact test will be used for treatment comparisons of discrete efficacy variables. The percentages and difference in percentages will be reported. Continuous efficacy variables will be analyzed by an analysis of covariance (ANCOVA) with treatment and baseline value in the model. Descriptive statistics will be reported.

Participants who receive rescue treatment will be considered treatment failures at all subsequent time points.

10.3.2.1. Primary Analyses

The primary efficacy measure is the binary outcome of response defined as a vIGA-AD score of 0 or 1 (clear or almost clear skin) with a ≥ 2 point improvement from baseline at Week 12. The primary analysis will be conducted using Fisher's exact test.

10.3.2.2. Secondary Analyses

Fisher's exact test will be used to analyze the proportion of participants achieving

- vIGA-AD of 0 or 1 with a ≥ 2 point improvement from baseline at Week 1 through Week 12and/or early discontinuation (as specified in the schedule of assessments [Section 2]).
- EASI50, EASI75, and EASI90 at Week 1 through Week 12 and or/early discontinuation (as specified in the schedule of assessments [Section 2]), where EASI50, EASI75, and EASI90 are defined as having an improvement of at least 50%, 75%, and 90% from baseline, respectively.
- SCORAD 50, SCORAD75 and SCORAD90 at Week 1 through Week 12 and or/early discontinuation (as specified in the schedule of assessments [Section 2]), where SCORAD75 and SCORAD90 are defined as having an improvement of at least 75% and 90% from baseline, respectively.

Mean change from baseline in EASI and SCORAD will be assessed at Week 1 through Week 12 and or/early discontinuation (as specified in the schedule of assessments [Section 2]) using the previously described model for continuous endpoints.

10.3.2.3. Exploratory Analyses

Improvement in signs and symptoms, health outcome measures, and quality of life measures (total scores, item scores, and derivations) will be summarized using descriptive statistics and the previously described models for discrete and continuous endpoints.

10.3.3. Pharmacokinetic Analyses

10.3.3.1. Pharmacokinetic Parameter Estimation

Pharmacokinetic parameter estimates for LY3454738 will be calculated by standard noncompartmental methods of analysis and summarized by dose and route of administration using descriptive statistics.

The primary parameters for analysis will be C_{max}, and AUC of LY3454738 after single- and multiple-dose administration. Other noncompartmental parameters such as time to maximum drug concentration (T_{max}), half-life, apparent clearance, apparent volume of distribution, and SC bioavailability may be reported. Analyses of other PK parameters may be performed, if deemed appropriate. Population analysis of the concentration-time data based on a compartmental model may also be performed to support PK simulations. Subgroup comparisons of Japanese versus non-Japanese participants may be performed using graphical and tabular comparisons.

10.3.3.2. Pharmacokinetic Statistical Inference

Pharmacokinetic parameters will be evaluated to estimate dose proportionality based on data in the SAD part of the study. Log-transformed C_{max} and AUC parameters will be evaluated using a power model (where log dose acts as an explanatory variable) to estimate ratios of dose-normalized geometric means and corresponding 90% confidence intervals. The estimated ratio of dose-normalized geometric means of PK parameters between the highest and lowest doses will be used to assess dose proportionality. A subinterval within the highest and lowest doses may also be considered for assessment of dose proportionality using the same approach. For the analysis of the dose proportionality assessment, the data from the placebo group will be excluded. Additional analyses following SC administration may be performed, if possible.

10.3.4. Pharmacodynamic Analyses

10.3.4.1. Pharmacodynamic Parameter Estimation

Pharmacodynamic analysis will be reported as % RO per participant over time based on LY3454738 binding to neutrophils from whole blood.

10.3.5. Pharmacokinetic/Pharmacodynamic Analyses

The relationship between LY3454738 exposure and RO maybe explored with graphical analysis. If the relationship is deemed to exist, a population PK/PD analysis approach may be applied.

The PD parameters for analysis will be maximum receptor occupancy (RO_{max}) and dose or concentration to achieve 50% RO_{max} (ED50 or EC50, respectively). Estimation of other PD parameters may be performed, if deemed appropriate

10.3.6. Evaluation of Immunogenicity

The frequency and percentage of participants with preexisting ADA and with treatment-emergent antidrug antibody positive (TE ADA+) to LY3454738 will be tabulated. Treatment-emergent ADAs are defined as those with a titer 2-fold (1 dilution) greater than the minimum required dilution if no ADAs were detected at baseline (treatment-induced ADA) or those with a

4-fold (2 dilutions) increase in titer compared to baseline if ADAs were detected at baseline (treatment-boosted ADA). For the TE ADA+ participants, the distribution of maximum titers will be described. The frequency of neutralizing antibodies will also be tabulated in TE ADA+ participants.

The relationship between the presence of antibodies and the PK parameters and PD response including safety and efficacy to LY3454738 may be assessed.

10.3.7. Data Review during the Study

Access to the data is scheduled to occur after each cohort prior to dose escalation, and before initiation of the multiple-dose cohorts, in order to review emerging safety, tolerability, drug exposure, and target engagement data. The purpose of these reviews is to guide dose selection for the next dosing session, and distribution of Japanese and non-Japanese participants, in addition to informing the design of subsequent studies.

A data review of safety/tolerability, PK, and target engagement data will occur when at least 6 participants in Cohort 3 have available data at Day 15. The assessment will mainly include data from Cohorts 1, 2, and 3. The data from this data review, along with the additional data detailed in Section 7.4, will be used to confirm or modify the specified dose levels for Cohorts 5 and 6 of Part A and the cohorts of Parts B and C of this study.

The investigator and the Lilly sponsor team will make the determination regarding dose escalation based upon their review of the data. The investigator will remain blinded and the Lilly sponsor team will be unblinded during these reviews. The investigator and the Lilly clinical pharmacologist, Lilly CRP, and Lilly study team will make the determination regarding dose escalation based upon the criteria outlined in Section 7.4.

10.3.8. Interim Analyses

An interim analysis will occur after all planned subjects in Part A and Part B complete Day 29 (Visit 8). The analysis will include a review of safety and tolerability data available at this time; inclusive of laboratory data through, at minimum, Day 29. Any additional data available will also be reviewed.

An unblinded interim analysis for Part C is planned when approximately 30 patients have had the opportunity to complete, at a minimum, the Week 12 visit. If more than 30 patients are required to be enrolled to ensure 30 completers, the Internal Assessment Committee (IAC) may recommend an additional unblinded interim analysis for Part C.

Interim analyses may be conducted using efficacy and/or safety data. The planned and any additional interim analyses are to support subsequent clinical development planning.

Assessments of unblinded interim data, such as efficacy data, will be conducted by an IAC with a limited number of prespecified team members who do not have direct site contact or data entry or data validation responsibilities (see Section 10.3.9). Only the IAC will be authorized to evaluate unblinded interim efficacy and safety analyses. Study sites will receive information about interim results if a safety concern is identified.

Unblinding details will be specified in the unblinding plan section of the statistical analysis plan or in a separate unblinding plan document.

10.3.9. Data Monitoring Committee (DMC)

Not applicable. An IAC will be used to conduct interim analysis of unblinded data. The IAC review will be fully independent from the study team and will include, at a minimum, a Lilly medical physician, a statistician, and a representative from the Lilly Global Patient Safety organization. Details about the IAC membership, purpose, responsibilities, and operation will be described in an IAC charter, which will be approved prior to the first unblinding.

11. References

Basra MK, Salek MS, Camilleri L, Sturkey R, Finlay AY. Determining the minimal clinically important difference and responsiveness of the Dermatology Life Quality Index (DLQI): further data. *Dermatology*. 2015;230(1):27-33.

Blom LH, Martel BC, Larsen LF, Hansen CV, Christensen MP, Juel-Berg N, Mitman T, Poulsen LK. The immunoglobulin superfamily member CD200R identifies cells involved in type 2 immune responses. *Allergy*. 2017;72(7):1081-1090.

Broderick C, Hoek, RM, Forrester JV, Liversidge J, Sedgwick JD, Dick AD. Constitutive retinal CD200 expression regulates resident microglia and activation state of inflammatory cells during experimental autoimmune uveoretinitis. *Am J Pathol.* 2002;161(5):1669-1677.

Chen Z, Yu K, Zhu F, Gorczynski R. Over-expression of CD200 protects mice from dextran sodium sulfate induced colitis. *PloS One*. 2016;11(2):e0146681.

DeGeorge J, Robertson S, Butler L, Derzi M, Stoch SA, Diaz D, Hartke J, Guzzie-Peck P, Mortimer-Cassen E, Bogdanffy M, Will Y, Greene N. An industry perspective on the 2017 EMA guidance on first-in-human and early clinical trials. *Clin Pharmacol Ther.* 2018;103(4):566-569.

Egawa G, Weninger W. Pathogenesis of atopic dermatitis. *Cogent Biology*. 2015;1(1):1103459.

Eichenfield LF, Tom WL, Chamlin SL, Feldman SR, Hanifin JM, Simpson EL, Berger TG, Bergman JN, Cohen DE, Cooper KD, Cordoro KM, Davis DM, Krol A, Margolis DJ, Paller AS, Schwartzenberger K, Silverman RA, Williams HC, Elmets CA, Block J, Harrod CG, Smith Begolka W, Sidbury R. Guidelines of care for the management of atopic dermatitis: section 1. Diagnosis and assessment of atopic dermatitis. *J Am Acad Dermatol.* 2014:70(2):338-351.

[EMA] Europe, the Middle East and Africa. Committee for Medicinal Products for Human Use. Guideline on strategies to identify and mitigate risks for first-in-human and early clinical trials with investigational medicinal products. 2017. Available at: http://www.ema.europa.eu/docs/en_GB/document_library/Scientific_guideline/2017/07/WC500232186.pdf. Published July 20, 2017. Accessed June 22, 2018.

Farré D, Martinez-Vicente P, Engel P, Angulo A. Immunoglobulin superfamily members encoded by viruses and their multiple roles in immune invasion. *Eur J Immunol.* 2017;47(5):780-796.

Hoek RM, Ruuls SR, Murphy CA, Wright GJ, Godard R, Zurawski SM, Blom B, Homola ME, Streit WJ, Brown MH, Barclay AN, Sedgwick JD. Down-regulation of the macrophage lineage through interaction with OX2 (CD200). *Science*. 2000;290(5497);1768-1771.

Hongbo Y, Thomas CL, Harrison MA, Salek MS, Finlay AY. Translating the science of quality of life into practice: What do dermatology life quality index scores mean? *J Invest Dermatol.* 2005;125(4):659-664.

Megna M, Napolitano M, Patruno, C, Villani A, Balato A, Monfrecola G, Ayala F, Balato N. Systemic treatment of adult atopic dermatitis: a review. *Dermatol Ther (Heidelb)*. 2017;7(1):1-23.

Naidoo J, Page DB, Li BT, Connell LC, Schindler K, Lacouture ME, Postow MA, Wolchock JD. Toxicities of the anti-PD-1 and anti-PD-L1 immune checkpoint antibodies [published correction appears in *Ann Oncol*. 2016;27(7):1362]. *Ann Oncol.* 2015;26(12):2375-2391.

Saber H, Gudi R, Manning M, Wearne E, Leighton JK. An FDA oncology analysis of immune activating products and first-in-human dose selection. *Reg Toxicol Pharmacol.* 2016;81:448-456.

Simelyte E, Alzabin S, Boudakov I, Williams R. CD200R1 regulates the severity of arthritis but has minimal impact on the adaptive immune response. *Clin Exp Immunol*. 2010;162(1):163-168.

Weninger W, Biro M, Jain R. Leukocyte migration in the interstitial space of non-lymphoid organs. *Nat Rev Immunol*. 2014:14(4);232-246.

Wright GJ, Cherwinski H, Foster-Cuevas, M, Brooke G, Puklavec MJ, Bigler M, Song Y, Jenmalm M, Gorman D, McClanahan T, Liu MR, Brown, MH, Sedwick JD, Phillips JH, Barclay AN. Characterization of the CD200 receptor family in mice and humans and their interactions with CD200. *J Immunol*. 2003;171(6):3034-3046.

Yu K, Chen Z, Gorczynski R. Effect of CD200 and CD200R1 expression within tissue grafts on increased graft survival in allogenic recipients. *Immunol Lett*. 2013;149(1-2):1-8.

Zhang S, Cherwinski H, Sedgwick JD, Phillips JH. Molecular mechanisms of CD200 inhibition of mast cell activation. *J Immunol*. 2004;173(11):6786-6793.

Appendix 1. Abbreviations and Definitions

Term	Definition
**AD**	atopic dermatitis
**ADA**	antidrug antibody
**ADCC**	antibody-dependent cellular cytotoxicity
**AE**	adverse event: Any untoward medical occurrence in a patient or clinical investigation participant administered a pharmaceutical product that does not necessarily have a causal relationship with this treatment. An AE can therefore be any unfavorable and unintended sign (including an abnormal laboratory finding), symptom, or disease temporally associated with the use of a medicinal (investigational) product, whether or not related to the medicinal (investigational) product.
**ALC**	absolute lymphocyte count
**ALP**	alkaline phosphatase
**ALT**	alanine aminotransferase
**ANC**	absolute neutrophil count
**ANCOVA**	analysis of covariance
**AST**	aspartate aminotransferase
**AUC**	area under the concentration versus time curve
**AUC(0-336)**	area under the concentration versus time curve from time 0 to 336 hours
**blinding**	A procedure in which one or more parties to the study are kept unaware of the treatment assignment(s). Unless otherwise specified, blinding will remain in effect until final database lock.<br>A single-blind study is one in which the investigator and/or his staff (not including the pharmacist) are aware of the treatment but the participant is not, or vice versa, or when the sponsor is aware of the treatment but the investigator and/his staff and the participant are not. A triple-blind study is one in which neither the participant nor any of the investigator or sponsor staff (not including the pharmacist) who are involved in the treatment or clinical evaluation of the participants are aware of the treatment received
**BSA**	body surface area
**C_{ave}**	average drug concentration
**CD200**	CD200R receptor ligand
**CD200R**	CD200 receptor
**C_{max}**	maximum observed drug concentration
**C_{min}**	minimum observed drug concentration

**complaint**	A complaint is any written, electronic, or oral communication that alleges deficiencies related to the identity, quality, purity, durability, reliability, safety or effectiveness, or performance of a drug or drug delivery system.
**Compliance**	Adherence to all the study-related requirements, good clinical practice (GCP) requirements, and the applicable regulatory requirements.
**confirmation**	A process used to confirm that laboratory test results meet the quality requirements defined by the laboratory generating the data and that Lilly is confident that results are accurate. Confirmation will either occur immediately after initial testing or will require that samples be held to be retested at some defined time point, depending on the steps required to obtain confirmed results.
**CRP**	clinical research physician: Individual responsible for the medical conduct of the study. Responsibilities of the CRP may be performed by a physician, clinical research scientist, global safety physician or other medical officer.
**CRU**	Clinical Research Unit
**CTCAE**	Common Terminology Criteria for Adverse Events
**CTLA**	cytotoxic T-lymphocyte associated protein
**DC**	dendritic cell
**DLQI**	Dermatology Life Quality Index
**DNA**	deoxyribonucleic acid
**Dok**	docking protein
**DSS**	dextran sulfate sodium
**EASI**	Eczema Area and Severity Index
**EASI50**	improvement of at least 50% in EASI score from baseline
**EASI75**	improvement of at least 75% in EASI score from baseline
**EASI90**	improvement of at least 90% in EASI score from baseline
**EC50**	concentration to achieve 50% maximum receptor occupancy
**ECG**	electrocardiogram
**EC_{10-15}**	drug concentration that produces 10-15% of the maximum effect
**eCRF**	electronic case report form
**ED**	early discontinuation
**ED50**	dose to achieve 50% maximum receptor occupancy
**ELISA**	enzyme-linked immunosorbent assay

**enroll**	The act of assigning a participant to a treatment. Participants who are enrolled in the study are those who have been assigned to a treatment.
**enter**	Participants entered into a study are those who sign the informed consent form directly or through their legally acceptable representatives.
**ERB**	ethical review board
**GCP**	good clinical practice
**GGT**	gamma-glutamyl transferase
**HBcAb**	hepatitis B core antibody
**HBsAb**	hepatitis B surface antibody
**HIV**	human immunodeficiency virus
**IB**	Investigator's Brochure
**IAC**	Internal Assessment Committee
**ICF**	informed consent form
**Ig**	immunoglobulin
**IgA**	immunoglobulin A
**IgE**	immunoglobulin E
**IgG**	immunoglobulin G
**IgG4**	immunoglobulin G subclass 4
**IgM**	immunoglobulin M
**IL**	interleukin
**ILC**	innate lymphoid cell
**IM**	intramuscular
**informed consent**	A process by which a participant voluntarily confirms his or her willingness to participate in a particular study, after having been informed of all aspects of the study that are relevant to the participant's decision to participate. Informed consent is documented by means of a written, signed and dated informed consent form.
**INR**	international normalized ratio
**interim analysis**	An interim analysis is an analysis of clinical study data, separated into treatment groups, that is conducted before the final reporting database is created/locked.

Investigational product (IP) A pharmaceutical form of an active ingredient or placebo being tested or used as a reference in a clinical study, including products already on the market when used or assembled (formulated or packaged) in a way different from the authorized form, or marketed products used for an unauthorized indication, or marketed products used to gain further information about the authorized form.

investigator A person responsible for the conduct of the clinical study at a study site. If a study is conducted by a team of individuals at a study site, the investigator is the responsible leader of the team and may be called the principal investigator.

IRB institutional review board

IRR infusion-related reaction

ISR injection site reaction

ITIM immunoreceptor tyrosine-based inhibitory motif

ITT intent-to-treat

IV intravenous

IWRS interactive web response system

JAK Janus kinase

Legal Representative An individual or judicial or other body authorized under applicable law to consent, on behalf of a prospective participant, to the participant's participation in the clinical study.

mAb monoclonal antibody

MAPK ras-mitogen activated protein kinase

NOAEL no-observed adverse effect level

NPxY phosphotyrosine binding domain recognition motif

open-label A study in which there are no restrictions on knowledge of treatment allocation, therefore the investigator and the study participant are aware of the drug therapy received during the study.

PD pharmacodynamic(s)

PD-1 programmed cell death protein 1

pDC plasmacytoid dendritic cell

PDE-4 phosphodiesterase type-4

PD-L1 programmed death ligand 1

PK pharmacokinetic(s)

POEM Patient Oriented Eczema Measure

**QTc**	corrected QT interval
**randomize**	the process of assigning participants to an experimental group on a random basis
**RasGAP**	ras GTPase-activating protein 1
**RNA**	ribonucleic acid
**RO**	receptor occupancy
**RO_{max}**	maximum receptor occupancy
**SAD**	single-ascending dose
**SAE**	serious adverse event
**SC**	subcutaneous
**SCORAD**	SCORing Atopic Dermatitis
**SCORAD75**	Improvement of at least 75% in SCORAD score from baseline
**SCORAD90**	Improvement of at least 90% in SCORAD score from baseline
**screen**	The act of determining if an individual meets minimum requirements to become part of a pool of potential candidates for participation in a clinical study.
**SHIP**	SH2-containing inositol 5’ phosphatase
**SUSAR**	suspected unexpected serious adverse reaction
**TB**	tuberculosis
**TBL**	total bilirubin level
**TCNI**	topical calcineurin inhibitor
**TCS**	topical corticosteroid
**TE ADA+**	treatment-emergent antidrug antibody positive
**TEAE**	treatment-emergent adverse event: Any untoward medical occurrence that emerges during a defined treatment period, having been absent pretreatment, or worsens relative to the pretreatment state, and does not necessarily have to have a causal relationship with this treatment
**T_{max}**	time to maximum drug concentration
**TST**	tuberculin skin test
**ULN**	upper limit of normal
**vIGA-AD**	Validated Investigator’s Global Assessment for Atopic Dermatitis

**WBC**	white blood cell

Appendix 2. Clinical Laboratory Tests

**Hematology**	**Clinical Chemistry**
Hematocrit	Sodium
Hemoglobin	Potassium
Erythrocyte count (RBC)	Bicarbonate
Mean cell volume	Chloride
Mean cell hemoglobin	Calcium
Mean cell hemoglobin concentration	Phosphorus
Leukocytes (WBC)	
Differential WBC absolute counts of:	Glucose random
Neutrophils	Blood urea nitrogen (BUN)
Lymphocytes	Uric acid
Monocytes	
Eosinophils	Total protein
Basophils	Albumin
B Cells	
T Cells	
NK cells	
Platelets	Total bilirubin
	Alkaline phosphatase (ALP)
**Urinalysis**	Aspartate aminotransferase (AST)
Specific gravity	Alanine aminotransferase (ALT)
pH	Creatinine
Protein	Gamma-glutamyl transferase (GGT)
Glucose	
Ketones	Ethanol testing[a]
Bilirubin	Urine drug screen[a]
Urobilinogen	Hepatitis B surface antigen
Blood	Hepatitis B core antibody (HBcAb)
Nitrite	Hepatitis C antibody
Microscopy[b]	HIV
	Pregnancy test
	FSH
	Serum immunoglobulins (IgG, IgM, IgA)

Abbreviations: FSH = follicle-stimulating hormone; HIV = human immunodeficiency virus; RBC = red blood cells; WBC = white blood cells.

a Urine drug screen and ethanol level may be repeated prior to admission to the clinical research unit.

b If dipstick results are abnormal.

Appendix 3. Study Governance, Regulatory and Ethical Considerations

Informed Consent

The investigator is responsible for:

- ensuring that the participant understands the nature of the study, the potential risks and benefits of participating in the study, and that their participation is voluntary.
- ensuring that informed consent is given by each participant or legal representative. This includes obtaining the appropriate signatures and dates on the informed consent form (ICF) prior to the performance of any protocol procedures and prior to the administration of investigational product.
- answering any questions the participant may have throughout the study and sharing in a timely manner any new information that may be relevant to the participant's willingness to continue his or her participation in the study.
- providing a copy of the ICF to the participant or the participant's legal representative and retaining a copy on file.

Recruitment

Lilly or its designee is responsible for the central recruitment strategy for participants. Individual investigators may have additional local requirements or processes. Study-specific recruitment material should be approved by Lilly.

Ethical Review

The investigator must give assurance that the ethical review board (ERB) was properly constituted and convened as required by International Conference on Harmonisation (ICH) guidelines and other applicable laws and regulations.

Documentation of ERB approval of the protocol and the ICF must be provided to Lilly before the study may begin at the investigative site(s). Lilly or its representatives must approve the ICF before it is used at the investigative site(s). All ICFs must be compliant with the ICH guideline on GCP.

The study site's ERB(s) should be provided with the following:

- the current IB and updates during the course of the study
- ICF
- relevant curricula vitae

Regulatory Considerations

This study will be conducted in accordance with the protocol and with:

1) consensus ethics principles derived from international ethics guidelines, including the Declaration of Helsinki and Council for International Organizations of Medical Sciences (CIOMS) International Ethical Guidelines
2) applicable ICH good clinical practice (GCP) Guidelines
3) applicable laws and regulations

Some of the obligations of the sponsor will be assigned to a third-party organization.

Protocol Signatures

The sponsor's responsible medical officer will approve the protocol, confirming that, to the best of his or her knowledge, the protocol accurately describes the planned design and conduct of the study.

After reading the protocol, each principal investigator will sign the protocol signature page and send a copy of the signed page to a Lilly representative.

Final Report Signature

The final report coordinating investigator or designee will sign the clinical study report for this study, indicating agreement that, to the best of his or her knowledge, the report accurately describes the conduct and results of the study.

The investigator with the most enrolled participants will serve as the final report coordinating investigator. If this investigator is unable to fulfill this function, another investigator will be chosen by Lilly to serve as the final report coordinating investigator.

The sponsor's responsible medical officer and statistician will sign/approve the final clinical study report for this study, confirming that, to the best of his or her knowledge, the report accurately describes the conduct and results of the study.

Data Quality Assurance

To ensure accurate, complete, and reliable data, Lilly or its representatives will do the following:

- provide instructional material to the study sites, as appropriate.
- provide training to instruct the investigators and study coordinators. This training will give instruction on the protocol, the completion of the electronic case report forms (eCRFs), and study procedures.
- make periodic visits to the study site.
- be available for consultation and stay in contact with the study site personnel by mail, telephone, and/or fax.
- review and evaluate eCRF data and/or use standard computer edits to detect errors in data collection.
- conduct a quality review of the database.

In addition, Lilly or its representatives will periodically check a sample of the participant data recorded against source documents at the study site. The study may be audited by Lilly and/or regulatory agencies at any time. Investigators will be given notice before an audit occurs.

The investigator will keep records of all original source data. This might include laboratory tests, medical records, and clinical notes. If requested, the investigator will provide the sponsor, applicable regulatory agencies, and applicable ERBs with direct access to the original source documents.

Data Collection Tools/Source Data

An electronic data capture system will be used in this study. The site must define and retain all source records and must maintain a record of any data where source data are directly entered into the data capture system.

Data Protection

Data systems used for the study will have controls and requirements in accordance with local data protection law.

The purpose and use of participant personal information collected will be provided in a written document to the participant by the sponsor.

Study and Site Closure

Discontinuation of Study Sites

Study site participation may be discontinued if Lilly or its designee, the investigator, or the ERB of the study site judges it necessary for medical, safety, regulatory, or other reasons consistent with applicable laws, regulations, and GCP.

Discontinuation of the Study

The study will be discontinued if Lilly or its designee judges it necessary for medical, safety, regulatory, or other reasons consistent with applicable laws, regulations, and GCP.

Appendix 4. Hepatic Monitoring Tests for Treatment-Emergent Abnormality

Selected tests may be obtained in the event of a treatment-emergent hepatic abnormality and may be required in follow-up with participants in consultation with the Lilly or designee clinical research physician.

Hepatic Monitoring Tests

**Hepatic Hematology**[a]	**Haptoglobin**[a]
Hemoglobin	
Hematocrit	**Hepatic Coagulation**[a]
RBC	Prothrombin Time
WBC	Prothrombin Time, INR
Neutrophils	
Lymphocytes	**Hepatic Serologies**[a,b]
Monocytes	Hepatitis A antibody, total
Eosinophils	Hepatitis A antibody, IgM
Basophils	Hepatitis B surface antigen
Platelets	Hepatitis B surface antibody
	Hepatitis B Core antibody
**Hepatic Chemistry**[a]	Hepatitis C antibody
Total bilirubin	Hepatitis E antibody, IgG
Conjugated bilirubin	Hepatitis E antibody, IgM
Alkaline phosphatase	
ALT	**Anti-nuclear antibody**[a]
AST	**Alkaline Phosphatase Isoenzymes**[a]
GGT	**Anti-smooth muscle antibody (or anti-actin**
CPK	**antibody)**[a]

Abbreviations: ALT = alanine aminotransferase; AST = aspartate aminotransferase; CPK = creatinine phosphokinase; GGT = gamma-glutamyl transferase; Ig = immunoglobulin; INR = international normalized ratio; RBC = red blood cells; WBC = white blood cells.

a Assayed by Lilly-designated or local laboratory.

b Reflex/confirmation dependent on regulatory requirements and/or testing availability

Appendix 5. Blood Sampling Summary

These tables summarize the approximate number of venipunctures and blood volumes for all blood sampling (screening, safety laboratories, and bioanalytical assays) during Part A, Part B, and Part C of the study.

Protocol J1B-MC-FRCC Part A Sampling Summary

Purpose	Blood Volume per Sample (mL)	Number of Blood Samples	Total Volume (mL)
Screening tests[a]	45	1	45
Clinical laboratory tests[a]	5	11	55
Safety Flow Panel (flow cytometry)	2	6	12
Immunoglobulins (IgG, IgM, IgA)	2.5	3	7.5
Pharmacokinetics (IV/SC Administration)	2	13/12	26/24
Blood discard for cannula patency (IV/SC Administration)	1	60/58	60/58
Pharmacodynamics (RO) (IV/SC Administration)	5	10/10	50/50
Immunogenicity	10	4	40
Pharmacogenetics	10	1	10
Exploratory Samples (IV/SC Administration)	10	11/10	110/100
Total (IV/SC Administration)	92.5	120/116	415.5/401.5
Total for clinical purposes [rounded up to nearest 10 mL] (IV/SC Administration)			420/410

Abbreviations: Ig = immunoglobulin; IV = intravenous; RO = receptor occupancy; SC = subcutaneous.

a Additional samples may be drawn if needed for safety purposes.

Protocol J1B-MC-FRCC Part B Sampling Summary

Purpose	Blood Volume per Sample (mL)	Number of Blood Samples	Total Volume (mL)
Screening tests[a]	45	1	45
Clinical laboratory tests[a]	5	7	35
Safety Flow Panel (flow cytometry)	2	5	10
Immunoglobulins (IgG, IgM, IgA)	2.5	4	10
Pharmacokinetics	2	22	44
Blood discard for cannula patency	1	84	84
Pharmacodynamics (RO)	5	19	95
Immunogenicity	10	4	40
Pharmacogenetics	10	1	10
Exploratory Samples	10	21	210
Total	92.5	168	583
Total for clinical purposes [rounded up to nearest 10 mL]			590

Abbreviations: Ig = immunoglobulin; RO = receptor occupancy.

a Additional samples may be drawn if needed for safety purposes.

Protocol J1B-MC-FRCC Part C Sampling Summary

Purpose	Blood Volume per Sample (mL)	Number of Blood Samples	Total Volume (mL)
Screening tests[a]	45	1	45
Clinical laboratory tests[a]	5	9	45
Safety Flow Panel (flow cytometry)	2	5	10
Immunoglobulins (IgG, IgM, IgA)	2.5	2	5
Pharmacokinetics	2	18	36
Blood discard for cannula patency	1	74	74
Pharmacodynamics (RO)	5	10	50
IL-19	2.5	11	27.5
Immunogenicity	10	5	50
Pharmacogenetics	10	1	10
Exploratory Samples[b] (serum, plasma, and mRNA)	16	4	64
Exploratory Samples[c] (serum only)	10	8	80
Total	111	148	496.5
Total for clinical purposes [rounded up to nearest 10 mL]			500

Abbreviations: Ig = immunoglobulin; IL = interleukin; IV = intravenous; mRNA= messenger ribonucleic acid; RNA = ribonucleic acid; RO = receptor occupancy.

a Additional samples may be drawn if needed for safety purposes.

b To be collected on all study days indicated in the Schedule of Activities (Section 2), except for Days 1 and 85.

c To be collected on Days 1 and 85, as indicated in the Schedule of Activities (Section 2).

Appendix 6. Validated Investigator Global Assessment Scale for Atopic Dermatitis (vIGA-AD)

Appendix 7. Protocol Amendment J1B-MC-FRCC(e) Summary Phase 1, Multicenter, Randomized, Placebo-Controlled, Triple-Blind, Single-Ascending Dose and Repeat-Dose Trial in Healthy Participants and Participants with Atopic Dermatitis

Overview

Protocol J1B-MC-FRCC, Phase 1, Multicenter, Randomized, Placebo-Controlled, Triple-Blind, Single-Ascending Dose and Repeat-Dose Trial in Healthy Participants and Participants with Atopic Dermatitis, has been amended.

The new protocol is indicated by Amendment (e) and will be used to conduct the study in place of any preceding version of the protocol.

This amendment is considered nonsubstantial based on the criteria set forth in Article 10(a) of Directive 2001/20/EC of the European Parliament and the Council of the European Union because it neither significantly impacts the safety or physical/mental integrity of participants nor the scientific value of the study.

The overall changes and rationale for the changes made to this protocol are as follows:

- Section 10.3.8 detailing about unblinded interim analysis for Part C to support subsequent clinical development planning.
- Added Section 10.3.9 detailing internal assessment committee.

Revised Protocol Sections

**Note:**	All deletions have been identified by ~~strikethroughs~~.<br>All additions have been identified by the use of underscore.

10.3.8 Interim Analysis

An interim analysis will occur after all planned subjects in Part A and Part B complete Day 29 (Visit 8). The analysis will include a review of safety and tolerability data available at this time; inclusive of laboratory data through, at minimum, Day 29. Any additional data available will also be reviewed.

An unblinded interim analysis for Part C is planned when approximately 30 patients have had the opportunity to complete, at a minimum, the Week 12 visit. If more than 30 patients are required to be enrolled to ensure 30 completers, the Internal Assessment Committee (IAC) may recommend an additional unblinded interim analysis for Part C.

Interim analyses may be conducted using efficacy and/or safety data. The planned and any additional interim analyses are to support subsequent clinical development planning.

Assessments of unblinded interim data, such as efficacy data, will be conducted by an IAC with a limited number of prespecified team members who do not have direct site contact or data entry or data validation responsibilities (see Section 10.3.9). Only the IAC will be authorized to evaluate unblinded interim efficacy and safety analyses. Study sites will receive information about interim results if a safety concern is identified.

Unblinding details will be specified in the unblinding plan section of the statistical analysis plan or in a separate unblinding plan document.

~~While not planned, there may be additional interim analyses.~~

~~The planned and any additional interim analyses are to support subsequent clinical development planning.~~

10.3.9 Data Monitoring Committee (DMC)

Not applicable. An IAC will be used to conduct interim analysis of unblinded data. The IAC review will be fully independent from the study team and will include, at a minimum, a Lilly medical physician, a statistician, and a representative from the Lilly Global Patient Safety organization. Details about the IAC membership, purpose, responsibilities, and operation will be described in an IAC charter, which will be approved prior to the first unblinding.

Leo Document ID = 6a7951f7-957b-42d0-8d0b-204ddae55613

Approver: PPD

Approval Date & Time: 16-Apr-2021 18:44:12 GMT

Signature meaning: Approved

Approver: PPD

Approval Date & Time: 19-Apr-2021 14:03:56 GMT

Signature meaning: Approved

STATISTICAL ANALYSIS PLAN

Phase 1, Multicenter, Randomized, Placebo-Controlled, Triple-Blind, Single-Ascending Dose and Repeat-Dose Trial in Healthy Participants and Participants with Atopic Dermatitis

Statistical Analysis Plan Status: Final (Working Version)
Statistical Analysis Plan Version: 2.0
Statistical Analysis Plan Date: 24-May-2021

Study Drug: LY3454738

Sponsor Reference: J1B-MC-FRCC
Covance CRU Study: 1000071-8398199

Clinical Phase I

1. TABLE OF CONTENTS

1. TABLE OF CONTENTS 2
2. ABBREVIATIONS 4
3. INTRODUCTION 7
4. STUDY OBJECTIVES 7
4.1 Primary Objectives 7
4.2 Secondary Objectives 7
4.3 Exploratory Objectives 8
5. STUDY DESIGN 8
5.1 Part A (SAD Design in Healthy Participants) 10
5.2 Part B (Repeat-Dose Design in Healthy Participants) 10
5.3 Part C (Repeat-Dose Design in Participants with AD) 10
6. TREATMENTS 11
7. SAMPLE SIZE JUSTIFICATION 11
8. DEFINITION OF ANALYSIS POPULATIONS 12
9. STATISTICAL METHODOLOGY 12
9.1 General 12
9.2 Demographics and Subject Disposition 13
9.3 Pharmacokinetic Assessment 13
9.3.1 Pharmacokinetic Analysis 13
9.3.2 Pharmacokinetic Statistical Methodology 17
9.4 Pharmacodynamic Assessment 18
9.4.1 Pharmacodynamic Parameter Estimation 18
9.4.2 Pharmacokinetic/Pharmacodynamic Analyses 18
9.5 Safety and Tolerability Assessments 18
9.5.1 Adverse events 18
9.5.2 Concomitant medication 19
9.5.3 Clinical laboratory parameters 19
9.5.4 Vital signs 19
9.5.5 Electrocardiogram (ECG) 19
9.5.6 Hepatic Monitoring 19
9.5.7 Immunogenicity 20
9.5.8 Flow Cytometry 20
9.5.9 Serum Immunoglobulins 20
9.5.10 Other assessments 20

9.6 Efficacy Assessments 20
10. DATA REVIEW DURING THE STUDY 22
11. INTERIM ANALYSES 22
12. CHANGES FROM THE PROTOCOL SPECIFIED STATISTICAL ANALYSES 22
13. REFERENCES 23
14. DATA PRESENTATION 23
14.1 Derived Parameters 23
14.2 Missing Data 23
14.3 Insufficient Data for Presentation 23
15. APPENDICES 24
Appendix 1: Document History 24

2. ABBREVIATIONS

Abbreviations pertain to the Statistical Analysis Plan (SAP) only (not the tables, figures and listings [TFLs]).

AD	Atopic dermatitis
ADA	Antidrug antibody
AE	Adverse event
ALP	Alkaline phosphatase
ALT	Alanine aminotransferase
ANCOVA	Analysis of covariance
AUC	Area under the concentration versus time curve
AUC(0-∞)	Area under the concentration versus time curve from time zero to infinity
AUC(0-τ)	Area under the concentration versus time curve during one dosing interval
AUC(0-t_{last})	Area under the concentration versus time curve from time zero to time t, where t is the last time point with a measurable concentration
%AUC(t_{last}-∞)	Percentage of AUC(0-∞) extrapolated
BQL	Below the quantifiable lower limit of the assay
C_{max}	Maximum observed drug concentration
CI	Confidence interval
CL	Total body clearance of drug calculated after intravenous administration
CL/F	Apparent total body clearance of drug calculated after extra-vascular administration
CRF	Case Report Form
CRU	Clinical Research Unit
CSR	Clinical Study Report
CTCAE	Common Terminology Criteria for Adverse Events
CV	Coefficient of variation
EC	Early Clinical
ECG	Electrocardiogram
e.g.	For example (Latin: *exempli gratia*)
IAC	Internal Assessment Committee

ICH	International Conference on Harmonisation
ITT	Intent-to-Treat
IV	Intravenous
LLOQ	Lower limit of quantification
LS	Least squares
MedDRA	Medical Dictionary for Regulatory Activities
MMRM	Mixed model repeated measures
MRE	Magnetic resonance elastography
PD	Pharmacodynamic
PK	Pharmacokinetic
PRO	Patient related outcomes
QoL	Quality of Life
R_A	Accumulation ratio based upon AUC(0-τ)
RO	Receptor occupancy
SAD	Single-ascending dose
SAP	Statistical Analysis Plan
SC	Subcutaneous
SD	Standard deviation
TBL	Total bilirubin
TE ADA+	Treatment-emergent antidrug antibody positive
TFLs	Tables, Figures, and Listings
$t_{1/2}$	Half-life associated with the terminal rate constant (λ_z) in non-compartmental analysis
t_{max}	Time of maximum observed drug concentration
ULN	Upper limit of normal
vIGA-AD	Validated Investigator's Global Assessment for Atopic Dermatitis
V_{SS}	Volume of distribution at steady state following intravenous administration
$V_{SS/F}$	Apparent volume of distribution at steady state after extra-vascular administration
V_z	Apparent volume of distribution during the terminal phase after intravenous administration
V_z/F	Apparent volume of distribution during the terminal phase after extra-vascular administration

WHO	World Health Organization

3. INTRODUCTION

This SAP has been developed after review of the Clinical Study Protocol Amendment (e) (final version dated 19 April 2021).

This SAP describes the planned analysis of the safety, tolerability, pharmacokinetic (PK) and pharmacodynamic (PD) data from this study. A detailed description of the planned TFLs to be presented in the clinical study report (CSR) is provided in the accompanying TFL shell document.

The intent of this document is to provide guidance for the statistical and PK analyses of data. In general, the analyses are based on information from the protocol, unless they have been modified by agreement between Eli Lilly and Company and Covance Early Clinical (EC) Biometrics. A limited amount of information concerning this study (e.g. objectives, study design) is given to help the reader's interpretation. This SAP must be signed off prior to first subject administration for this study. When the SAP and TFL shells are agreed upon and finalized, they will serve as the template for this study's CSR.

This SAP supersedes the statistical considerations identified in the protocol; where considerations are substantially different, they will be so identified. If additional analyses are required to supplement the planned analyses described in this SAP, they may be performed and will be identified in the CSR. Any substantial deviations from this SAP will be agreed upon between Eli Lilly and Company and Covance EC Biometrics and identified in the CSR. Any minor deviations from the TFLs may not be documented in the CSR.

This SAP is written with consideration of the recommendations outlined in the International Conference on Harmonisation (ICH) E9 Guideline entitled Guidance for Industry: Statistical Principles for Clinical Trials[1] and the ICH E3 Guideline entitled Guidance for Industry: Structure and Content of Clinical Study Reports[2].

4. STUDY OBJECTIVES

4.1 Primary Objectives

- To assess the safety and tolerability of LY3454738 after single intravenous (IV) and subcutaneous (SC) dosing in healthy participants and after multiple IV dosing in healthy participants and participants with atopic dermatitis (AD).
- To evaluate the efficacy of LY3454738 after multiple IV dosing in participants with AD at Week 12.

4.2 Secondary Objectives

- To characterize the PK of LY3454738 following single (IV and SC) and multiple (IV) dosing administration in healthy participants and after multiple IV dosing in participants with AD.

- To evaluate the efficacy of LY3454738 over time after multiple IV dosing in participants with AD.

4.3 Exploratory Objectives

- To assess the potential development of anti-LY3454738 antibodies and their impact on safety and PK of LY3454738.
- To evaluate relationship of receptor occupancy (RO) versus dose after single and multiple dosing of LY3454738.
- To explore the PD response of LY3454738 after multiple IV dosing in participants with AD.
- To evaluate the efficacy of LY3454738 over time after multiple IV dosing in participants with AD as assessed by patient related outcomes (PRO)/Quality of Life (QoL) measures.
- To explore the potential associations between exposure and PD/clinical responses.
- To explore the safety, tolerability, PK, and RO of LY3454738 in Japanese participants in relation to non-Japanese participants.

5. STUDY DESIGN

Study J1B-MC-FRCC (FRCC) is a Phase 1, multicenter, randomized, placebo-controlled, triple-blind (i.e., blinded to investigator, participant, and sponsor staff who are involved in the treatment or clinical evaluation of the participants), single-ascending dose (SAD) and repeat-dose trial in healthy participants and in participants with AD to explore the safety, tolerability, PK, target engagement, and, in participants with AD, clinical PD and efficacy of LY3454738. A portion of the healthy participants will be Japanese.

There are 3 parts to this trial:

- Part A: SAD design in healthy participants (including healthy Japanese participants)
- Part B: Repeat-dose design in healthy participants (including healthy Japanese participants)
- Part C: Repeat-dose design in participants with AD.

Table FRCC.1 provides a detailed description of participant cohorts and planned doses for Parts A, B, and C. Figure FRCC.1 illustrates the study design and demonstrates the relationship among Parts A, B, and C. Part A will begin first and will inform and thereby trigger the initiation of Parts B and C.

In Parts A and B of the trial, participants will be dosed with LY3454738 or placebo at the clinical site on Day 1 (Part A) or Days 1 and 15 (Part B).

In Part C of the trial, participants will receive LY3454738 or placebo every 2 weeks for 12 weeks on an outpatient basis.

Table FRCC.1. Summary of Participant Cohorts

Cohort#	Planned Dose/ Administration Route	Number of Planned non-Japanese Participants		Number of Planned Japanese Participants		Total Number of Planned Participants
		LY	PBO	LY	PBO	
**Part A**						
SAD Cohort 1[a]	1 mg/IV	6	2	0	0	8
SAD Cohort 2[a]	5 mg/IV	6	2	0	0	8
SAD Cohort 3	25 mg/IV	6	2	0	0	8
SAD Cohort 4a	100 mg/IV	3	1	3	1	8
SAD Cohort 4b	100 mg/SC	3	0	3	0	6
SAD Cohort 5	300 mg/IV	3	1	3	1	8
SAD Cohort 6	1000 mg/IV	3	1	3	1	8
**Part B**						
Repeat-Dose Cohort	200 mg/IV	3	1	3	1	8
**Part C**						
Atopic Dermatitis	500 mg/IV	20	10	0	0	30

Abbreviations: IV = intravenous; LY = LY3454738; PBO = placebo; SAD = single-ascending dose; SC=subcutaneous.

[a] Sentinel dosing will be used in this cohort. Single-ascending dose cohorts 1 and 2 will include sentinel dosing for 2 participants (1 placebo and 1 LY3454738). If safety and tolerability are acceptable in these 2 participants, the remainder of the cohort can commence dosing 48 hours later.

Figure FRCC.1. Study design

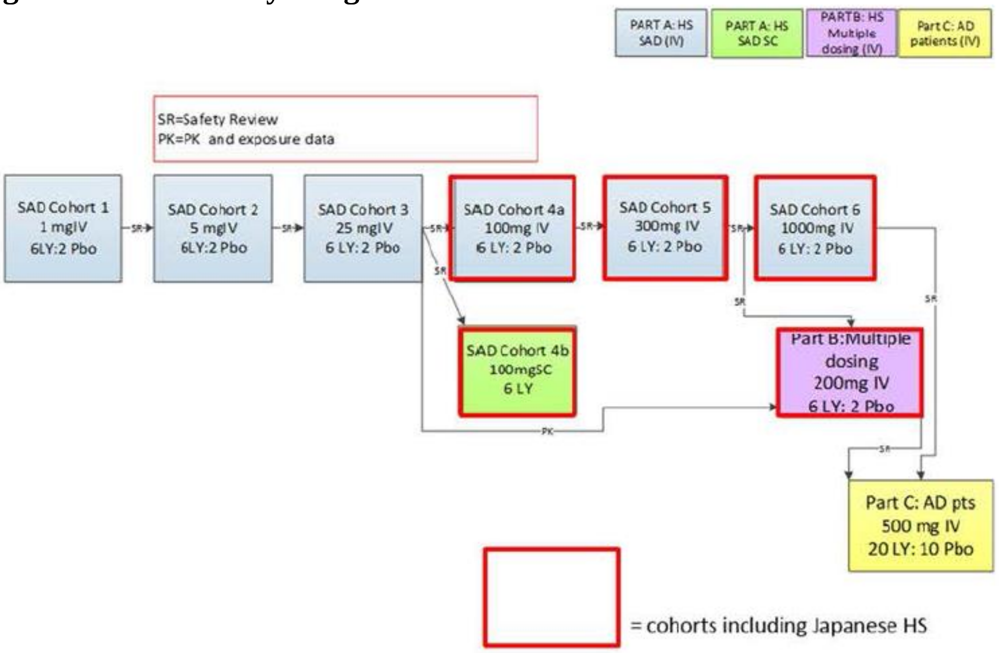

Abbreviations: AD = atopic dermatitis; HS = healthy subjects; IV = intravenous; LY = LY3454738; Pbo = placebo; PK = pharmacokinetics; pts = patients; SAD = single-ascending dose; SC = subcutaneous; SR = safety review.

5.1 Part A (SAD Design in Healthy Participants)

Single-ascending doses will be evaluated in Part A in healthy participants. Sentinel dosing will be used for 2 participants (1 placebo and 1 LY3454738) at each of the first 2 dose levels. With sentinel dosing, only 1 participant will be dosed at a time, and the second participant will not be dosed until the first participant's infusion is complete. If safety and tolerability are acceptable in these 2 participants, the remainder of the cohort can commence dosing 48 hours later. Non-sentinel participants will be dosed in sequence, each following, at a minimum, completion of the infusion for the prior participant.

Six cohorts with healthy participants are planned to be randomized to receive single IV doses of LY3454738 (1, 5, 25, 100, 300, 1000 mg) or placebo. Single-ascending dose Cohorts 4a, 5, and 6 are planned to include Japanese participants (4 per cohort [3 LY3454738 : 1 placebo]). A single open-label cohort (Cohort 4b) is planned to be enrolled to receive a single SC dose of 100 mg. Cohort 4b will include 6 healthy participants: approximately 3 non-Japanese participants and 3 Japanese participants. All participants in this cohort will receive LY3454738. This cohort can run in parallel with Cohort 4a, which will receive a single IV dose of 100 mg or placebo. Participants will be followed for 12 weeks after dosing.

A data review is scheduled to occur after each cohort, prior to dose escalation. Decisions regarding dose escalation in Part A will be made by the safety review committee after safety, tolerability, and exposure are reviewed.

5.2 Part B (Repeat-Dose Design in Healthy Participants)

A single cohort with healthy participants will be randomized to receive 2 biweekly IV doses of LY3454738 (200 mg) or placebo (Days 1 and 15). Part B will include 4 Japanese participants [3 LY3454738 : 1 placebo] and 4 non-Japanese participants [3 LY3454738 : 1 placebo]. The planned dose for this cohort is 200 mg; a dose level expected to have submaximal exposure in order to assess any acute safety/tolerability events associated with a second exposure. The actual dose may be adjusted up or down based on the data review analysis. The expected exposure at the end of dosing (area under the concentration versus time curve (AUC) from time 0 to 336 hours [AUC(0-336)]) in Part B (based on predicted accumulation) must have already been evaluated as a single dose. Participants will be dosed in sequence, each following, at a minimum, completion of the infusion for the prior participant. Participants will be followed for 12 weeks after the final treatment administration.

5.3 Part C (Repeat-Dose Design in Participants with AD)

A single cohort of participants with AD will be randomized 2:1 to receive IV dosing of LY3454738 or placebo, respectively, every 2 weeks for 12 weeks (dosing on Days 1, 15, 29, 43, 57, 71, and 85). The planned dose for this cohort is 500 mg. This dose is predicted to achieve a steady state exposure that is similar to the maximum exposure achieved after the maximum dose tested as a single dose (1000 mg). The actual dose in Part C may be modified after review of the prior exposure data and the PK and RO data from Cohorts 1, 2, and 3 in Part A. If more than 1 participant is at an individual site, these participants should be dosed in sequence. Participants will be followed for 12 weeks after the last dose.

6. TREATMENTS

The following is a list of the study treatment abbreviations that will be used in the TFLs.

Part A

Cohort	Study Treatment Name	Treatment order in TFL
	Placebo IV	1
SAD Cohort 1	1 mg LY3454738 IV	2
SAD Cohort 2	5 mg LY3454738 IV	3
SAD Cohort 3	25 mg LY3454738 IV	4
SAD Cohort 4a	100 mg LY3454738 IV	5
SAD Cohort 4b	100 mg LY3454738 SC	6
SAD Cohort 5	300 mg LY3454738 IV	7
SAD Cohort 6	1000 mg LY3454738 IV	8

Part B

Cohort	Study Treatment Name	Treatment order in TFL
Repeat-Dose Cohort	Placebo IV	1
Repeat-Dose Cohort	200 mg LY3454738 IV	2

Part C

Cohort	Study Treatment Name	Treatment order in TFL
Atopic Dermatitis	Placebo IV	1
Atopic Dermatitis	500 mg LY3454738 IV	2

7. SAMPLE SIZE JUSTIFICATION

The sample sizes in Part A (54 participants) and Part B (8 participants) are not based on statistical calculations, as these cohorts are designed primarily to seek information on safety, tolerability, PK, and target engagement. The sample sizes are not adjusted to account for dropouts. However, the protocol allows replacement if it is deemed necessary to obtain sufficient data for interpretation.

For Part C, assuming 50% of participants receiving LY3454738 and 8% of participants receiving placebo achieve a Validated Investigator’s Global Assessment for Atopic Dermatitis (vIGA-AD) 0/1, a sample size of 30 completers will provide approximately 70% power using a 2-sided Fisher’s exact test at the 0.10 significance level.

Participants who are randomized but not administered treatment may be replaced to ensure that approximately enough participants may complete the study.

8. DEFINITION OF ANALYSIS POPULATIONS

The “Safety” population will consist of all enrolled subjects, whether or not they completed all protocol requirements.

The “Pharmacokinetic” population will consist of all subjects who received at least one dose of study drug and have evaluable PK data.

The “Pharmacodynamic” population will consist of all subjects who received at least one dose of study drug or placebo and have evaluable PD data.

The “Intent-to-Treat (ITT)” population will consist of all randomized subjects, even if the subject does not take the assigned treatment, does not receive the correct treatment, or otherwise does not follow the protocol.

All protocol deviations that occur during the study will be considered for their severity/impact and will be taken into consideration when subjects are assigned to analysis populations.

9. STATISTICAL METHODOLOGY

9.1 General

In Part A and Part B, summary statistics, data tabulations and figures will be provided by population (overall, Japanese, and non-Japanese) to explore the safety, tolerability, PK and RO of LY3454738 in Japanese participants in relation to non-Japanese participants.

Data listings will be provided for all data that is databased. Summary statistics and statistical analysis will only be presented for data where detailed in this SAP. For continuous data, summary statistics will include the arithmetic mean, arithmetic standard deviation (SD), median, min, max and N; for log-normal data (e.g. the PK parameters: AUCs and maximum observed drug concentration [C_{max}]) the geometric mean and geometric coefficient of variation (CV%) will also be presented. For categorical data, frequency count and percentages will be presented. Data listings will be provided for all subjects up to the point of withdrawal, with any subjects excluded from the relevant population highlighted. Summary statistics and statistical analyses will generally only be performed for subjects included in the relevant analysis population. For the calculation of summary statistics and statistical analysis, unrounded data will be used.

Mean change from baseline is the mean of all individual subjects’ change from baseline values. Each individual change from baseline will be calculated by subtracting the individual subject’s baseline value from the value at the time point. The individual subject’s change from baseline values will be used to calculate the mean change from baseline using a SAS procedure such as Proc Univariate.

Data analysis will be performed using SAS® Version 9.4 or greater.

9.2 Demographics and Subject Disposition

Subject disposition will be listed. The demographic variables age, sex, race, ethnicity, body weight, height and body mass index will be summarized and listed. All other demographic variables will be listed only.

In Part C, baseline disease characteristics (vIGA-AD, Eczema Area and Severity Index [EASI], and SCORing Atopic Dermatitis [SCORAD]) will also be summarized and listed.

9.3 Pharmacokinetic Assessment

9.3.1 Pharmacokinetic Analysis

PK parameter estimates will be determined using non-compartmental procedures in validated software program (Phoenix WinNonlin Version 6.4 or later).

Serum concentrations of LY3454738 will be used to determine the following PK parameters, when possible:

Part A

Parameter	Units	Definition
$AUC(0\text{-}t_{last})$	h*µg/mL	area under the concentration versus time curve from time zero to time t, where t is the last time point with a measurable concentration
$AUC(0\text{-}\infty)$	h*µg/mL	area under the concentration versus time curve from time zero to infinity
$\%AUC(t_{last}\text{-}\infty)$	%	percentage of $AUC(0\text{-}\infty)$ extrapolated
C_{max}	µg/mL	maximum observed drug concentration
t_{max}	h	time of maximum observed drug concentration
$t_{½}$	h	half-life associated with the terminal rate constant (λz) in non-compartmental analysis
CL	L/h	total body clearance of drug calculated after intravenous administration
CL/F	L/h	apparent total body clearance of drug calculated after extra-vascular administration
V_z	L	apparent volume of distribution during the terminal phase after intravenous administration
V_z/F	L	apparent volume of distribution during the terminal phase after extra-vascular administration
V_{SS}	L	volume of distribution at steady state following intravenous administration
V_{SS}/F	L	apparent volume of distribution at steady state after extra-vascular administration

Part B and C

Parameter	Units	Definition
AUC(0-τ)	h*µg/mL	area under the concentration versus time curve during one dosing interval
C_{max}	µg/mL	maximum observed drug concentration
t_{max}	h	time of maximum observed drug concentration
$t_{½}$	h	half-life associated with the terminal rate constant (λz) in non-compartmental analysis
CL	L/h	total body clearance of drug calculated after intravenous administration
V_Z	L	volume of distribution during the terminal phase after intravenous administration
R_A	N/A	accumulation ratio based upon AUC(0-τ)

Trough (predose) serum concentrations of LY3463251 will be listed and summarized.

Additional PK parameters may be calculated, as appropriate.

The software and version used for the final analyses will be specified in the CSR. Any exceptions or special handling of data will be clearly documented within the final study report.

Formatting of tables, figures and abbreviations will follow the Eli Lilly Global PK/PD/TS Tool: NON-COMPARTMENTAL PHARMACOKINETIC STYLE GUIDE. The version of the tool effective at the time of PK analysis will be followed.

General PK Parameter Rules

- Actual sampling times will be used in the final analyses of individual PK parameters, except for non-bolus pre-dose sampling times which will be set to zero. For multiple dose profiles, the pre-dose time will be set to zero.

- C_{max} and t_{max} will be reported from observed values. If C_{max} occurs at more than one time point, t_{max} will be assigned to the first occurrence of C_{max}.

- AUC parameters will be calculated using a combination of the linear and logarithmic trapezoidal methods (linear-log trapezoidal rule). The linear trapezoidal method will be applied up to t_{max} and then the logarithmic trapezoidal method will be used after t_{max}. The minimum requirement for the calculation of AUC will be the inclusion of at least three consecutive concentrations above the lower limit of quantification (LLOQ), with at least one of these concentrations following C_{max}.

- AUC(0-∞) values where the percentage of the total area extrapolated is more than 20% will be flagged. Any AUC(0-∞) value excluded from summary statistics will be noted in the footnote of the summary table.

- Half-life ($t_{½}$) will be calculated, when appropriate, based on the apparent terminal log-linear portion of the concentration-time curve. The start of the terminal elimination phase for each subject will be defined by visual inspection and generally will be the first point at which there is no systematic deviation from the log-linear decline in serum concentrations. Half-life will only be calculated when a reliable estimate for this parameter can be obtained comprising of at least 3 data points. If $t_{½}$ is estimated over a time window of less than 2 half-lives, the values will be flagged in the data listings. Any $t_{½}$ value excluded from summary statistics will be documented in the footnote of the summary table.

- A uniform weighting scheme will be used in the regression analysis of the terminal log-linear portion of the concentration-time curve.

- The parameters based on predicted C_{last} will be reported.

Individual PK Parameter Rules

- Only quantifiable concentrations will be used to calculate PK parameters with the exception of special handling of certain concentrations reported below the lower limit of quantitation (BQL). Serum concentrations reported as BQL will be set to a value of zero when all of the following conditions are met:
    - The compound is non-endogenous.
    - The samples are from the initial dose period for a subject or from a subsequent dose period following a suitable wash-out period.
    - The time points occur before the first quantifiable concentration.

- All other BQL concentrations that do not meet the above criteria will be set to missing.

- Also, where two or more consecutive concentrations are BQL towards the end of a profile, the profile will be deemed to have terminated and therefore any further quantifiable concentrations will be set to missing for the calculation of the PK parameters unless it is considered to be a true characteristic of the profile of the drug.

- For multiple-dosing data, when pre-dose concentrations are missing, the value to be substituted will be C_{min} for the dosing interval.

Individual Concentration vs. Time Profiles

- Individual concentrations will be plotted utilizing actual sampling times.

- The terminal point selections will be indicated on a semi-logarithmic plot.

Average Concentration vs. Time Profiles

- The average concentration profiles will be graphed using scheduled (nominal) sampling times.
- The average concentration profiles will be graphed using arithmetic average concentrations.
- The pre-dose average concentration for single-dose data from non-endogenous compounds will be set to zero. Otherwise, only quantifiable concentrations will be used to calculate average concentrations.
- Concentrations at a sampling time exceeding the sampling time window specified in the protocol, or ± 10%, will be excluded from the average concentration profiles.
- Concentrations excluded from the mean calculation will be documented in the final study report.
- A concentration average will be plotted for a given sampling time only if 2/3 of the individual data at the time point have quantifiable measurements that are within the sampling time window specified in the protocol or ± 10%. An average concentration estimated with less than 2/3 but more than 3 data points may be displayed on the mean concentration plot if determined to be appropriate and will be documented within the final study report.

Treatment of Outliers during Pharmacokinetic Analysis

Application of this procedure to all PK analyses is not a requirement. Rather, this procedure provides justification for exclusion of data when scientifically appropriate. This procedure describes the methodology for identifying an individual value as an outlier for potential exclusion, but does not require that the value be excluded from analysis. The following methodology will not be used to exclude complete profiles from analysis.

Data within an Individual Profile
A value within an individual profile may be excluded from analysis if any of the following criteria are met:

- For PK profiles during single dosing of non-endogenous compounds, the concentration in a pre-dose sample is quantifiable.
- For any questionable datum that does not satisfy the above criteria, the profile will be evaluated and results reported with and without the suspected datum.

Data between Individual Profiles

1. If n<6, then the dataset is too small to conduct a reliable range test. Data will be analyzed with and without the atypical value, and both sets of results will be reported.

2. If n≥6, then an objective outlier test will be used to compare the atypical value to other values included in that calculation:

    a. Transform all values in the calculation to the logarithmic domain.

    b. Find the most extreme value from the arithmetic mean of the log transformed values and exclude that value from the dataset.

    c. Calculate the lower and upper bounds of the range defined by the arithmetic mean ±3*SD of the remaining log-transformed values.

    d. If the extreme value is within the range of arithmetic mean ±3*SD, then it is not an outlier and will be retained in the dataset.

    e. If the extreme value is outside the range of arithmetic mean ±3*SD, then it is an outlier and will be excluded from analysis.

If the remaining dataset contains another atypical datum suspected to be an outlier and n ≥ 6 following the exclusion, then repeat step 2 above. This evaluation may be repeated as many times as necessary, excluding only one suspected outlier in each iteration, until all data remaining in the dataset fall within the range of arithmetic mean ±3*SD of the log-transformed values.

Reporting of Excluded Values
Individual values excluded as outliers will be documented in the final report. Approval of the final report will connote approval of the exclusion.

9.3.2 Pharmacokinetic Statistical Methodology

PK parameters will be evaluated to estimate dose proportionality based on data in the SAD part of the study. Log-transformed C_{max} and AUC parameters will be evaluated using a power model (where log dose acts as an explanatory variable) to estimate ratios of dose normalized geometric means and corresponding 90% confidence intervals (CIs). The estimated ratio of dose-normalized geometric means of PK parameters between the highest and lowest doses will be used to assess dose proportionality. A subinterval within the highest and lowest doses may also be considered for assessment of dose proportionality using the same approach. For the analysis of the dose proportionality assessment, the data from the placebo group will be excluded. Additional analyses following SC administration may be performed, if possible.

Example SAS code for the analysis:

```
proc mixed data=pk1;
   model lvar=ldose / alpha=0.1 cl solution residual ddfm=kr;
   estimate 'xx mg' intercept 1 ldose a / cl; /*a=Log value of xx*/
   estimate 'yy mg' intercept 1 ldose b / cl; /*b=Log value of yy*/
   estimate 'zz mg' intercept 1 ldose c / cl; /*c=Log value of zz*/
   estimate 'zz mg - xx mg' ldose pp / alpha=0.1 cl; /*pp=Difference in log values of zz and xx*/
   ods output solutionf=est;
   ods output estimates=estims;
 run;
```

9.4 Pharmacodynamic Assessment

9.4.1 Pharmacodynamic Parameter Estimation

PD analysis will be reported as %RO per participant over time based on LY3454738 binding to neutrophils from whole blood. %RO will be summarized over time by treatment, and listed. Figures of the mean %RO profile and individual %RO profiles over time will be presented by treatment. Lilly will be responsible for this analysis.

9.4.2 Pharmacokinetic/Pharmacodynamic Analyses

The relationship between LY3454738 exposure, RO and other PD endpoints may be explored with graphical analysis for Parts A, B and C. If the relationship is deemed to exist, a population PK/PD analysis approach may be applied. Lilly will be responsible for this analysis.

The PD parameters for analysis will be maximum receptor occupancy (RO_{max}) and dose or concentration to achieve 50% RO_{max} (ED50 or EC50, respectively). Estimation of other PD parameters may be performed, if deemed appropriate. Figures of the mean serum IL-19 profiles and individual IL-19 profiles over time will be presented. Lilly will be responsible for this analysis.

9.5 Safety and Tolerability Assessments

9.5.1 Adverse events

Where changes in severity are recorded in the Case Report Form (CRF), each separate severity of the adverse event (AE) will be reported in the listings, only the most severe will be used in the summary tables. A pre-existing condition is defined as an AE that starts before the subject has provided written informed consent and is ongoing at consent. A non-treatment emergent AE is defined as an AE which starts after informed consent but prior to dosing. A treatment-emergent AE is defined as an AE which occurs postdose or which is present prior to dosing and becomes more severe postdose.

All AEs will be listed. Treatment-emergent AEs will be summarized by treatment, severity and relationship to the study drug. The frequency (the number of AEs, the number of subjects experiencing an AE and the percentage of subjects experiencing an AE) of treatment-emergent AEs will be summarized by treatment, Medical Dictionary for Regulatory Activities (MedDRA) version 21.0 system organ class and preferred term. Infusion and injection site reactions will be summarized by maximum Common Terminology Criteria for Adverse Events (CTCAE) grade.

The summary and frequency AE tables will be presented for all causalities and those considered related to the study drug. Any serious AEs will be listed. AEs by day of onset will be presented for the Part B and Part C data.

9.5.2 Concomitant medication

Concomitant medication will be coded using the world health organization (WHO) drug dictionary (Version March 2018). Concomitant medication will be listed.

9.5.3 Clinical laboratory parameters

All clinical chemistry and hematology data will be summarized by parameter and treatment, and listed. Urinalysis data will be listed. Additionally clinical chemistry, hematology and urinalysis data outside the reference ranges will be listed.

Values for any clinical chemistry, hematology and urinalysis values outside the reference ranges will be flagged on the individual subject data listings.

9.5.4 Vital signs

Vital signs data will be summarized by treatment together with changes from baseline, where baseline is defined as the Day 1 predose assessment. Figures of mean vital signs and mean changes from baseline profiles will be presented by treatment. Furthermore, values for individual subjects will be listed.

9.5.5 Electrocardiogram (ECG)

ECGs will be performed for safety monitoring purposes only and will not be presented. Any clinically significant findings from ECGs will be reported as an AE.

9.5.6 Hepatic Monitoring

If a subject experiences elevated alanine aminotransferase (ALT) ≥3× upper limit of normal (ULN), alkaline phosphatase (ALP) ≥2× ULN, or elevated total bilirubin (TBL) ≥2× ULN, liver tests will be performed to confirm the abnormality. Additional safety data may be collected if required, as defined in the protocol. Where applicable, the following will be presented.

The subjects' liver disease history and associated person liver disease history data will be listed. Any concomitant medication of acetaminophen/paracetamol will be listed. Results from any hepatic monitoring procedures, such as a magnetic resonance elastography (MRE) scan, and a biopsy assessment will be listed, if performed.

Hepatic risk factor assessment data will be listed. Liver related signs and symptoms data will be summarized by treatment and listed. Alcohol and recreational drug use data will also be listed.

All hepatic chemistry, hematology, coagulation, and serology data will be listed. Values outside the reference ranges will be flagged on the individual subject data listings.

9.5.7 Immunogenicity

The frequency and percentage of participants with preexisting antidrug antibody (ADA) and with treatment-emergent antidrug antibody positive (TE ADA+) to LY3454738 will be tabulated. Treatment-emergent ADAs are defined as those with a titer 2-fold (1 dilution) greater than the minimum required dilution if no ADAs were detected at baseline (treatment-induced ADA) or those with a 4-fold (2 dilutions) increase in titer compared to baseline if ADAs were detected at baseline (treatment-boosted ADA). For the TE ADA+ participants, the distribution of maximum titers will be described. The frequency of neutralizing antibodies will also be tabulated in TE ADA+ participants.

The relationship between the presence of antibodies and the PK parameters and PD response including safety and efficacy to LY3454738 may be assessed.

9.5.8 Flow Cytometry

Target engagement will be assessed using a whole blood assay measuring the binding of LY3454738 to CD200R expressed on neutrophils by flow cytometry. These data will be listed. Flow cytometry data will be summarized by treatment

9.5.9 Serum Immunoglobulins

Serum immunoglobulins (M, G, A and total) will be summarized by treatment and listed.

9.5.10 Other assessments

All other safety assessments not detailed in this section will be listed but not summarized or statistically analyzed.

9.6 Efficacy Assessments

Efficacy analyses will be performed for Part C of the study only.

Fisher’s exact test will be used for treatment comparisons of discrete efficacy variables. The percentages and difference in percentages will be reported. Continuous efficacy variables will be analyzed by a mixed model repeated measures (MMRM) to estimate the least squares (LS) means and corresponding 90% CIs. Descriptive statistics will be reported. Mean vIGA-AD score, and mean percent change from baseline in EASI and SCORAD, will be plotted up to Week 24. Individual scores will also be plotted up to Week 24.

Primary Analyses

The primary efficacy measure is the binary outcome of response defined as a vIGA-AD score of 0 or 1 (clear or almost clear skin) with a ≥ 2 point improvement from baseline at Week 12. The primary analysis will be conducted using Fisher’s exact test.

Secondary Analyses

Fisher's exact test will be used to analyze the proportion of participants achieving

- vIGA-AD of 0 or 1 with a ≥ 2 point improvement from baseline at Week 1 through Week 12.

- EASI50, EASI75, and EASI90 at Week 1 through Week 12, where EASI50, EASI75, and EASI90 are defined as having an improvement of at least 50%, 75%, and 90% from baseline, respectively.

- SCORAD50, SCORAD75 and SCORAD90 at Week 1 through Week 12, where SCORAD50, SCORAD75 and SCORAD90 are defined as having an improvement of at least 50%, 75% and 90% from baseline, respectively.

Change from baseline and percent change from baseline in EASI and SCORAD will be analysed using a MMRM. The model will include fixed factors for treatment, time and treatment-by-time interaction, baseline as a covariate, and subject as random effect. An unstructured covariance matrix will be used to account for within-subject variability. The LS mean, treatment difference, corresponding 90% CI and p-value will be presented. The analysis will be performed separately including all data up to Week 12, and all data up to Week 24 (percent change only), presenting the overall LS means for each treatment and difference in LS means (LY3454738-Placebo). Additionally, LS means and differences will be presented by timepoint up to Week 24 for percent change from baseline.

Example SAS code for the Fisher's exact test analysis:

```
proc freq data=saf1;
   tables resp*treatment / exact;
   weight count;
   ods output fishersexact=fishers1;
run;
```

Example SAS code for the MMRM:

```
proc mixed data=saf1 method=ml;
   by param1 param2;
   class treatment timepoint subj;
   model change = base treatment timepoint treatment*timepoint / ddfm=kr cl residual;
   repeated timepoint / subject=subj type=un;
   lsmeans treatment / alpha=0.1 cl pdiff;
   lsmeans treatment*timepoint / alpha=0.1 cl pdiff;
   ods output lsmeans=lsmeans1;
   ods output diffs=diff;
run;
```

Exploratory Analyses

Improvement in signs and symptoms, health outcome measures, and QoL measures (total scores, item scores, and derivations) will be summarized using descriptive statistics and the previously described models for discrete and continuous endpoints.

10. DATA REVIEW DURING THE STUDY

Access to the data is scheduled to occur after each cohort prior to dose escalation, and before initiation of the multiple-dose cohorts, in order to review emerging safety, tolerability, drug exposure, and target engagement data. The purpose of these reviews is to guide dose selection for the next dosing session, and distribution of Japanese and non-Japanese participants, in addition to informing the design of subsequent studies.

A data review of safety/tolerability, PK, and target engagement data will occur when at least 6 participants in Cohort 3 have available data at Day 15. The assessment will mainly include data from Cohorts 1, 2, and 3 of Part A. The data from this data review, along with the additional data detailed in Section 7.4 of the protocol, will be used to confirm or modify the specified dose levels for Cohorts 5 and 6 of Part A and the doses for Parts B and C of this study.

The investigator and the Lilly sponsor team will make the determination regarding dose escalation based upon their review of the data. The investigator will remain blinded and the Lilly sponsor team will be unblinded during these reviews.

11. INTERIM ANALYSES

An interim analysis will occur after all planned subjects in Part A and Part B complete Day 29 (Visit 8). The analysis will include a review of safety and tolerability data available at this time; inclusive of laboratory data through, at minimum, Day 29. Any additional data available will also be reviewed.

An unblinded interim analysis for Part C is planned when approximately 30 patients have had the opportunity to complete, at a minimum, the Week 12 visit. If more than 30 patients are required to be enrolled to ensure 30 completers, the Internal Assessment Committee (IAC) may recommend an additional unblinded interim analysis for Part C.

Interim analyses may be conducted using efficacy and/or safety data. The planned and any additional interim analyses are to support subsequent clinical development planning.

Assessments of unblinded interim data, such as efficacy data, will be conducted by an IAC with a limited number of prespecified team members who do not have direct site contact or data entry or data validation responsibilities. Only the IAC will be authorized to evaluate unblinded interim efficacy and safety analyses. Study sites will receive information about interim results if a safety concern is identified.

Unblinding details will be specified in a separate unblinding plan document.

12. CHANGES FROM THE PROTOCOL SPECIFIED STATISTICAL ANALYSES

Change from baseline in EASI and SCORAD will be analysed using a MMRM as detailed in Section 9.6, not an analysis of covariance (ANCOVA). Percent change from baseline will also be analysed.

13. REFERENCES

1. International Conference on Harmonization of Technical Requirements for Registration of Pharmaceuticals for Human Use, ICH Harmonized Tripartite Guideline, Statistical Principles for Clinical Trials (E9), 5 February 1998.

2. International Conference on Harmonization of Technical Requirements for Registration of Pharmaceuticals for Human Use, ICH Harmonized Tripartite Guideline, Structure and Content of Clinical Study Reports (E3), 30 November 1995.

14. DATA PRESENTATION

14.1 Derived Parameters

Individual derived parameters (e.g. PK parameters) and appropriate summary statistics will be reported to three significant figures. Observed concentration data, e.g. C_{max}, should be reported as received. Observed time data, e.g. t_{max}, should be reported as received. N and percentage values should be reported as whole numbers. Median values should be treated as an observed parameter and reported to the same number of decimal places as minimum and maximum values.

14.2 Missing Data

Missing data will not be displayed in listings.

14.3 Insufficient Data for Presentation

Some of the TFLs may not have sufficient numbers of subjects or data for presentation. If this occurs, the blank TFL shell will be presented with a message printed in the centre of the table, such as, “No serious adverse events occurred for this study.”

15. APPENDICES

Appendix 1: Document History

Status and Version	Date of Change	Summary/Reason for Changes
Final Version 1.0	NA	NA; the first version.
Final Version 2.0	24May2021	Interim Analyses details added in line with Protocol Amendment (e).<br>Other minor edits for consistency with Protocol and TFL Shells.

NA = not applicable
