# Supplementary material for: Ucenprubart is an agonistic antibody to CD200R with the potential to treat inflammatory skin disease: preclinical development and a phase 1 clinical study
Source: Nat Commun. 2025 May 1;16:4082. doi: 10.1038/s41467-025-59147-w (PMC12046042; doi:10.1038/s41467-025-59147-w)
Supplement: Supplementary file 2 — Description of Additional Supplementary Files [file 41467_2025_59147_MOESM2_ESM.pdf]

Description of Additional Supplementary Files

Supplementary Data 1 Differential Expressed Genes in human macrophages treated with ucenprubart or dupilumab compared to isotype treatment.
